# A Multicenter Study in Northern Italy to Evaluate the Impact of a Sepsis Bundle in Obstetric Settings: The SOS Study

**DOI:** 10.1093/ofid/ofaf337

**Published:** 2025-06-16

**Authors:** Marta Colaneri, Simona Biscarini, Lara Tiranini, Rebecca Pesare, Pietro Valsecchi, Elena Seminari, Arsenio Spinillo, Alessandra Bandera, Enrico Mario Ferrazzi, Andrea Gori, Laura Carenzi, Luigi Pusterla, Federico D’Amico, Alessandro Raimondi, Massimo Puoti, Elisa Vallicella, Gianpaolo Grisolia, Salvatore Casari, Alice Zavatta, Irene Cetin, Alice Bonetti, Marta Corbella, Fausto Baldanti, Patrizia Cambieri, Paola Brambilla, Catherine Klersy, Camilla Torriani, Maria Cristina Monti, Raffaele Bruno, Maternal Sepsis Group, Nicola Cesano, Veronica Bonaldo, Angelo Pan, Annalisa Abbiati

**Affiliations:** Department of Biomedical and Clinical Sciences, University of Milan, Milan, Italy; Centre for Multidisciplinary Research in Health Science, University of Milan, Milano, Italy; Infectious Diseases Unit, Fondazione IRCCS Ca’ Granda Ospedale Maggiore Policlinico, Milan, Italy; Department of Obstetrics and Gynecology, IRCCS Policlinico San Matteo Foundation, Pavia, Italy; S.C. Malattie Infettive, Fondazione IRCCS Policlinico San Matteo, Pavia, Italy; S.C. Malattie Infettive, Fondazione IRCCS Policlinico San Matteo, Pavia, Italy; S.C. Malattie Infettive, Fondazione IRCCS Policlinico San Matteo, Pavia, Italy; Department of Obstetrics and Gynecology, IRCCS Policlinico San Matteo Foundation, Pavia, Italy; Dipartimento di Scienze Clinico-Chirurgiche, Diagnostiche e Pediatriche Università di Pavia, Pavia, Italy; Infectious Diseases Unit, Fondazione IRCCS Ca’ Granda Ospedale Maggiore Policlinico, Milan, Italy; Department of Pathophysiology and Transplantation, University of Milan, Milan, Italy; Obstetrics Unit, Department of Mother, Child and Neonate, UOC Mangiagalli Fondazione IRCCS Ca’ Granda Ospedale Maggiore Policlinico, Milan, Italy; Department of Clinical Sciences and Community Health, University of Milan, Milan, Italy; Department of Biomedical and Clinical Sciences, University of Milan, Milan, Italy; Centre for Multidisciplinary Research in Health Science, University of Milan, Milano, Italy; Infectious Disease Unit II, Ospedale Luigi Sacco, ASST Fatebenefratelli Sacco, Milano, Italy; Infectious Diseases Unit, ASST Lariana Ospedale Sant’Anna, Como, Italy; Infectious Diseases Unit, ASST Lariana Ospedale Sant’Anna, Como, Italy; Infectious Diseases Unit, ASST Grande Ospedale Metropolitano Niguarda, Milan, Italy; Infectious Diseases Unit, ASST Grande Ospedale Metropolitano Niguarda, Milan, Italy; Infectious Diseases Unit, ASST Grande Ospedale Metropolitano Niguarda, Milan, Italy; Department of Obstetrics and Gynecology, Carlo Poma Hospital, ASST Mantova, Mantova, Italy; Department of Obstetrics and Gynecology, Carlo Poma Hospital, ASST Mantova, Mantova, Italy; Infectious Diseases Unit, ASST Ospedale Carlo Poma, Mantova, Italy; Department of Woman, Mother and Neonate, V. Buzzi Children Hospital, ASST Fatebenefratelli Sacco, Milan, Italy; Department of Biomedical and Clinical Sciences, University of Milan, Milan, Italy; Obstetrics Unit, Department of Mother, Child and Neonate, UOC Mangiagalli Fondazione IRCCS Ca’ Granda Ospedale Maggiore Policlinico, Milan, Italy; Microbiology and Virology Unit, IRCCS Fondazione Policlinico San Matteo, Pavia, Italy; Microbiology and Virology Unit, IRCCS Fondazione Policlinico San Matteo, Pavia, Italy; Dipartimento di Scienze Clinico-Chirurgiche, Diagnostiche e Pediatriche Università di Pavia, Pavia, Italy; Microbiology and Virology Unit, IRCCS Fondazione Policlinico San Matteo, Pavia, Italy; Microbiology and Virology Unit, IRCCS Fondazione Policlinico San Matteo, Pavia, Italy; Infectious Diseases Unit, ASST Ospedale of Cremona, Cremona, Italy; SSD Biostatistica e Clinical Trial Center, Fondazione IRCCS Policlinico San Matteo, Pavia, Italy; Unit of Biostatistics and Clinical Epidemiology, Department of Public Health, Experimental and Forensic Medicine, University of Pavia, Pavia, Italy; Unit of Biostatistics and Clinical Epidemiology, Department of Public Health, Experimental and Forensic Medicine, University of Pavia, Pavia, Italy; S.C. Malattie Infettive, Fondazione IRCCS Policlinico San Matteo, Pavia, Italy; Dipartimento di Scienze Clinico-Chirurgiche, Diagnostiche e Pediatriche Università di Pavia, Pavia, Italy

**Keywords:** bundle, maternal sepsis, neonatal health, pregnancy, puerperium

## Abstract

**Background:**

In 2018, Lombardy's Fight Against Sepsis in Obstetrics group developed a regional sepsis management bundle for obstetric patients. This study aimed to evaluate the impact of this bundle on maternal and neonatal clinical outcomes and on process measures.

**Methods:**

This multicenter, observational, retrospective study included data from pregnant and puerperal adult patients diagnosed with sepsis according to the Surviving Sepsis Campaign guidelines in 2 periods: May 2015 to May 2018 (prebundle) and July 2018 to January 2023 (postbundle).

**Results:**

Eighty women were included: 24 (30.0%) in the prebundle period and 56 (70.0%) in the postbundle period. The primary source of infection was urinary (40.0%), with *Escherichia coli* being the most common pathogen isolated from blood cultures. Regarding clinical outcomes, no deaths occurred in pre- and postbundle periods. For mothers, there was no significant difference in median length of stay between the groups, while neonatal intensive care unit admissions of neonates significantly decreased from 85.7% to 31.3% (*P* = .013). Regarding process measures, the only significant increase occurred in infectious disease specialist consultations in the postbundle period (75.0%) as compared with the prebundle period (50.0%, *P* = .029).

**Conclusions:**

The implementation of a regional maternal sepsis management bundle did not significantly alter maternal outcomes but was associated with a reduction in neonatal intensive care unit admissions, although what role bundle implementation had in this change remains uncertain. More infectious disease consultations postbundle highlight the potential role of the bundle increasing sepsis awareness among physicians dealing with these patients.

## Teaser

Sepsis is a significant cause of maternal deaths, but early identification and management remain challenging due to the unique physiologic changes in pregnancy. A sepsis management bundle specific to obstetrics was implemented in Lombardy, Italy, aiming to improve sepsis outcomes in pregnant and postpartum women. The study assessed the bundle's impact across 7 hospitals, finding no reduction in maternal intensive care unit admissions or length of hospital stay. The bundle did lead to a significant decrease in neonatal intensive care unit admissions and miscarriage rates, indicating improved clinician awareness of sepsis in this patient population. Despite the mixed results, the study suggests that improved guidelines and education could enhance sepsis management in this population.

Sepsis accounts for 11% of maternal deaths globally [1]; despite its significant impact, its early identification and management remain challenging for different reasons [2]. First, an international consensus definition of sepsis in pregnancy and puerperium was established by the World Health Organization only in 2017 [3]; second, pregnant women's physiologic and immunologic adaptations can alter their ability to effectively combat infection, obscure certain signs of sepsis, and delay its recognition [4]. In addition, sepsis guidelines have always been modeled on the basis of randomized controlled trials that did not include pregnant patients, affecting the utility of the Quick Sequential Organ Failure Assessment, National Early Warning Score, or Systemic Inflammatory Response Syndrome score in this unique population [5].

As a result, an obstetric-specific sepsis scoring system, the Modified Early Obstetric Warning Score (MEOWS), has been proposed and further validated [6, 7]. However, its efficacy to reduce maternal deaths seems limited [8], and this may depend on its potentially enhanced performance when coupled with a proper guidance on action enclosed in bundles.

Over the last 2 decades, the Surviving Sepsis Campaign highlighted the importance of generating standardized guidelines for management of sepsis and septic shock in the general population. The implementation of sepsis bundles aimed to direct physicians toward the early diagnosis and treatment of this condition, leading to an improvement in the quality of management for cases of sepsis [9].

The evidence surrounding the efficacy of sepsis bundles remains inconclusive, with conflicting data emerging from recent studies. While the Sepsis Six, a set of 6 practical interventions, is widely recognized for its potential to significantly improve survival rates when implemented within the first hour of sepsis recognition [10], recent multicenter randomized trials have shown that early goal-directed therapy may not offer benefits over usual care [11–13]. Importantly, there is a significant gap in the literature regarding the application and effectiveness of sepsis bundles in the pregnant and puerperal populations, where sepsis presents unique challenges [14].

In May 2018, the Fight Against Sepsis in Obstetrics working group of the Lombardy Region in Northern Italy developed a bundle following regional risk management indications, which was aimed at proposing definitions and procedures for the identification and management of sepsis in the obstetric setting [15]. The objective of this study was to assess the effects of the regional sepsis bundle implementation in 7 hospitals in Lombardy.

## METHODS

### Study Design and Setting

The SOS study is a multicenter, observational, and retrospective cohort study conducted in 7 referral hospitals in the Lombardy Region, Northern Italy (IRCCS Fondazione Policlinico San Matteo of Pavia, Azienda Socio-Sanitaria Territoriale of Cremona, Ospedale Sant’Anna of Como, IRCCS Ospedale Ca’ Granda of Milan, ASST Grande Ospedale Metropolitano Niguarda of Milan, Ospedale Carlo Poma of Mantova, Vittore Buzzi Children Hospital of Milan).

The leading coordinating center was IRCCS Policlinico San Matteo Foundation, Pavia. Every center housed intensive care unit (ICU), infectious diseases (ID), and gynecology/obstetrics beds. Moreover, all the centers had a specific obstetrical emergency department, as well as pediatric and neonatology specialists.

### Inclusion Criteria

Data were collected of all the pregnant and puerperal adult patients who were diagnosed with sepsis according to the Surviving Sepsis Campaign guidelines [9] between May 2015 and May 2018 (prebundle period) and between July 2018 and January 2023 (postbundle period). The only exclusion criterion was age <18 years. The reason for the 2-month gap was to allow time for the bundle to circulate and be used in daily practice by the considered hospitals in Lombardy.

First, medical records were retrieved of all pregnant and puerperal patients discharged with a sepsis-related *ICD-9* code—namely, regarding “sepsis,” “septic shock,” and “infection.” Second, clinical data were analyzed by ID specialists, and the patients were included if they had (1) organ dysfunction, determined by an increase ≥2 points in the Sequential Organ Failure Assessment (SOFA) score, and (2) infection, the diagnosis of which relied on clinical suspicion from signs and symptoms of infection, radiologic and microbiologic data, and response to therapy. The medical records were then anonymized and abstracted on standardized data collection forms in the web platform REDCap. Demographic and clinical data were retrospectively extracted from paper and electronic records from the 7 involved hospitals. Notably, the centers were eligible for the study if caring for at least 1500 deliveries per year.

### Patient Consent

This research project was carried out in accordance with a research plan and per the current version of the Declaration of Helsinki. The sponsor (IRCCS Policlinico San Matteo Foundation, Pavia) ensured that approval from a competent ethics committee was sought for the study. Informed consent for pseudonymized data processing for future research purposes was provided by all patients at the time of hospital admission, as routine procedure. Specific written informed consent was waived because of the retrospective nature of the analysis. The SOS study was approved by the IRCCS Policlinico San Matteo Foundation Institutional Review Board (20210080285; 25 August 2021).

### Bundle Description

The “Operational Guidance Document for Early Identification and Management of Sepsis in Obstetrics” was developed by the working group Fight Against Sepsis in Obstetrics of the Lombardy Region, Northern Italy, following regional indications of risk management. A version translated in English is provided in the supplementary materials. The aim of the bundle was to propose definitions and procedures for the identification and management of sepsis in the obstetric population.

Although this is a comprehensive bundle that addresses various aspects of sepsis management, such as hemodynamic support, fluid resuscitation, and vasopressor use, our primary focus was related to ID-centered aspects, such as recognition and management of maternal sepsis with appropriate microbiological testing and antibiotic therapy. We briefly discuss these key points in turn.

#### Recognition of Maternal Sepsis

The bundle clearly states that all pregnant women arriving at the maternity triage service or already hospitalized should be monitored with the MEOWS, a specific early warning tool (Supplementary Figure 1, supplementary materials). MEOWS entails the assessment of vital signs such as respiratory rate, blood pressure, oxygen saturation, level of consciousness, and urinary output. Each parameter deviation from normal values is assigned a risk zone indicated by the colors white, yellow, and red. MEOWS enables early identification of patients with objective signs of potential ongoing infection but is insufficient for sepsis diagnosis. Maternal sepsis diagnosis requires the presence—during pregnancy or childbirth, postabortion, or postpartum—of a presumed or confirmed infection associated with damage to 1 or more organs, as evidenced by specific criteria, such as oxygen requirement to maintain SpO_2_ >95% or PaO_2_/FiO_2_ <400, platelet count <100 × 10^6^/L, bilirubin level >1.2 mg/dL, systolic blood pressure <90 mm Hg or mean arterial pressure <75 mm Hg, patient arousable to stimuli (verbal, painful, or unconscious), and creatinine level >1.2 mg/dL. The presence of 1 or more of these organ damage criteria corresponds to a modified SOFA (mSOFA) score ≥1.

#### Management of Maternal Sepsis

**Figure 1.**
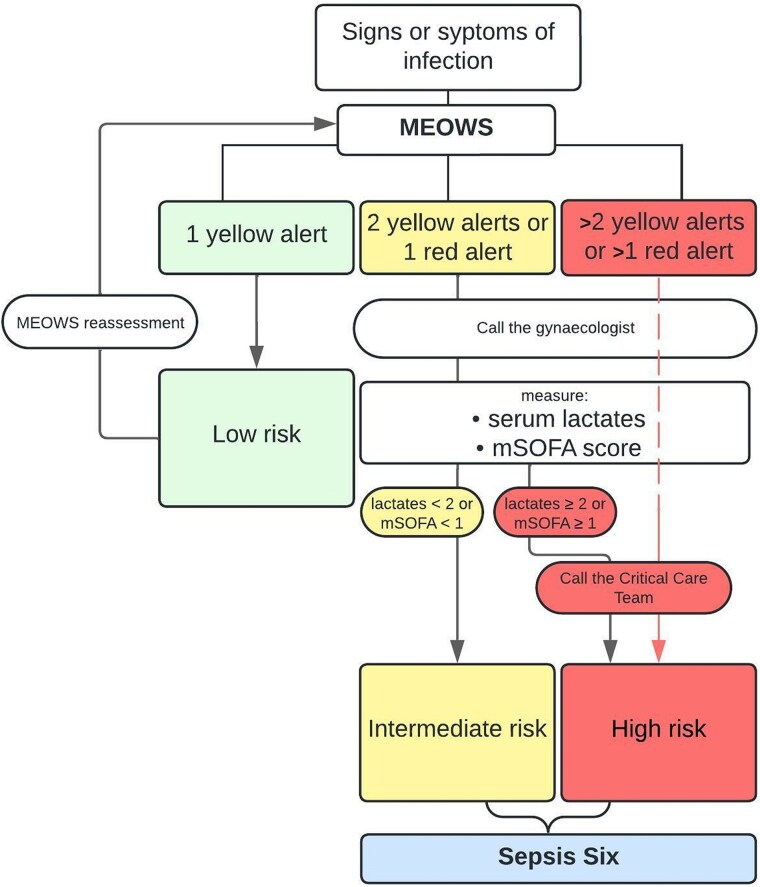
Definition of risk zone based on the Modified Early Obstetric Warning Score (MEOWS) and the modified Sequential Organ Failure Assessment (mSOFA). The image is the modified English version of a flowchart included in the “Operational Guidance Document for Early Identification and Management of Sepsis in Obstetrics” (supplementary materials, attachment 1). The Sepsis Six consists of 3 diagnostic and 3 therapeutic steps—all to be delivered within 1 hour of the initial diagnosis of sepsis:1. Deliver high-flow oxygen.2. Take blood cultures and consider source control.3. Administer empiric intravenous antibiotics.4. Measure serial serum lactates and send full blood count.5. Start intravenous fluid resuscitation.6. Commence accurate urine output measurement [10].

Depending on MEOWS, mSOFA, and lactate values obtained through arterial blood examination of patients, different “risk zones” are identified, as depicted in Figure 1, outlining the appropriate pathway to follow. In particular, implementation of the Sepsis Six panel is necessary in all cases of intermediate risk (infection signs/symptoms + 2 yellow or 1 red MEOWS alert) and high risk (infection signs/symptoms + >2 yellow or >1 red MEOWS alert or mSOFA ≥1).

The bundle provides detailed instructions for performing cultures before antibiotics, recommending at least 2 sets of blood cultures (BCs) and the search for infection foci. Regarding antibiotics, intravenous administration within the first hour is recommended in suspected or confirmed cases of sepsis, and the combination of penicillin/β-lactamase inhibitor is suggested as the first choice in the treatment, considering the most common septic sources in the obstetric context.

With the bundle, there were educational activities that consisted of formal training sessions, educational meetings, and visual aids such as posters in emergency departments and patient care areas. Additionally, a paper MEOWS parameter collection form was introduced in all patient charts to improve the early recognition of sepsis.

### Patient Characteristics

The demographic data included age and ethnicity. Clinical data included comorbidities (history of cancer, chronic kidney disease, chronic liver disease, autoimmune disease, and hematologic disease). Obstetric data comprised gestational age, type of delivery, and delivery complications. Sepsis data included the timing of sepsis identification (antenatal, peripartum, or in the puerperium), MEOWS and mSOFA scores, and lactate values.

Although a definition of pregnant patients at high risk has always been difficult to assess [16], according to Zhang et al [17] we defined this subgroup of patients as those who had at least 1 of the following conditions: age ≥40 years, prepregnancy body mass index ≥30, history of drug or alcohol abuse, arterial hypertension, cardiovascular disease, history of arterial or venous thromboembolic events, pregestational diabetes, renal disease, rheumatic disease, thyroid abnormalities, hematologic disease, pulmonary disease, psychiatric disorders, cancer, gestational hypertension, intrahepatic cholestasis of pregnancy, previous cesarean sections, and history of thromboembolic events during pregnancy.

### Microbiological and Treatment Data

Microbiological data included the type of microorganism causing the infection with specific susceptibility profiles, the site of infections, and cultures of specific samples (eg, fetus, amniotic liquid, and placenta). BCs were defined positive according to Weinstein et al [18]. A positive BC result was defined polymicrobial when more than 1 pathogen was isolated from the same blood sample or from 2 consecutive blood samples of the same patient within 24 hours.

Coagulase-negative staphylococci, aerobic and anaerobic diphtheroid, *Micrococcus* spp, *Bacillus* spp, and viridans streptococci were considered contaminants if only 1 bottle of BC was positive. In this case, the isolate was reported as a probable contaminant, and susceptibility testing was not performed.

Bacterial isolates were defined as multidrug-resistant organisms when nonsusceptible to at least 1 agent in ≥3 antimicrobial categories or when harboring specific antibiotic resistance mechanisms (eg, methicillin-resistant *Staphylococcus aureus*, vancomycin-resistant *Enterococcus faecium*, and extended-spectrum β-lactamase– or carbapenemase-producing Enterobacterales) [19]. Treatment data included the type of antibiotic, timing of administration, and total duration of the therapy.

To assess the appropriateness of antibiotic therapy, we reviewed all infections and evaluated whether the administered empiric antibiotic regimen was aligned with the spectrum of activity required for the subsequently isolated pathogen.

### Outcomes Data

The outcomes data were structured into patient-centered outcomes and process measures, reflecting clinical impacts and key intervention metrics [20]. Patient-centered data encompassed a comprehensive assessment of maternal and neonatal health. For the mothers, the incidence of maternal death, admission to ICU, and length of stay (LOS) were evaluated.

The following were assessed for neonates: miscarriage (fetal death before 24 weeks of gestation), stillbirth (fetal death at ≥24 weeks of gestation), weight at birth, admission to the neonatal ICU (NICU), and early neonatal death (defined as mortality within the first 7 days of life). Process measures comprised the rate of lactate measurements, the rate of positive BC results, the time elapsed between the suspicion of sepsis and BC collection, the administration of antibiotics before sepsis suspicion and before proper collection of BCs, the appropriateness of empirical antibiotic therapy, the length of antibiotic therapy, and the frequency of requests for ID specialist consultations.

### Objectives

The primary objective of this study was to assess whether the bundle implementation influenced the median LOS of the patients. Specifically, it aimed to determine if there was a decrease in LOS during the postbundle period as compared with the prebundle period.

Secondary objectives of the study were as follows:

- To evaluate if there was a reduction in sepsis-related ICU admissions of patients during the postbundle period as compared with the prebundle period.
- To assess whether there was a decrease in the number of neonatal deaths or admissions to the NICU during the postbundle period as compared with the prebundle period.
- To determine if there was an increased request of lactates in the suspected cases of maternal sepsis in the postbundle period as compared with the prebundle period.
- To determine if there was an increased rate of BCs requested in the postbundle period as compared with the prebundle period.
- To examine if the time interval between the suspicion of sepsis and BC collection improved after the bundle implementation.
- To determine if there was a reduction in the rate of antibiotics initiated before BC collection.
- To determine if there was an increase of the appropriateness of antibiotic therapy in the postbundle period as compared with the prebundle period.
- To determine if the length of antibiotic therapy was shorter in the postbundle period as compared with the prebundle period.
- To investigate whether there was a significant rise in the number of ID consultations postbundle.

### Sample Size Calculation

From a clinical perspective, we were primarily interested in evaluating how the implementation of the bundle influenced patient clinical outcomes. Since maternal sepsis remains a rare event and severe outcomes such as mortality are fortunately even less frequent, the choice of LOS as the primary outcome of this study was driven by methodological considerations, particularly the need to ensure an adequate sample size. Therefore, although we recognize the inherent limitations of LOS as an outcome, as it may be affected by multiple factors beyond infection management, we believe that it provides valuable insight into the overall impact of the bundle on maternal care.

Assuming an initial mean hospital stay of 14 days (SD, 2), we anticipate a clinically meaningful reduction of 2 (2) days in the postbundle group. Based on these estimates, a minimum of 52 patients (26 in each group) is required to detect this difference with 80% power while maintaining a type I error rate of 1% (α = .01). This sample size was determined through power analysis, ensuring that our study is adequately powered to identify changes in patient outcomes attributable to the bundle intervention. The estimated patient count is consistent with the catchment population and expected incidence of sepsis in pregnancy in the participating hospitals.

### Statistical Methods

Median and IQR were used to describe continuous variables. Categorical variables were represented with absolute frequencies and percentages.

Appropriate statistical tests were conducted to compare variables between the pre- and postbundle groups, selected per the nature of the variables. Fisher exact test or χ^2^ test was applied for categorical variables, as appropriate, after checking for violation of Pearson χ^2^ expected frequencies assumption. For continuous variables, the Wilcoxon rank sum test was employed to compare the distributions between the groups.

Statistical significance was fixed for *P* values <.05. Statistical analyses were conducted with R (version 4.2.1).

## RESULTS

**Figure 2.**
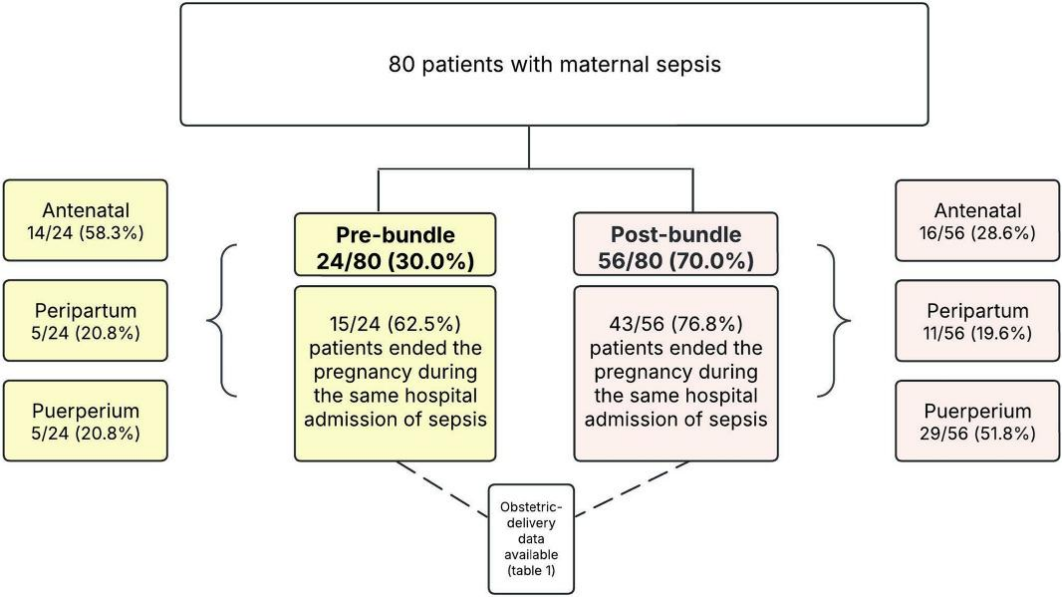
Flowchart of study population.

Overall, 80 women with sepsis during pregnancy or puerperium were included, with a median age of 31.0 years (IQR, 26.0–36.3). Among them, 24 (30.0%) were evaluated in the prebundle period and 56 (70.0%) in the postbundle period (Figure 2).

Regarding the history of these patients, few underlying comorbidities were reported, with thyroid abnormalities being the most frequent (13/80, 16.3%). Moreover, 9 (11.3%) patients previously underwent abdominal surgery. Seven (8.8%) patients had a penicillin allergy, while 3 (3.8%) already had a septic event.

Regarding previous pregnancies, 42 (52.5%) patients were primiparas while 16 (20.0%) had at least 1 previous cesarean section. More than half of patients (52/80, 65%) had a high-risk pregnancy: 15 of 24 (63%) in the prebundle period and 37 of 56 (66%) in the postbundle period (*P* = .759). Eight (10.0%) patients had sepsis during or following a multiple pregnancy, while the most frequent pregnancy complication was premature preterm rupture of membranes (16/80, 20.0%).

Delivery data were collected from 58 patients (15 prebundle and 43 postbundle) who ended their pregnancy during the same hospital admission of the septic event. Specifically, most patients underwent a cesarean section (n = 35, 60.3%) while 20 (34.5%) needed an induction of labor. In available medical records, 14 (30.4%) patients received an antibiotic prophylaxis in the peripartum period.

Gestational age at the time of delivery was lower in prebundle patients as compared with postbundle, with a median 26.0 weeks (IQR, 17.0–33.0) vs 37.0 (27.0–39.8; *P* = .006). Concerning the delivery complications, nearly a third of women (15/55, 27.3%) experienced a postpartum hemorrhage while 14 of 53 (26.4%) underwent manual removal of retained placenta. Demographic, clinical, and obstetric-delivery data of the study cohort are reported in Table 1.

**Table 1.** Demographic, Clinical, and Obstetric-Delivery Data

		No. (%) or Median (IQR)			
	No.	Overall (n = 80)	Prebundle (n = 24)	Postbundle (n = 56)	*P* Value^a^
Age, y	80	31.0 (26.0–36.3)	33.5 (25.8–37.3)	31 (26.0–36.0)	.578
Ethnicity	79				.301
Caucasian		53 (66.3)	15 (62.5)	38 (67.9)	
Hispanic		6 (7.5)	1 (4.2)	5 (8.9)	
Indian		3 (3.8)	1 (4.2)	2 (3.6)	
Asiatic		2 (2.5)	2 (8.3)	0 (0)	
African		6 (7.5)	1 (4.2)	5 (8.9)	
Arabic		7 (8.8)	2 (8.3)	5 (8.9)	
Other		2 (2.5)	0 (0)	2 (3.6)	
History	80				
Penicillin allergy		7 (8.8)	3 (13)	4 (7.1)	.450
Cardiovascular disease		3 (3.8)	1 (4.2)	2 (3.6)	>.981
Thyroid abnormalities		13 (16.3)	6 (25.0)	7 (12.5)	.200
Hematologic malignancy		4 (5.0)	0 (0)	4 (7.1)	.304
Respiratory disease		2 (2.5)	0 (0)	2 (3.6)	>.900
Kidney disease		1 (1.3)	0 (0)	1 (1.8)	>.900
Rheumatic disease		2 (2.5)	1 (4.2)	1 (1.8)	.577
Previous septic event		3 (3.8)	2 (8.3)	1 (1.8)	.232
Uterine fibroids		5 (6.6)	1 (4.2)	4 (7.7)	>.900
Previous abdominal surgery		9 (11.3)	2 (8.3)	7 (12.5)	.734
Obstetric history					
No. of prior pregnancies	80				.400
0		42 (53.8)	10 (41.7)	32 (57.1)	
≥3		7 (8.7)	3 (12.5)	4 (7.1)	
Previous cesarean section	79	16 (20.0)	4 (16.7)	12 (21.4)	>.900
Multiple pregnancy	80	8 (10.0)	1 (4.2)	7 (12.5)	.401
Pregnancy complication					
HELLP syndrome	80	3 (3.8)	1 (4.2)	2 (3.6)	>.900
Gestational diabetes	80	5 (6.3)	1 (4.2)	4 (7.1)	>.900
Gestational hypertension	80	8 (10.0)	1 (4.2)	7 (12.5)	.458
Cholestasis	80	3 (3.8)	0 (0)	3 (5.4)	.587
PPROM	80	16 (20.0)	5 (20.8)	11 (19.6)	>.900
Preterm labor	79	9 (11.4)	4 (16.7)	5 (9.1)	.411
Patients at high risk	80	52 (65)	15 (63)	37 (66)	.759
Obstetric-delivery data ^b^		58	15	43	
Gestational age delivery, wk	57	36.0 (25.0–39.0)	26.0 (17.0–33.0)	37.0 (27.0–39.8)	.006
Induction of labor	58	20 (34.5)	4 (26.7)	16 (37.2)	.500
Cesarean section	58	35 (60.3)	10 (66.7)	25 (58.1)	.577
Antibiotic prophylaxis	46	14 (30.4)	6 (50.0)	8 (23.5)	.140
Delivery complications					
Postpartum hemorrhage	55	15 (27.3)	2 (14.3)	13 (31.7)	.301
Manual removal of retained placenta	53	14 (26.4)	4 (30.8)	10 (25.0)	.678
Disseminated intravascular coagulation	55	2 (3.6)	1 (7.1)	1 (2.4)	.400

Abbreviations: HELLP, hemolysis, elevate liver enzymes, and low platelet count; PPROM, premature preterm rupture of membranes.

^a^Fisher exact test, Wilcoxon rank sum test, Pearson χ^2^ test.

^b^Data are provided for patients who ended pregnancy during the same hospital admission as the septic event.

Overall, sepsis occurred in 30 patients (37.5%) during the antenatal period, 16 (20.0%) during the peripartum period, and 34 (42.5%) during the puerperium. In the postbundle period, there was a significant increase in postpartum cases and a decrease in cases during pregnancy (*P* = .021). Data on sepsis are presented in Table 2.

**Table 2.** Sepsis Data

		No. (%) or Median (IQR)			
	No.	Overall (n = 80)	Prebundle (n = 24)	Postbundle (n = 56)	*P* Value^a^
Time of sepsis identification	80				.021
Antenatal: from conception to labor onset		30 (37.5)	14 (58.3)	16 (28.6)	
Peripartum: from labor onset to delivery of placenta		16 (20.0)	5 (20.8)	11 (19.6)	
Puerperium: from delivery of placenta to 42 d after		34 (42.5)	5 (20.8)	29 (51.8)	
Source of sepsis					
Genital tract	80	12 (15.0)	5 (20.8)	7 (12.5)	.500
Urinary tract	80	32 (40.0)	9 (37.5)	23 (41.1)	.765
Chorioamnionitis	80	23 (28.8)	8 (33.3)	15 (26.8)	.588
Pneumonia	80	7 (8.8)	3 (12.5)	4 (7.1)	.357
Appendicitis	80	1 (1.3)	0 (0)	1 (1.8)	>.900
Surgical site	80	6 (7.5)	1 (4.2)	5 (8.9)	.701
Skin soft tissue	80	1 (1.3)	0 (0)	1 (1.8)	>.900
Mastitis	80	1 (1.3)	0 (0)	1 (1.8)	>.900
Unknown	80	8 (10.0)	0 (0)	8 (14.3)	.100
MEOWS: color-coded score risk	42				.310
High		26 (61.9)	…	26 (61.9)	
Intermediate		12 (28.6)	…	12 (28.6)	
Low		4 (9.5)	…	4 (9.5)	
mSOFA score	73				.800
<1		22 (30.1)	7 (31.8)	15 (29.4)	
≥1		51 (69.9)	15 (68.2)	36 (70.6)	
Laboratory test results					
Lactates	38	1.5 (0.8–2.7)	0.9 (0.7–1.5)	1.6 (0.8–2.7)	.199
White blood cells count, × 10^3^/μL	79	13.4 (8.0–18.3)	10.2 (3.5–15.4)	14.9 (9.1–19.5)	.050
C-reactive protein, mg/dL	79	14.7 (7.8–21.9)	11.8 (8.1–19.0)	15.4 (7.8–23.3)	.186
Diagnostic imaging performed	80				
Chest x-ray		42 (52.5)	14 (58.3)	28 (50.0)	.469
Abdominal ultrasound		47 (58.8)	12 (50.0)	35 (62.5)	.269
Transvaginal ultrasound		42 (52.5)	12 (50.0)	30 (53.6)	.799
CT scan		20 (25.0)	7 (29.2)	13 (23.2)	.622

Abbreviations: CT, computed tomography; MEOWS, Modified Early Obstetric Warning Score; mSOFA, modified Sequential Organ Failure Assessment.

^a^Fisher exact test, Wilcoxon rank sum test, Pearson χ^2^ test.

The primary source of infection was urinary (n = 32, 40.0%), followed by chorioamnionitis (n = 23, 28.8%) and genital tract infections (n = 12, 15.0%). Regarding disease severity, 51 (69.9%) of 73 patients had a mSOFA ≥1, while among those in the postbundle period group, 38 of 42 (90.5%) had a high- or intermediate-risk score on MEOWS. In terms of imaging, slightly more than half of the patients (n = 42, 52.5%) underwent ultrasound or x-ray, while 20 (25.0%) required a computed tomography scan. Table 3 shows the microbiological data.

**Table 3.** Blood Cultures Results and Treatment Details

		No. (%)			
	No.	Overall (n = 80)	Prebundle (n = 24)	Postbundle (n = 56)	*P* Value^a^
Polymicrobial	52^b^	4 (7.7)	0 (0)	4 (10.3)	.677
Isolated pathogens	56^b^	56	13	43	.689
* Escherichia coli*		27 (48.2)	8 (61.5)	19 (44.2)	
* Klebsiella pneumoniae*		11 (19.6)	2 (15.4)	9 (20.9)	
Anaerobic bacteria^c^		6 (10.7)	1 (7.7)	5 (11.6)	
* Streptococcus* spp^d^		6 (10.7)	1 (7.7)	5 (11.6)	
* Enterococcus* spp^e^		3 (5.4)	0 (0)	3 (6.9)	
* Staphylococcus* spp^f^		3 (5.4)	1 (7.7)	2 (4.7)	
Class of administered antibiotics					
Penicillins	80	51 (63.8)	12 (50.0)	39 (69.6)	.094
Cephalosporins	78	28 (35.9)	10 (41.7)	18 (33.3)	.455
Carbapenems	77	21 (27.3)	5 (20.8)	16 (30.2)	.366
Fluoroquinolones	77	7 (9.1)	2 (8.3)	5 (9.4)	>.900
Aminoglycosides	79	24 (30.4)	4 (16.7)	20 (36.4)	.081
Glycopeptides and lipoglycopeptides	79	17 (21.5)	5 (20.8)	12 (21.8)	>.900
Macrolides, lincosamides	80	10 (12.5)	6 (25.0)	4 (7.1)	.058
Others	79	17 (21.5)	9 (37.5)	8 (14.5)	.022

No vancomycin-resistant *Enterococcus faecium* or extended-spectrum β-lactamase/AmpC/carbapenemase-producing Enterobacterales was found.

^a^The Wilcoxon rank sum test and Pearson χ^2^ test are used to compare the presence or absence of specific administered antibiotics between the pre- and postperiods.

^b^Blood cultures were performed in 74 of 80 patients (92.5%); among them, 52 of 74 (70.3%) cultures were positive and 56 different pathogens were isolated.

^c^Anaerobic includes gram-negative bacteria as *Bacteroides fragilis* (1/6, 17%), *Parabacteroides distasonis* (1/6, 17%), and *Prevotella bivia* (1/6, 17%), as well as gram-positive bacteria as *Peptostreptococcus* spp (2/6, 33%) and facultative anaerobic as *Gardnerella vaginalis* (1/6, 17%).

^d^ *Streptococcus* spp include *S agalactiae* (2/6), *S anginosus* (1/6), *S gallolyticus* (1/6), *S mitis* (1/6), and *S pyogenes* (1/6).

^e^ *Enterococcus* spp include *E faecalis* (2/3, 66%) and *E faecium* (1/3, 33%).

^f^ *Staphylococcus* spp include methicillin-sensitive *S aureus* (1/3, 33%), methicillin-resistant *S aureus* (1/3, 33%), and methicillin-resistant *S epidermidis* (1/3, 33%).

Among the 52 positive BC results, 56 pathogens were identified, with 4 of 52 (7.7%) showing a polymicrobial infection. The most frequently isolated bacteria were Enterobacterales, namely *Escherichia coli* (27/56 48.2%) and *Klebsiella pneumoniae* (11/56, 19.6%), followed by anaerobic bacteria (6/56, 10.7%) and gram-positive bacteria such as *Streptococcus* spp (6/56, 10.7%), *Enterococcus* spp (3/56, 5.4%), and *Staphylococcus* spp (3/56, 5.4%). Multidrug-resistant organisms accounted only 2 (3.6%) of the 56 isolated pathogens: 1 methicillin-resistant *S aureus* and 1 methicillin-resistant *Staphylococcus epidermidis*. No vancomycin-resistant *E faecium* or extended-spectrum β-lactamase/AmpC/carbapenemase–producing Enterobacterales was found.

Details on microbial isolates in urine, vaginal swab, placenta, and fetus/newborn cultures are reported in Supplementary Table 1 and the supplementary materials (attachment 2). Most patients (51/80, 63.8%) received penicillins (primarily piperacillin/tazobactam) as monotherapy or in combination as empiric antibiotic therapy.

### Patient-Centered Outcomes

Regarding the primary objective of the study, the median LOS was 13.0 days (IQR, 9.0–18.3), with no differences between the periods. Notably, no maternal deaths or stillbirths occurred in either group. Of 80 patients, 22 (27.5%) required ICU admission, with 9 patients (37.5%) in the prebundle period and 13 (23.2%) in the postbundle period. Although there was a reduction in the number of ICU admissions in the postbundle period, this difference was not statistically significant (*P* = .200). Of 58 pregnancies, 15 (25.9%) ended in miscarriages: 7 of 15 (46.7%) in the prebundle period and 8 of 43 (18.6%) in the postbundle (*P* = .045). A significantly lower percentage of newborns required NICU admission after the introduction of the bundle, with 6 of 7 (85.7%) and 10 of 32 (31.3%) in the pre- and postbundle periods, respectively (*P* = .013). Early neonatal death (within 7 days) occurred in 3 newborns (7.7%) for the overall cohort.

### Process Measures

Regarding the secondary objectives and the process measures, the lactate measurement was assessed in 80 patients overall, with an increase in the postbundle period (52%) as compared with the prebundle period (38%), but this difference was not statistically significant (*P* = .200). Similarly, the rate of BCs requested increased in the postbundle period (75%) vs the prebundle period (59%, *P* = .222). Following the same trend, the median time between suspected sepsis and BC collection improved from 11.0 hours (IQR, 0.0–28.0) prebundle to 2.0 hours (0.8–21.3) postbundle, but no significant difference was observed (*P* = .800). Importantly, more than half of the patients (51.9%) were already undergoing antibiotic treatment before sepsis suspicion, and 65.8% had received antibiotics before BC collection, with no significant difference between the pre- and postbundle periods.

Empirical antibiotic therapy was appropriate in 75% of cases overall, increasing from 69% in the prebundle group to 77% in the postbundle group, although this change was not statistically significant. Moreover, the median duration of treatment was similar in the 2 groups, with 8.0 days (IQR, 7.0–14.0) prebundle and 11.0 days (7.3–16.0) postbundle (*P* = .297).

Finally, there was a significant increase in ID specialist consultations in the postbundle period (75.0%) as compared with the prebundle period (50.0%, *P* = .029). Table 4 reports details about treatment and outcome data, and Table 5 reports details about the process measure outcomes.

**Table 4.** Patient-Centered Outcomes

	No.	Overall (n = 80)	Prebundle (n = 24)	Postbundle (n = 56)	*P* Value^a^
Length of stay, d	80	13.0 (9.0–18.3)	10.5 (8.0–17.0)	14.0 (10.0–20.3)	.211
Maternal death	0	0 (0)	0 (0)	0 (0)	…
ICU admission	80	22 (27.5)	9 (37.5)	13 (23.2)	.200
ICU length of stay, d	22	3.5 (2.3–6.8)	3.0 (2.0–6.0)	5.0 (3.0–7.0)	.300
Miscarriage	58	15 (25.9)	7 (46.7)	8 (18.6)	.045
Stillbirth	58	0 (0)	0 (0)	0 (0)	…
NICU admission	39	16 (41.0)	6 (85.7)	10 (31.3)	.013
Weight at birth, g	38	2948.5 (1957.5–3422.5)	1100.0 (988.5–3100.0)	3100.0 (2202.5–3437.0)	.300
Early neonatal death, within 7 d	39	3 (7.7)	1 (14.2)	2 (6.3)	.522

Abbreviations: ICU, intensive care unit; NICU, neonatal intensive care unit admission.

^a^The Wilcoxon rank sum test and Pearson χ^2^ test are used to compare the presence or absence of specific administered antibiotics between the pre- and postperiods.

**Table 5.** Sepsis Bundle Process Measures

		No. (%) or Median (IQR)			
	No.	Overall (n = 80)	Prebundle (n = 24)	Postbundle (n = 56)	*P* Value^a^
Lactates performed	80	38 (48)	9 (38)	29 (52)	.200
Blood cultures performed	80	74 (92.5)	22 (91.7)	52 (92.9)	.222
Positive blood culture results	74	52 (70.3)	13 (59.0)	39 (75.0)	.222
Time elapsed between suspicion of sepsis and blood culture, h	50	2.7 (0.7–23.3)	11.0 (0.0–28.0)	2.0 (0.8–21.3)	.800
Antibiotic treatment before sepsis suspicion	79	41 (51.9)	11 (47.8)	30 (53.6)	.566
Antibiotic started before cultures	76	50 (65.8)	13 (59.0)	37 (68.6)	.379
Antibiotic appropriateness	52	39 (75)	9 (69)	30 (77)	.700
Length of antibiotic treatment, d	78	10.0 (7.0–15.8)	8.0 (7.0–14.0)	11.0 (7.3–16.0)	.297
Infectious disease specialist consultation	80	54 (67.5)	12 (50.0)	42 (75.0)	.029

^a^The Wilcoxon rank sum test and Pearson χ^2^ test are used to compare the presence or absence of specific administered antibiotics between the pre- and postperiods.

## DISCUSSION

During the study period, a total of 80 patients developed maternal sepsis, with 50 cases occurring in the peripartum and puerperium periods. The primary infection source was urinary, predominantly caused by *E coli*, and penicillins were the most frequently prescribed antibiotics. These findings align with existing data from high-income countries, where *E coli* is a leading pathogen in maternal sepsis, with urinary and genital tract infections being most common [21, 22]. Similarly, World Health Organization data from the GLOSS group confirm these findings, suggesting that cephalosporins and amoxicillin are commonly prescribed in similar populations [23]. In light of these consistent findings, our study contributes to a growing understanding of pathogen patterns and treatment practices in maternal sepsis, highlighting a critical area for focused intervention.

The bundle did not significantly reduce maternal LOS or ICU admissions. However, there was a reduction in NICU admissions observed, although the contribution of the bundle implementation to this change remains uncertain. Given the different median gestational age between pre- and postimplementation groups (26 vs 37 weeks), it is possible that preterm monitoring requirements contributed to fetal outcome differences. These differences between the populations may be due to chance but also could be seen as an effect of the bundle itself in that it may have standardized early recognition of sepsis across gestational ages, potentially shifting clinical focus beyond high-risk preterm cases. We believe that this potential change in focus may represent an important but often overlooked advantage of bundles—namely, improving overall sepsis awareness and response rather than merely targeting high-risk groups [24].

Moreover, the significant increase in sepsis diagnoses during the postpartum period and the corresponding reduction in cases during pregnancy in the postbundle group, along with differences in sample sizes and population characteristics, complicate the attribution of observed improvements solely to the bundle. The postbundle cohort was larger and coincided with heightened clinical awareness, which may have influenced outcomes beyond the bundle's direct impact. This also suggests that while standardized protocols are beneficial, their success is contingent upon institutional engagement, clinician adherence, and preexisting clinical culture [25].

To assess the impact of the sepsis bundle, key process-specific metrics were analyzed. Despite a nonsignificant improvement in several process measures, the observed trend suggests a positive evolution of diagnostic and management strategies. Notably, ID consultations significantly increased in the postbundle period, reflecting the explicit recommendation in the bundle to involve ID specialists early. In fact, prompt ID involvement has been already recognized as a key factor in improving sepsis outcomes in other populations [26].

Antibiotic appropriateness remained unchanged between the pre- and postbundle groups despite the bundle's focus on antimicrobial stewardship. This aligns with other studies highlighting challenges in achieving consistent antibiotic adherence in sepsis management [27]. Continued efforts, including real-time feedback and clinician education, remain crucial for improving antimicrobial stewardship [28]. The persistent issue of inappropriate microbiological sampling timing, with more than half of the patients receiving antibiotics before BC collection, underscores the need for further optimization of bundle adherence. This gap, despite bundle implementation, reflects ongoing difficulties in prioritizing BCs before antibiotic initiation, as noted in the literature [29].

This study has several important limitations. The retrospective design limits control over confounding variables, and data collection from 7 hospitals introduces variability in documentation practices. The prebundle sample size (n = 24) was slightly below the target of 26 but remained close to the original estimate, and a post hoc power analysis confirmed sufficient statistical power. Additionally, differences in population characteristics between periods suggest that improved neonatal outcomes cannot be attributed solely to bundle implementation but may reflect evolving clinical practices.

Despite these limitations, this study provides valuable insights. It is among the first to examine the effects of the Surviving Sepsis Campaign guidelines on maternal and neonatal outcomes, addressing a gap in existing literature. Implementing a structured bundle across multiple hospitals allowed for a comprehensive evaluation of sepsis management in pregnancy and puerperium. Moreover, a key aspect of the bundle's implementation was the adoption of multiple educational strategies to enhance guideline adherence, including the awareness of the potential benefit of increasing the ID specialists' involvement.

In conclusion, this study underscores the need for localized, condition-specific guidelines that adapt Surviving Sepsis Campaign principles to obstetric populations, ensuring better adherence and improved maternal and neonatal outcomes. Future efforts should focus on enhancing guideline adherence through targeted education, real-time feedback mechanisms and tailored antimicrobial stewardship strategies.

## Supplementary Material

ofaf337_Supplementary_Data

## Supplementary Data

Supplementary materials are available at *Open Forum Infectious Diseases* online. Consisting of data provided by the authors to benefit the reader, the posted materials are not copyedited and are the sole responsibility of the authors, so questions or comments should be addressed to the corresponding author.

## Notes

***Acknowledgment.*** The Maternal Sepsis Group in addition to named authors: Dr Nicola Cesano, Dr Veronica Bonaldo, Dr Angelo Pan, and Dr Annalisa Abbiati. We acknowledge the effort of all the researchers and coordinators across the various centers who facilitated the data collection process and ensured the integrity and accuracy of the information gathered.

***Financial support***.This research was supported by the EU funding within the Next Generation EU-MUR PNRR Extended Partnership initiative on Emerging Infectious Diseases (Project no. PE00000007, INF-ACT).
