# Supplementary material for: A Multicenter Study in Northern Italy to Evaluate the Impact of a Sepsis Bundle in Obstetric Settings: The SOS Study
Source: Open Forum Infect Dis. 2025 Jun 16;12(7):ofaf337. doi: 10.1093/ofid/ofaf337 (PMC12216898; doi:10.1093/ofid/ofaf337)
Supplement: ofaf337_Supplementary_Data [file ofaf337_supplementary_data.zip › GL_eng_sos_ofid.docx]

**The GENERAL MANAGER of WELFARE of Lombardy region**

**WHEREAS:**

- Maternal sepsis is a life-threatening condition defined as organ dysfunction caused by an infection occurring during pregnancy, childbirth, following an abortion, or in the postnatal period;
- It represents a clinically challenging condition associated with high mortality rates, especially when complicated by septic shock;
- In our country, sepsis is the second leading cause of direct maternal mortality, after obstetric hemorrhage, accounting for approximately 10% of cases;
- In Lombardy, according to an analysis of hospital discharge records (SDO), the incidence of maternal sepsis is 0.33/1000 births/year (analysis conducted on 49,984 births from 2009 to 2013);
- Despite a reduction in maternal deaths linked to infections and sepsis in recent years, maternal sepsis remains one of the leading preventable causes of death;

**REFERENCING** the following regional provisions:

- Directorate General of Health (now Welfare) decree no. 7846 of 29/07/2009, “Prevention and control of hospital and healthcare-associated infections – guidelines for the organization and reporting of activities directed by the Directorate General of Health and accredited healthcare facilities,” which, among other measures, included the establishment of Technical In-depth Study Groups (GAT) on specific topics, including the management of severe sepsis cases;
- Directorate General of Health decree no. 7517 of 5/08/2013, approving the technical document “Integrated strategies to reduce hospital mortality associated with severe sepsis,” outlining pathways and activities for managing interventions in sepsis cases;

**REFERENCING** the Regional Government Resolution (d.g.r.) no. X/7600 of 20/12/2017, “Determinations regarding the management of the socio-health system for the 2018 fiscal year,” which reaffirmed, among other priorities, the importance of risk management projects in the labor/delivery context;

**CONSIDERING** that in obstetric populations, key factors for significantly improving outcomes include the rapid identification and timely management of sepsis, achievable through a clinical-organizational approach to the care pathway;

**ACKNOWLEDGING** that the Directorate General for Welfare relies on the expertise of regional socio-health system professionals based on their clinical, scientific, and professional experience to develop and share guidelines and documents ensuring diagnostic and therapeutic appropriateness, quality, and safety in care pathways;

**NOTING** that a multidisciplinary working group was established within the Directorate General for Welfare, composed of gynecologists, anesthesiologists, obstetricians, and risk managers, with the objective of proposing an operational document, in line with regional Risk Management guidelines, for the early identification and management of sepsis in obstetrics;

**HAVING REVIEWED** the “Operational Guidelines for the Early Identification and Management of Sepsis in Obstetrics” prepared by this multidisciplinary group of experts:

**CONSIDERING** that the document contains recommendations serving as a time-sensitive, multiprofessional, and multidisciplinary reference for clinical practice;

**DETERMINED** to approve the “Operational Guidelines for the Early Identification and Management of Sepsis in Obstetrics” - Annexed and an integral part of this decree;

**DETERMINED** that the recommendations in the document are directed at all healthcare facilities in Lombardy that have Gynecology and Obstetrics services, as well as facilities that, despite not having such services, may encounter pregnancy-related issues;

**DETERMINED** to mandate the ATS (Territorial Healthcare Agencies) to ensure the widest possible dissemination of the approved document to public and private healthcare facilities in their jurisdiction;

**DETERMINED** to publish the document on the Lombardy Region’s portal, [www.regione.lombardia.it](http://www.regione.lombardia.it);

**IN VIEW OF:**

- Regional Law no. 20/2008, as well as the organizational measures of the XI legislature;
- Regional Law no. 23/2015, “Evolution of the Lombardy socio-health system: amendments to Title I and Title II of Regional Law no. 33 of 30 December 2009 (Consolidated Health Law)”;
- The Regional Socio-Health Plan (PSSR) 2010-2014 - approved with resolution no. IX/88 of 17/11/2010, the validity of which was extended by resolution no. X/2989 of 23/12/2014 until the approval of a new plan;

**HEREBY DECREES**:

1. To approve the “Operational Guidelines for the Early Identification and Management of Sepsis in Obstetrics” - Annexed as an integral part of this decree.
2. To establish that the recommendations contained in the document are intended for all healthcare facilities in Lombardy with Gynecology and Obstetrics services and those that, while lacking such services, may face pregnancy-related issues.
3. To mandate the ATS to ensure the widest dissemination of the document referenced in point 1 to public and private healthcare facilities in their jurisdiction.
4. To arrange for the publication of the document on the Lombardy Region’s portal, [www.regione.lombardia.it](http://www.regione.lombardia.it).

**GENERAL DIRECTOR**
Giovanni Daverio
Digitally signed in accordance with current legal provisions.

**Operational Guidelines for the Early Identification and Management of Sepsis in Obstetrics**

Following Regional Decree no. 7517 of 5/8/2013, *“Integrated Strategies to Reduce Hospital Mortality Associated with Severe Sepsis,”* and the *“Recommendation for the Identification, Management, and Treatment of Sepsis in Pediatrics”* issued on 15/1/2014, the “Combating Sepsis in Obstetrics” Working Group of the Lombardy Region has developed, in accordance with Regional Risk Management guidelines, a document aimed at proposing definitions and procedures for the identification and management of sepsis in obstetric populations.

**Specific Objectives:**

1. **Propose clinical-organizational elements** to define a diagnostic-therapeutic care pathway capable of ensuring the application of proven recommendations and strategies to significantly reduce the morbidity and mortality associated with maternal sepsis across all birthing centers.
2. **Recommend actions** for implementing the described strategies.

The practical tools outlined herein (including prevention tools, diagnostic criteria, identification and monitoring tools, therapeutic guidelines, and structural indicators) represent an operational proposal designed to facilitate their implementation and adaptation within individual healthcare facilities.

**TECHNICAL IN-DEPTH WORKING GROUP**

Moscheni Maristella Risk Manager - ASST Nord Milano – Coordinator regional panel discussion
Burato Enrico Risk Manager - ASST Mantova
Comberti Enrico Risk Manager - ASST Spedali Civili Brescia
Cetin Irene Obstetrician Gynaecologist - ASST FBF-Sacco – Coordinator regional project
Antonazzo Patrizio Obstetrician Gynaecologist - ASST FBF-Sacco
Cromi Antonella Obstetrician Gynaecologist - ASST Sette Laghi
Ferrazzi Enrico Obstetrician Gynaecologist - IRCCS CA’ Granda Osp. Maggiore Policlinico
Ghezzi Fabio Obstetrician Gynaecologist - ASST Sette Laghi
Locatelli Anna Obstetrician Gynaecologist - ASST Vimercate
Lojacono Andrea Obstetrician Gynaecologist - ASST Spedali Civili Brescia
Maini Isabella Obstetrician Gynaecologist - ASST Monza
Muggiasca Maria Luisa Obstetrician Gynaecologist - ASST FBF-Sacco
Vergani Patrizia Obstetrician Gynaecologist- ASST Monza
Colciago Elisabetta Obstetrician - Università Milano Bicocca
Del Bo Elsa Obstetrician - IRCCS Polic. San Matteo Pavia
Miglietta Marinella Obstetrician – Osp. "Sacra Famiglia" FBF Erba
Nelli Elisabetta Obstetrician - ASST Lecco
Monti Gianpaola Anaesthesiologist - ASST Grande Ospedale Metropolitano Niguarda - technical and scientific coordinator
D'Andrea Luca Anaesthesiologist - ASST Monza
Moise Gabriella Anaesthesiologist - ASST Nord Milano
Porro Giuliana Anaesthesiologist - IRCCS CA’ Granda Osp. Maggiore Policlinico
Bersani Maurizio Director – Lombardy region
Mozzanica Davide Director – Lombardy region
Picchetti Chiara Secretary – Lombardy region

Consultants:
Dalla Gasperina Daniela - Malattie infettive e Tropicali, ASST Sette Laghi, Varese
Grossi Paolo Antonio - Malattie infettive e Tropicali, ASST Sette Laghi, Varese

**SUMMARY**

[SUMMARY OF RECOMMENDATIONS 7](#_Toc192690086)

[1. Prevention 7](#_Toc192690087)

[2. Maternal Sepsis Alert and Monitoring Systems 7](#_Toc192690088)

[3. New Definitions of Maternal Sepsis and Risk Identification 7](#_Toc192690089)

[4. Initial Diagnostic and Therapeutic Interventions for Maternal Sepsis 9](#_Toc192690090)

[PREFACE 10](#_Toc192690091)

[1. PREVENTION AND RISK FACTORS 11](#_Toc192690092)

[RISK FACTORS 13](#_Toc192690093)

[PREVENTIVE MEASURES 13](#_Toc192690094)

[ANTIBIOTIC PROPHYLAXIS 15](#_Toc192690095)

[2. ALERT SYSTEMS AND MATERNAL SEPSIS: IDENTIFICATION AND MONITORING 16](#_Toc192690096)

[MEOWS CHART: ITS USE AND CLINICAL RESPONSE 17](#_Toc192690097)

[INTENSIFICATION OF CARE 20](#_Toc192690098)

[USE OF MEOWS IN THE IDENTIFICATION AND MONITORING OF THE POTENTIALLY SEPTIC PATIENT 23](#_Toc192690099)

[3. New Definitions of Maternal Sepsis and Risk Identification 24](#_Toc192690100)

[Definitions 24](#_Toc192690101)

[How to identify and diagnose Sepsis/Septic Early 27](#_Toc192690102)

[RISK IDENTIFICATION 31](#_Toc192690103)

[4. First Diagnostic-Therapeutic Interventions for Maternal Sepsis 34](#_Toc192690104)

[BLOOD CULTURES AND OTHER SPECIMENS COLLECTION 36](#_Toc192690105)

[Lactates, Haemoglobin, and Organ Function Parameters 37](#_Toc192690106)

[URINE OUTPUT MONITORING 38](#_Toc192690107)

[OXYGEN THERAPY 38](#_Toc192690108)

[ANTIBIOTIC THERAPY 38](#_Toc192690109)

[SOURCE CONTROL 41](#_Toc192690110)

[VOLEMIC RESUSCITATION 42](#_Toc192690111)

[VENOUS THROMBOEMBOLISM PROPHYLAXIS. 44](#_Toc192690112)

[ATTACHMENTS 45](#_Toc192690113)

[ANNEX 1. PARAMETERS OF THE MEOWS SHEETS 45](#_Toc192690114)

[ANNEX 2. INTENSIFICATION OF MONITORING 50](#_Toc192690115)

[ANNEX 3. IMPLEMENTATION AND OBSTACLES TO THE USE OF THE MEOWS CARD 51](#_Toc192690116)

[ANNEX 4. Choice of antibiotic regimen, remodulation and duration of treatment 52](#_Toc192690117)

[ANNEX 5. Organisational Check-list 56](#_Toc192690118)

[BIBLIOGRAPHY 63](#_Toc192690119)

# SUMMARY OF RECOMMENDATIONS

## 1. Prevention

- Implement preventive measures against infection/sepsis in the obstetric population based on solid evidence.
  Primary strategies include identifying risk factors, employing evidence-based clinical practices to control infections (e.g., appropriate hygiene and disinfection methods, antibiotic prophylaxis protocols), and promoting health education among the obstetric population.
- Use monitoring and alert systems such as the *Modified Early Obstetric Warning Score (MEOWS)* for the early detection of infection/sepsis.
- Clearly document and highlight risk factors in medical records for every hospital visit or during inpatient care.
- Train healthcare staff on risk factors and preventive measures against infection/sepsis in obstetrics.

## 2. Maternal Sepsis Alert and Monitoring Systems

- Monitoring Vital Signs: For all pregnant women or those within 42 days postpartum, arriving at the birth centre (Obstetric ER or General ER) or already hospitalized, use MEOWS for vital sign monitoring.
- During labour, vital signs should be recorded in the partograph following local protocols.
- Post-delivery or post-operative patients must have their final set of vital signs documented on the MEOWS chart before transfer to the ward.
- Frequency of MEOWS monitoring depends on risk levels, admission diagnosis, clinical complications, and MEOWS scores.

**Rapid Clinical Deterioration**:

- If one yellow MEOWS parameter is detected, evaluate for infection/sepsis/shock by assessing symptoms/signs and organ damage.
- Escalate care if there is rapid clinical deterioration:
  - Initiate the "intensified care" pathway.
  - If immediate evaluation by an on-call physician is not possible, involve a senior obstetrician or anaesthesiologist or escalate to the Clinical Director.
  - Consider transferring the patient to a higher-level facility if adequate care cannot be guaranteed locally.

**Operational Responses**:

- Perform the *ABCDE sequence* (Airway, Breathing, Circulation, Delivery, Execution of care plan).
- Ensure continuous clinical reassessment, monitor the patient’s condition, and engage multidisciplinary expertise (e.g., obstetrician, infectious disease specialist, anesthesiologist).

## 3. New Definitions of Maternal Sepsis and Risk Identification

**Definitions**:

- **Maternal Sepsis**: Diagnosed during pregnancy, delivery, post-abortion, or postpartum when there is confirmed or suspected infection alongside one or more criteria for organ dysfunction:
  - Oxygen need to maintain SpO₂ > 95% or PaO₂/FiO₂ < 400.
  - Platelet count < 100 x 10⁶/L.
  - Bilirubin level > 1.2 mg/dL.
  - Systolic BP < 90 mmHg or MAP < 75 mmHg.
  - Altered consciousness (responsive to verbal/painful stimuli or unresponsive).
  - Creatinine level > 1.2 mg/dL.

**Organ Dysfunction**: Defined by a modified SOFA (Sequential Organ Failure Assessment) score ≥ 1.

**Septic Shock**: Suspected or confirmed infection with hypotension requiring vasopressors to maintain MAP ≥ 65 mmHg and serum lactate > 2 mmol/L after adequate fluid resuscitation.

**Diagnosis**

- **Infection Detection**: Conduct a thorough history and physical examination for signs/symptoms of infection.
- **Organ Dysfunction Assessment**: Evaluate vital signs (MEOWS) and perform laboratory tests (sepsis panel).
- **Clinical Practice**: For patients with presumed or confirmed infections, always assess for organ dysfunction, and vice versa, for organ dysfunction without clear cause, investigate for potential infections.

**Operational Procedures**

**Step 1**: For suspected or confirmed infections:

- Evaluate by obstetrician and on-call physician.
- Monitor vital signs using MEOWS.
- Assess fetal well-being.

**Step 2**: Tailor actions based on MEOWS alert codes:

- **Low Risk**: Suspected/confirmed infection with 1 yellow MEOWS parameter. Monitor vitals per MEOWS, assess fetal well-being, and conduct relevant diagnostic tests if required.
- **Intermediate Risk**: Suspected/confirmed infection with 2 yellow or 1 red MEOWS parameters. Conduct organ dysfunction evaluation (modified SOFA score), serum lactate measurement, Sepsis Six protocol, and initiate vital support if needed.
- **High Risk**: Suspected/confirmed infection with >2 yellow or >1 red MEOWS parameters, or organ dysfunction (modified SOFA score ≥1), or serum lactate > 2 mmol/L. Requires joint evaluation by an anesthesiologist, obstetrician, and infectious disease specialist (if available).

**Laboratory Tests**: A "sepsis panel" includes complete blood count, lactate, electrolytes, kidney and liver function tests, coagulation markers (PT-PTT), and inflammation markers (CRP or PCT).

## 4. Initial Diagnostic and Therapeutic Interventions for Maternal Sepsis

Implement all the "Sepsis Six" interventions in every case of suspected or confirmed maternal sepsis or septic shock within the first hour of diagnostic suspicion.

The "Sepsis Six" framework includes six interventions, divided into:

- **3 diagnostic actions**: blood culture collection, lactate measurement and other laboratory tests, monitoring of urine output.
- **3 therapeutic actions**: oxygen administration, fluid resuscitation, and antibiotic therapy.

Specifically, apply the "Sepsis Six" protocol for all patients with:

- **Intermediate risk**: signs/symptoms of infection + 2 yellow or 1 red MEOWS (Modified Early Obstetric Warning System) indicators.
- **High risk**: signs/symptoms of infection + >2 yellow or >1 red MEOWS indicators or mSOFA ≥ 1.
- For **low-risk patients**, the implementation of "Sepsis Six" should be based solely on medical indication.
- Before administering antibiotics, perform at least two sets of blood cultures. If antibiotic therapy is already underway, collect samples prior to the next dose.
- Conduct cultures from all anatomical sites potentially serving as the source of infection.
- Appropriate culture tests should be available in all inpatient units and emergency departments.
- Measure blood lactate levels in all obstetric patients presenting with signs or symptoms of suspected or presumed infection. This can be done via arterial or venous sampling through peripheral or central access using point-of-care testing methods.
- Evaluate organ functionality parameters ("sepsis panel").
- In cases of sepsis or septic shock, red blood cell transfusion (in the absence of other conditions like myocardial ischemia, severe hypoxemia, or massive hemorrhage) is recommended when hemoglobin levels are <7 g/dL.
- Monitor urine output in all obstetric patients with suspected or confirmed infection/sepsis/septic shock.
- Assess and monitor respiratory function (respiratory rate and SpO2) in all patients with suspected or confirmed sepsis.
- Administer oxygen therapy with 100% FiO2 using a non-rebreathing reservoir mask in obstetric patients with suspected or confirmed sepsis.
- Administer intravenous antibiotic treatment as soon as possible and within the first hour in all cases of suspected or confirmed sepsis/septic shock, especially for patients at intermediate and high risk.
- Start empirical treatment with one or more broad-spectrum antibiotics targeting the most likely pathogens causing sepsis or septic shock.
- In obstetrics, the primary septic source is the genital tract, followed by the urinary tract and wounds. Studies indicate that the most commonly isolated microorganisms in obstetrics are *E. coli*, Group B Streptococcus, and anaerobic bacteria. Mixed infections (Gram-positive and Gram-negative) are frequently observed.
- The combination of penicillin/beta-lactamase inhibitor with an aminoglycoside can be considered a first-line treatment for maternal sepsis.
- Once the pathogen is identified and antibiotic sensitivity is obtained, or clinical improvement is documented, adjust the antibiotic treatment accordingly.
- Identify the septic focus and, where possible, eliminate it.
- If a septic focus is eradicated, collect samples of the infected biological material (biological fluids or tissue biopsies) and send them for culture testing.
- Always consider possible non-obstetric septic foci (e.g., appendicitis, pancreatic abscess, intestinal infarction, influenza).
- Promptly remove vascular access devices deemed potential septic foci once alternative access has been established.
- Assess fetal well-being in all cases of hypoperfusion or established shock. Evaluate the decision to deliver the fetus in cases of obstetric sepsis, balancing the risks associated with gestational age, maternal condition, and fetal status. Maternal stabilization takes precedence before delivery in cases of compromised fetal well-being, as maternal intervention may improve fetal condition.
- In hypotensive patients with suspected or confirmed sepsis/septic shock, administer a fluid bolus of 30 mL/kg of crystalloids in increments of 500 mL within the first hour. After the 20th week of pregnancy, position the patient in the left lateral decubitus position.
- Perform fluid resuscitation using crystalloids, alternating normal saline with balanced electrolyte solutions. Albumin supplementation may be considered for patients requiring high fluid volumes.
- Evaluate the response to fluid resuscitation in terms of effectiveness (e.g., improved blood pressure, peripheral perfusion: urine output and lactate levels) and complications.
- Continue fluid resuscitation until reaching the target mean arterial pressure (MAP) of ≥65 mmHg, with a maximum dose of 30 mL/kg. Additional fluids should only be prescribed by an anesthesiologist or intensivist.
- Pharmacological prophylaxis for venous thromboembolism is recommended in cases of maternal sepsis.

# PREFACE

Maternal sepsis is a life-threatening condition defined as organ dysfunction caused by an infection occurring during pregnancy, childbirth, after an abortion, or in the postnatal period (World Health Organization, Statement on Maternal Sepsis, 2017). It represents a challenging clinical condition and remains associated with high mortality, particularly when complicated by septic shock.

Immunological competence during pregnancy does not decrease but undergoes modifications that allow the maternal body to adapt to the fetus. This creates a state of immune modulation rather than immunosuppression. For example, the reduction in IgG production is compensated by an increase in leukocytes. The reduction in the number and function of CD4+, CD8+, and natural killer cells may impact antiviral, antifungal, or antiparasitic responses and delay pathogen clearance. However, increased innate immunity during pregnancy may help prevent infections and explain the absence of greater susceptibility to pathogens. Indeed, evidence for increased susceptibility to infections during pregnancy is relatively weak (Kourtis, 2014).

Although pregnant women do not seem more prone to infections in general, physiological changes and immunological alterations associated with advancing pregnancy can increase the severity of diseases caused by certain pathogens (Kourtis et al., 2014).

Estimates of the incidence of sepsis during pregnancy are primarily based on retrospective studies and vary significantly depending on the detection methods and definitions used. According to the literature, the incidence of sepsis during pregnancy ranges from 0.1 to 0.4 per 1,000 births annually (Van Dillen, 2013; Italian Obstetric Surveillance System, 2014-2015). In the Lombardy region, analysis of hospital discharge records (SDO) indicated a maternal sepsis incidence of 0.33 per 1,000 births annually (based on 49,984 births from 2009 to 2013).

Mortality rates for maternal sepsis range from 0.6 to 1.1 per 100,000 births, according to Australian and American data. Sepsis during pregnancy accounts for 10.7% of maternal deaths (2003-2012), as reported by the World Health Organization. Similarly, in Italy, maternal sepsis is the second leading cause of direct maternal death after obstetric haemorrhage, accounting for 10% of cases according to the pilot project on maternal mortality surveillance conducted by the National Institute of Health.

Recent studies show that maternal sepsis has a mortality rate ranging from 1.8% to 17.6%, increasing to 28-33% in cases of septic shock. Despite reductions in maternal deaths due to infections and sepsis in recent years, maternal sepsis remains one of the leading preventable causes of death.

In the obstetric population, as emphasized by the Surviving Sepsis Campaign (SSC 2016) guidelines for adults, key factors that can significantly improve prognosis include the rapid recognition and timely management of sepsis. This can be achieved through the systematic use of assessment procedures, scheduled reassessments, and the establishment of alert thresholds that trigger response actions based on decision-making algorithms—in summary, through a "clinical-organizational" approach to patient care pathways.

To develop an operational document for all regional facilities aimed at the early identification and management of sepsis in obstetrics, the Lombardy Region established a multidisciplinary working group consisting of gynaecologists, anaesthesiologists, midwives, and risk managers. The recommendations arising from this group serve as a clinical reference within a time-dependent, multi-professional, and multidisciplinary framework and should not be interpreted as rigidly absolute guidelines.

The document includes the following chapters:

1. Prevention and Risk Factors.
2. Alert Systems and Maternal Sepsis: Identification and Monitoring.
3. New Definitions of Maternal Sepsis and Risk Identification.
4. Initial Diagnostic and Therapeutic Interventions for Maternal Sepsis.

# 1. PREVENTION AND RISK FACTORS

This chapter examines interventions aimed at reducing the risk of infections/sepsis during pregnancy, childbirth, and the postnatal period.

**Rationale**

Prevention refers to the set of "evidence-based" strategies and behaviours designed to reduce the risk factors for infections/sepsis in the obstetric population. Primary prevention plays a fundamental role for everyone.

Prevention is achievable through appropriate infection control programs, which include:

- Strict monitoring of infection patterns.
- Training healthcare staff on infection control practices, with particular attention to risk factors.
- Utilizing appropriate methods and devices for cleaning, hygiene, and disinfection.
- Implementing preventive pre-surgical/pre-delivery screenings.
- Providing widespread health education to the obstetric population.

In May 2017, the World Health Organization (WHO) approved a resolution aimed not only at improving the diagnosis and treatment of sepsis but also at prioritizing prevention, with particular attention to infection control and limiting the spread of antibiotic resistance.

It is well established that preventive measures, with a focus on risk factors, effectively reduce the incidence of infections/sepsis in the obstetric population, thereby improving associated morbidity and mortality.

It is crucial to always consider the physiological changes during pregnancy and their impact on diagnosis and clinical management (see Table 1).

#### Table 1: Physiological Changes During Pregnancy and Their Impact on Diagnosis and Management

(Cordioli, 2013)

| **SYSTEM** | **PHYSIOLOGICAL CHANGES** | **IMPACT ON DIAGNOSIS/MANAGEMENT** |
| --- | --- | --- |
| Cardiovascular | ↓ Peripheral vascular resistance  ↓ Blood pressure  ↑ Heart rate  ↑ Cardiac output | Masking of early signs/symptoms of sepsis |
| Blood | \|  \| \| --- \|  \| ↑ Plasma volume  ↑ Red blood cell volume \| \| --- \| | Anaemia  Delayed signs of hypovolemia  Significant reduction in oxygen delivery to tissues |
| Respiratory | ↑ Tidal volume  ↓ Residual volume  ↑ Minute ventilation by 30-40%  ↑ Respiratory rate  ↓ PaCO2 | Delayed compensation for metabolic alkalosis  Impaired oxygenation |
| Renal | \| Ureteropelvic dilation  ↓ Ureteral pressure due to smooth muscle relaxation  Bladder flaccidity  ↑ Intravesical pressure from uterine compression  ↑ Vesicoureteral reflux  ↑ Renal plasma flow  ↑ Glomerular filtration rate  ↓ Blood urea nitrogen and creatinine levels \| \| --- \|  \|  \| \| --- \| | Delayed identification of renal damage due to sepsis  Increased predisposition to urinary tract infections |
| Gastrointestinal | ↓ Intestinal muscle tone  Delayed gastric emptying  Diaphragm elevation  Altered bile composition  ↑ Proinflammatory cytokine production by Kupffer cells | ↑ Risk of bacterial translocation  ↑ Risk of aspiration pneumonia  ↑ Risk of cholestasis, hyperbilirubinemia, jaundice |
| Coagulation | ↑ Factors VII, VIII, IX, X, XII, Von Willebrand factor, and fibrinogen  ↓ Protein S  ↓ Fibrinolytic activity | ↑ Risk of thrombotic events  ↑ Risk of disseminated intravascular coagulation (DIC) |
| Genital | ↓ Vaginal pH  ↑ Glycogen in vaginal epithelium | ↑ Risk of chorioamnionitis |

## RISK FACTORS

The assessment and management of risk factors represent the primary tool for preventing infections/sepsis in the obstetric population.
Risk factors are related to constitutional factors and the clinical history of the obstetric patient and vary depending on the trimester of pregnancy (Table 2).

## PREVENTIVE MEASURES

The main preventive measures for infections/sepsis in the obstetric population include:

- Proper use of clinical practices aimed at infection control (e.g., appropriate methods and devices for cleaning, hygiene, and disinfection, antibiotic prophylaxis protocols, etc.).
- Use of monitoring and alert systems (Modified Early Obstetric Warning Score, MEOWS) to enable early identification of infection/sepsis (see Chapter 2).
- Health education programs for hospital staff and the obstetric population to increase awareness of the issue.
- Use of logistical-organizational checklists and implementation of clinical audits to promote preventive measures and early diagnosis.
- Monitoring the phenomenon.

**HOSPITAL PREVENTION MEASURES**

- Proper handwashing (WHO project; training).
- Use of alcohol-based hand gel by healthcare workers and visitors.
- Avoid shaving pubic and perineal hair.
- Avoid vaginal examinations in pregnant women with premature rupture of membranes unless risk factors are present.
- Avoid frequent vaginal examinations during low-risk labor.
- Allow sufficient time for the third stage of labor without unnecessary invasive maneuvers.
- Avoid unnecessary cesarean sections.
- Maintain strict asepsis in the operating room.
- Adhere to hospital protocols for bladder catheterization.
- Follow hospital protocols for managing peripheral and central venous access.
- Avoid unjustified use of antibiotics (adhere to hospital protocols) to minimize the risk of antibiotic resistance.
- Advise patients upon discharge to promptly identify and report potential symptoms of infection: chills, fever, malaise, hypotension, vomiting, abdominal pain, or abnormal vaginal discharge.

**Table 2. Risk Factors for Infections/Sepsis in the Obstetric Population**

| **TIMEFRAME** | **RISK FACTORS** |
| --- | --- |
| **Antepartum** |  |
| Constitutional and Lifestyle | Age > 40 or < 18 years  BMI > 30  Cigarette smoking  Low socioeconomic status  Ethnic minority |
| Clinical History | Diabetes  Anaemia  Immune system disorders  Use of immunosuppressive drugs  Close contact with individuals/family members with bacterial/viral infections |
| Pregnancy-related | Amniocentesis or other invasive procedures  Cervical cerclage  Prolonged rupture of membranes  Pathological vaginal discharge |
| **Intrapartum** |  |
| Labor and Delivery | Chorioamnionitis  Repeated vaginal examinations during labour  Operative vaginal delivery  Manual removal of the placenta  Caesarean delivery  Early haemorrhage  Perineal trauma or hematomas |
| **Postnatal period** |  |
| Postpartum | Retained placental tissue  Urinary tract infections  Surgical wound/episiotomy site infection  Late haemorrhage  Epidural catheter site infection  Mastitis |

**PREVENTION DURING PREGNANCY**

- Nutritional education:
  - Avoid foods that may transmit severe infections such as listeriosis, salmonellosis, and toxoplasmosis (refer to Ministry of Health guidelines on pregnancy nutrition).
  - Proper diet to prevent excessive weight gain and the onset of metabolic syndrome.
- Personal hygiene and frequent handwashing.
- Avoid crowded environments.
- Avoid direct and prolonged contact with children and adults with upper respiratory tract infections (particularly for women working with young children due to the risk of Group A Streptococcus infection).
- Advise against travel to high-risk infectious areas.
- Recommend flu vaccination at any stage of pregnancy (the Ministry of Health advises vaccination during the second and third trimesters).
- Prevent and treat anemia (refer to ISS postpartum hemorrhage guidelines).
- Identify pregnant women with low socioeconomic status or from ethnic minorities during the first obstetric visit and offer them additional support (e.g., extra obstetric counseling, social support, and cultural mediation services) (NICE Guideline 2010, updated 2018).

## ANTIBIOTIC PROPHYLAXIS

Antibacterial prophylaxis aims to prevent infections in the presence of risk factors or during invasive procedures caused by pathogens from the endogenous flora. Antibiotics are typically administered before potential exposure to achieve therapeutic tissue concentrations at the time of the procedure and for a limited duration (<24 hours). The selected antibiotic should be long-acting, narrow-spectrum, cost-effective, and have minimal side effects. Table 3 summarizes the main indications.

**Table 3.** Indications and Non-Indications for Antibiotic Prophylaxis in Obstetric Care (WHO Recommendations for prevention and treatment of maternal peripartum infections, 2015).

| **RECOMMENDED** | **NOT RECOMMENDED** |
| --- | --- |
| In labor: with a positive swab or risk factors for Group B Streptococcus (GBS) infection | Outside labor: even if positive swab for GBS infection |
| Prolonged rupture of membranes | Threatened preterm labor with intact membranes |
| Elective and/or urgent cesarean delivery | Meconium-stained amniotic fluid |
| Manual removal of the placenta |  |
| Third- and fourth-degree perineal tears |  |

**Recommendations**

- Implement infection/sepsis prevention measures in the obstetric population whose effectiveness is proven by strong evidence.

- Use monitoring and warning systems (MEOWS) as a tool for early detection of infection/sepsis in the obstetric population.

- Recognise and report risk factors in the health record in an unobtrusive way at each hospital admission and/or during the hospitalisation of the obstetric patient.

- Plan training of health personnel on risk factors and infection/sepsis prevention measures in the obstetric population.

**Actions**

- Prepare protocols on recommended and specific infection/sepsis prevention measures for the obstetric population.

- Plan training of health personnel on risk factors and infection/sepsis prevention measures in the obstetric population.

# 2. ALERT SYSTEMS AND MATERNAL SEPSIS: IDENTIFICATION AND MONITORING

**Rationale**
Pregnancy, childbirth, and the postpartum period are physiological events; clinical monitoring throughout the childbirth process must always be an integral part of the care process to early recognize any deterioration in the general condition and prevent adverse outcomes. A clear example is sepsis, where the physiological changes of pregnancy can mask its signs and symptoms, leading to a delayed diagnosis until there is significant clinical deterioration (Table 1).
“Women with serious illness, especially sepsis, may appear deceptively well before suddenly collapsing, often with little or no warning;” (Centre for Maternal and Child Enquiries 2011).
Supporting this, the most recent maternal mortality data indicate sepsis as the second leading cause of direct maternal death, highlighting the need to define dedicated pathways for risk identification and appropriate responses to the most frequent and often fatal acute conditions.

Early warning systems ("Early Warning Score", EWS) were developed in the 1990s based on evidence that even minimal changes in physiological parameters precede clinical deterioration and that recognizing these changes with an appropriate response can improve outcomes for acutely ill patients. The EWS adapted to the obstetric population (“Modified Early Obstetric Warning Score”, MEOWS) have been introduced more recently in maternity units to facilitate risk recognition and to outline a therapeutic-care pathway based on clinical severity for women who are developing or have developed a critical illness.
In fact, the signs of impending shock are often not recognized early in this population, and the response is frequently disorganized within a very complex clinical picture.
EWS/MEOWS rely on the measurement of vital parameters on a scoring or color-coded scale that allows for quick and shared assessment of clinical status with the aim of providing a reproducible measure of whether and to what extent a patient is at risk of deterioration.
This alert system also allows the identification (based on score/color code) of the type of response to be activated in terms of timing (frequency of measurement/monitoring) and modality (alert level).
Such procedures promote a standardized approach to maternal well-being, with particular attention to intensification of care (when necessary) and facilitate teamwork and communication among healthcare providers.
The implementation of the MEOWS alert system (via paper or electronic chart) follows the recommendations from the 2007 and 2011 maternal mortality reports (Confidential Maternal Enquiry Report, McClure et al., 2011) and the 2016 guidelines from the Italian National Institute of Health (Agenas: Clinical-Organizational Guidelines for the Prevention of Pregnancy-Related Complications) for the assessment, monitoring, and management of obstetric patients.
MEOWS indeed has high sensitivity (89%) in predicting morbidity and a reasonable specificity (79%) supporting its use in the obstetric population.
The MEWS commonly used for the adult population have proven to be an accurate tool in identifying patients with infections at risk of clinical deterioration, underlining their key role as a screening tool in time-dependent conditions like sepsis. Early recognition and timely treatment of sepsis represent key interventions in improving morbidity and mortality in the obstetric population (as in the adult population).

The objective of this chapter is to facilitate the identification and management of potentially septic obstetric patients through the use of a standardized early warning system (MEOWS).
This document provides, in addition to the MEOWS tool, guidelines on the frequency of clinical monitoring, the urgency of intervention, and the competencies required at different severity levels, with particular emphasis on sepsis cases. It also highlights the importance of having an organized, defined, and shared response in all healthcare facilities with a maternity ward to enable early recognition and timely intervention for potentially septic patients.

## MEOWS CHART: ITS USE AND CLINICAL RESPONSE

The Regional Working Group proposes the use of the MEOWS alert system as outlined in the 2014 Irish National Guidelines. The proposed MEOWS is based on the measurement of seven physiological parameters; their clinical significance and how to record them are provided in **Annex 2**.
Each parameter is graded in levels, with each level assigned a color code. The sum of the color codes provides the degree of deviation from normal physiology and determines the type of response to be implemented.
To facilitate its use and promote standardization of the tool, a color-coded MEOWS chart is proposed, allowing the immediate identification of deterioration alarms (Figure 1).

The MEOWS system, with its corresponding chart, includes:

- Measurement of vital parameters according to a frequency determined by the risk level, the admission diagnosis/complication, and the last measurement (Table 6);
- An alert level based on the color code score, which modulates the urgency of the clinical response and the level of professional competence required for the interventions (Figures 2 and 3);
- The MEOWS score provides three levels of alert corresponding to: 1 YELLOW PARAMETER, 2 YELLOW PARAMETERS, or 1 RED PARAMETER, >2 YELLOW PARAMETERS or >1 RED PARAMETER.

MEOWS must be used systematically to provide standardized measures of severity levels in acute conditions and as a surveillance tool to track clinical progression and to alert about any potential deterioration.

**Figure 1.** Modified Early Obstetric Warning Score (MEOWS).


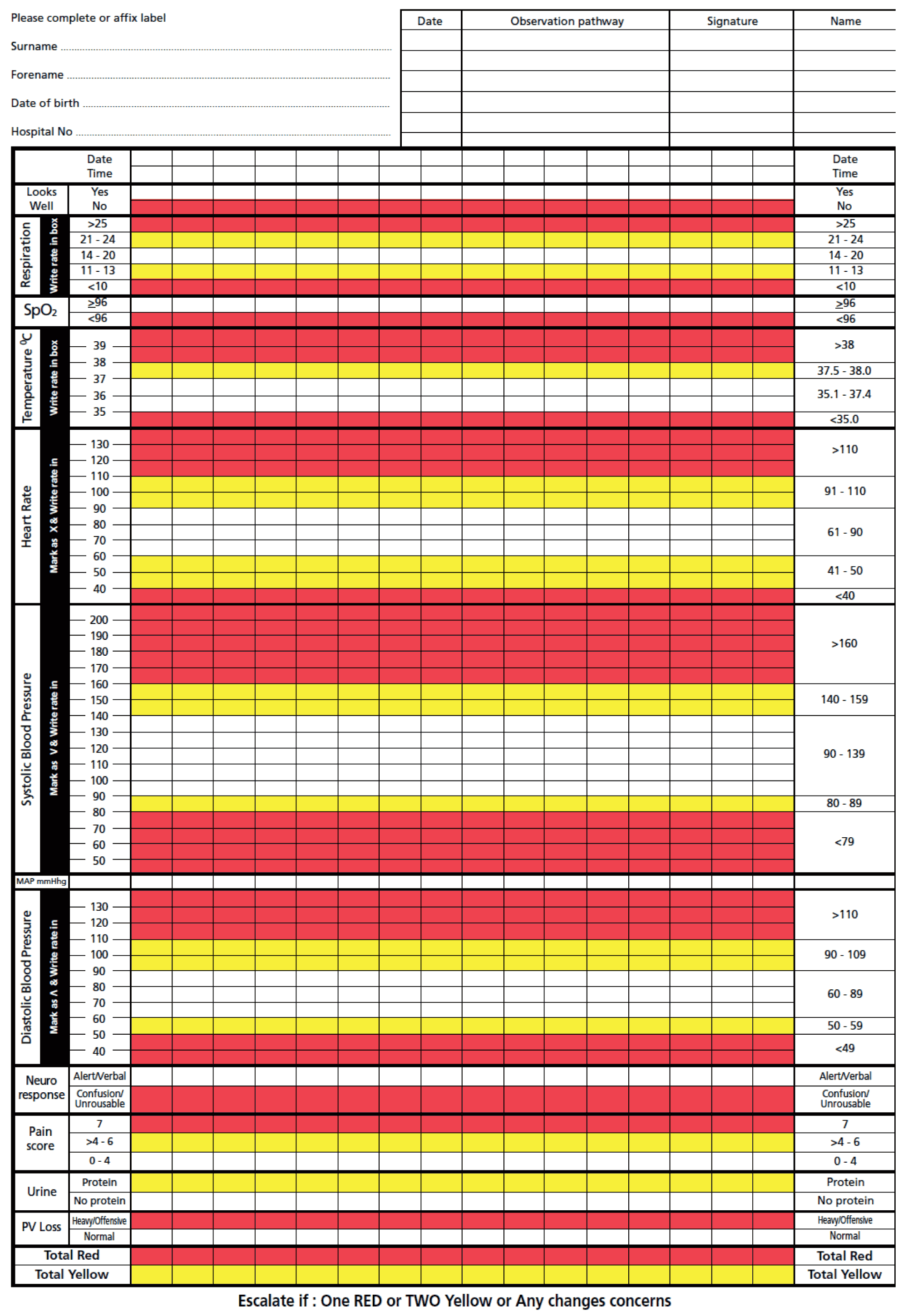
 Early

**Table 4.** Frequency of MEOWS Parameter Measurement

| **Clinical Situation** | **Minimum Frequency of MEOWS Parameter Measurement** |
| --- | --- |
| Low-risk pregnant woman with uncomplicated pregnancy (hospitalized) | Complete assessment upon admission, then checks every 12 hours, adjustable based on clinical indication |
| Postpartum low-risk woman with uncomplicated pregnancy and delivery | Complete assessment of all parameters after delivery. Then, MEOWS parameters checked every 12 hours until discharge unless clinically indicated otherwise |
| Antenatal or Postnatal | Minimum Frequency of MEOWS Parameter Measurement |
| Hypertensive disorders | Daily monitoring of all parameters including urinalysis, with MEOWS parameters evaluated every 4 hours |
| Suspected or confirmed maternal infection | Daily monitoring of all parameters with MEOWS parameters evaluated every 4 hours |
| Any other clinical concern | Complete assessment of all vital signs recorded at least every 12 hours, followed by additional assessments based on overall clinical picture |
| Emergency Situation | Based on clinical evaluation |
| Blood transfusion | Follow local protocols |
| After caesarean section or surgery during pregnancy/postpartum* | A complete assessment of vital signs (urinalysis not applicable) should be recorded: — every 15 minutes in recovery room, — every 30 minutes for 2 hours postpartum, — then every 4-8 hours for 48 hours, — once daily until discharge.  *The frequency of parameter measurements should follow the MEOWS protocol |

**Recommendations**

— In all pregnant women or within 42 days of delivery arriving at the maternity unit (Obstetric or General Emergency Department) or already hospitalized, vital parameter monitoring should be conducted using a specific obstetric early warning score (MEOWS).

— For women in labor, from the moment of admission to the delivery room, vital parameter measurements following the local protocol should be documented in the partogram.

— Upon leaving the delivery room or operating room, the last vital parameter measurements should be recorded on the MEOWS sheet before transferring the patient to the ward.

— The frequency of MEOWS parameter measurement is determined by the evolving risk, admission diagnosis and/or clinical complications, and the last evaluation (color code score); the level of alert on the MEOWS is determined solely by the evaluation (color code score).

Figure 2. Diagram for the application path of MEOWS parameter measurement *[Translator’s note: the text in the image is in Italian, but its content is already explained in the paragraph*]

**Actions**

— Implement a training program on the systematic assessment and standardized alert system for the obstetric population (MEOWS) and its use in clinical practice for all healthcare professionals involved in the care of pregnant or postpartum patients.

## INTENSIFICATION OF CARE

In case of deviation from normal physiology, the MEOWS color code score provides three levels of alert that modulate the urgency of clinical response and the level of professional competence required for assistance. The detection of Yellow or Red vital parameters requires increased frequency of monitoring and/or the intervention of an Obstetrician and Anesthetist. Alert levels modulate the urgency of clinical response and the required level of expertise.

Figure 3 MEOWS flowchart *[Translator’s note: the text in the image is in Italian, but its content is already explained in the paragraph]*

**Recommendations**

— If ≥1 yellow MEOWS parameter is detected, consider and rule out the suspicion of infection/sepsis/septic shock by looking for signs and symptoms of infection and organ damage that could confirm or exclude the diagnosis of sepsis/septic shock.

— In case of rapid clinical deterioration, a "care intensification pathway" should be implemented, which may require the involvement of more experienced personnel or a multidisciplinary consultation for assessment, evaluation, and management of the patient (Annex 2).

— If rapid clinical deterioration occurs and immediate evaluation by the on-call physician is not possible, consider involving another Obstetrician-Gynecologist and/or more experienced Anesthetist, or early engagement with the Clinical Director/Head of Department. The inability for immediate evaluation by the on-call physician is a clear indication to call the on-call physician. — If rapid clinical deterioration occurs and it is not possible to transfer the patient to a more suitable facility, DO NOT delay necessary diagnostic-therapeutic interventions (e.g., laboratory tests, ECG, ABG, chest X-ray, etc.).

— In case of rapid clinical deterioration, the ABCDE assessment sequence (Figure 4) should be followed: Airway, Breathing, Circulation, Delivery, and Execution of the care pathway. The critical care pathway for the pregnant patient is illustrated in Figure 5.

— In case of clinical deterioration, continuous reassessment of the patient's condition should be conducted, including the ABCDE sequence, physical examination, review of clinical documentation, and medical history. — In case of clinical deterioration, while waiting for the physician’s assessment, the Obstetrician should intensify monitoring and patient care according to the protocol shown in Table 4.

— In case of clinical deterioration, multidisciplinary expertise should be involved early (Obstetrician, Infectious Disease Specialist, Anaesthesiologist).

Figure 4 ABCDE sequence for the assessment of the patient with signs/symptoms of clinical deterioration. *[Translator’s note: the text in the image is in Italian, but its content is already explained in the paragraph*]

Figure 5 Critical pregnancy care pathway *[Translator’s note: modified English version of the image included in the original Operational Guidance Doc]*****

## USE OF MEOWS IN THE IDENTIFICATION AND MONITORING OF THE POTENTIALLY SEPTIC PATIENT

The use of EWS in identifying acute phases of diseases and preparing an appropriate care plan highlights their role in time-dependent conditions like sepsis. Early diagnosis and prompt treatment of sepsis are key interventions that can improve morbidity and mortality in obstetric patients, as in adult patients. Specifically, MEOWS can play a key role in the management pathway of maternal sepsis by:

1. **Identification of patients with infections/sepsis**: Alterations in vital parameters detected by the MEOWS alert system are often the first signs of infection/sepsis, such as changes in consciousness, tachycardia, or fever, detectable by one or more yellow and/or red color codes. If ≥1 yellow MEOWS parameter is found, always consider the suspicion of infection/sepsis/septic shock by looking for signs or symptoms of an infection and organ damage not otherwise explained (Figure 1); the combination of suspected or confirmed infection + organ damage confirms the diagnosis of sepsis.
2. **Identification of possible organ damage in the patient with suspected or confirmed infection**: In a patient with signs or symptoms of infection, some MEOWS parameters, when altered (yellow or red parameters), may indicate organ damage, such as hypotension, increased respiratory rate, reduced SpO2, or altered consciousness. Therefore, in any evaluation of an obstetric patient, particularly with suspected infection, it is essential to assess vital parameters using the MEOWS alert system.
3. **Identification of the risk of deterioration in patients with infections/sepsis**: As proposed by the regional working group, in pregnant or postpartum women arriving at the maternity unit or already hospitalized, with an infection, the MEOWS alert system can stratify the evolving risk and define the appropriate diagnostic-therapeutic care pathway, as indicated in Chapter 3:
   - **Intermediate Risk** (signs/symptoms of infection + 2 yellow or 1 red MEOWS): Always requires the evaluation of the modified SOFA score, serum lactate measurement, and the Sepsis Six protocol.
   - **High Risk** (signs/symptoms of infection + >2 yellow or >1 red MEOWS or modified SOFA score ≥1 or lactate >2 mmol/l): Always requires the intervention of an Anesthetist and the execution of the Sepsis Six protocol.

**Recommendations**

— In case of ≥1 yellow MEOWS code, always consider the suspicion of infection/sepsis/septic shock by looking for signs or symptoms of infection and organ damage that cannot be explained otherwise.
— At every obstetric patient evaluation, particularly in suspected infection, it is crucial to assess vital parameters using the MEOWS alert system with the aim of detecting potential organ damage. — In an obstetric patient with signs or symptoms of infection, always complete the assessment of vital parameters using MEOWS; this approach suggests risk stratification and the definition of the corresponding diagnostic-therapeutic care pathway.
— In any woman suspected of sepsis, immediately begin the care intensification pathway; transfer to an intensive care unit should be evaluated by the Anesthetist.

**Actions**

— Implement standard operating procedures for the use of MEOWS in obstetric patients with suspected infection/sepsis/septic shock.

**Rationale**

Inadequate documentation (e.g., lack of monitoring instructions, imprecise therapeutic prescriptions, illegible handwriting, unconventional abbreviations) can lead to adverse events in the management of critically ill patients.

**Recommendations**

— Constantly document all vital parameters and clinical responses from the MEOWS alert system, noting the time and the operator.

**Actions**

— Implement the use of a standardized MEOWS sheet as the sole registration form for vital parameters. — Implement monitoring systems to ensure compliance with MEOWS usage.

# 3. New Definitions of Maternal Sepsis and Risk Identification

#### Rationale

Literature data consistently highlight that early identification of septic shock/sepsis in obstetric populations, followed by prompt therapeutic intervention, has a significant impact on survival rates. Reducing mortality requires the establishment of shared protocols for the early recognition of infections and organ dysfunction related to sepsis outside of critical care areas. This is particularly important in a population where clinical progression is faster and often more fatal. Maternal sepsis should therefore be defined as an obstetric emergency.

This document aims to provide a practical approach to assessing patients with infection/sepsis/septic shock in primary care settings (emergency departments, delivery rooms, or wards), by gynecologists and midwives. It includes practical tools to evaluate risk factors, along with a detailed and structured list of potential signs and symptoms of clinical relevance. It is believed that using a simple and standardized assessment tool could help healthcare professionals identify the more severely ill patients, who may require life-saving treatments.

## Definitions

**Definition of Sepsis for Pregnant and Postpartum Women**
In 2016, the World Health Organization (WHO) proposed a new definition for maternal sepsis:

"Maternal sepsis is a life-threatening condition defined as organ dysfunction resulting from an infection acquired during pregnancy, childbirth, the post-abortion period, or the postpartum period (i.e., the time from membrane rupture or delivery to the 42nd day of the puerperium)."

This definition was developed based on a systematic review of existing definitions (including the Third International Consensus Definitions for Sepsis and Septic Shock 2016 for the adult population) and through international technical consultation. The new WHO proposal aligns with the recent adult sepsis definition, considering sepsis as a dysregulated, life-threatening response caused by infection.

**Diagnostic Criteria for Sepsis and Septic Shock for Pregnant and Postpartum Women**
The new definition establishes that the combination of **infection + organ damage** is the basis for diagnosing maternal sepsis, as is the case for adults. However, the WHO did not define diagnostic criteria due to concerns that the adult organ failure assessment system (SOFA score) is insufficiently validated for maternal sepsis, considering the physiological changes specific to pregnancy and its complexity. In the absence of WHO diagnostic criteria, the Lombardy Region Technical Advisory Group (GAT) has proposed using diagnostic criteria already used for the adult population, adapted to the physiological conditions of pregnancy. This strategy is in line with recommendations from the Obstetric Medicine Association of Australia and New Zealand.

Therefore, based on these definitions:

- **Maternal Sepsis** is a life-threatening condition defined as organ dysfunction resulting from an infection during pregnancy, childbirth, post-abortion, or postpartum period.

**Suggested Diagnostic Criteria**
The diagnosis of maternal sepsis involves the identification of a suspected or confirmed infection associated with the damage of one or more organs, which can be evidenced by one or more of the following criteria:

- Need for oxygen to maintain SpO2 > 95% or PaO2/FiO2 < 400
- Platelet count < 100 x 10^6/L
- Bilirubin level > 1.2 mg/dL
- Systolic blood pressure (SBP) < 90 mmHg
- Patient awakens only with verbal/pain stimulus or is unconscious
- Creatinine level > 1.2 mg/dL

These criteria are summarized by an increase in the modified SOFA score of ≥1 (Table 5).

### ****Table 5: Modified SOFA Score****

| **Organ/System** | **Parameter** | **Score 0** | **Score 1** |
| --- | --- | --- | --- |
| **Respiratory** | Oxygen requirement to maintain SpO2 > 95% | No | Yes |
| **Coagulation** | Platelet count x 10^6/L | > 100 | < 100 |
| **Hepatic** | Bilirubin level mg/dL | < 1.2 | > 1.2 |
| **Cardiovascular** | Systolic blood pressure (SBP) mmHg | > 90 | < 90 |
| **Neurological** | AVPU scale (Awake, Verbal, Pain, Unconscious) | A (Alert) | V, D, or unconscious |
| **Renal** | Creatinine level mg/dL | < 1.2 | > 1.2 |

The **SOFA (Sequential Organ Failure Assessment)** score is a system for assessing organ dysfunction in critically ill patients based on vital parameters and biochemical tests. It assigns a score (0-4) to each organ system (respiratory, cardiovascular, coagulation, hepatic, renal, and neurological), based on its level of dysfunction. The total SOFA score (0-24) is the sum of individual system scores, with a baseline score of 0 indicating no organ dysfunction.

For the obstetric population, the regional working group, pending the diagnostic criteria from the WHO, has proposed diagnostic criteria for organ dysfunction summarized in a **modified SOFA score (mSOFA)**. This strategy aims to provide:

1. An easy-to-use tool outside of critical care, as calculating the GCS or obtaining an arterial blood gas on initial evaluation in suspected infection cases outside of critical areas can be difficult.
2. A means for early diagnosis in a population where clinical progression can be rapid and often fatal.
3. A score that takes into account the physiological changes typical of pregnancy.

The modified SOFA score uses values corresponding only to **score 0** (physiological values in the obstetric population) and **score 1** (threshold pathological values indicating possible organ damage related to sepsis in the obstetric population), making it easier to use (including for memorization). Values higher than those corresponding to score 1 will be considered pathological.

**Modified SOFA Score Interpretation**

- In the obstetric population, a score of ≥1 in the modified SOFA score (rather than ≥2 as in the adult population) serves as the diagnostic criterion for identifying organ dysfunction related to sepsis in the presence of a confirmed or suspected infection. This approach is supported by the physiological changes induced by pregnancy and the rapid clinical progression of sepsis in this population, offering a more sensitive and earlier screening tool.
- The modified SOFA score defines specific threshold values for the obstetric population. For example, baseline creatinine levels are significantly lower, with normal values ranging from 0.4-0.9 mg/dL, and a threshold of 1.2 mg/dL is considered indicative of severe organ dysfunction (score 1). Similarly, bilirubin levels follow the same pattern.

The modified SOFA score simplifies the evaluation of the respiratory and neurological systems. The Glasgow Coma Score (GCS), which is not routinely used in obstetrics and gynecology wards, is replaced by the AVPU scale (Alert, Verbal, Pain, Unconscious), which is also used in the MEOWS monitoring system. The PaO2/FiO2 ratio, which requires an arterial blood gas analysis, can initially be replaced by the "need for oxygen to maintain a saturation > 95%". These evaluations should later be confirmed by the GCS and PaO2/FiO2 ratio once the patient is under the care of an anesthesiologist/intensivist or potentially admitted to a critical care unit.

In obstetrics, a systolic blood pressure (SBP) < 90 mmHg can be normal, so the modified SOFA score should be interpreted considering the patient's usual pressure values during pregnancy or the puerperium.

### ****Septic Shock****

Septic shock is a subset of sepsis where circulatory, cellular, and metabolic alterations are associated with a higher risk of mortality compared to sepsis alone.

**Suggested Diagnostic Criteria for Septic Shock**
The diagnosis of septic shock involves confirming an infection associated with hypotension requiring vasopressor administration to maintain a mean arterial pressure (MAP) ≥65 mmHg and a lactate level >2 mmol/L after appropriate volume resuscitation.

**Quick-SOFA (qSOFA)**
Due to the difficulty of calculating the SOFA score outside of Intensive Care Units, the authors of the new guidelines in the adult population proposed a simplified surrogate, the qSOFA. The qSOFA, where "quick" refers to its rapid use at the bedside without laboratory tests, investigates three simple parameters: blood pressure, respiratory rate, and mental status. If at least 2 criteria are met—tachypnea (RR ≥22 breaths/min), hypotension (SBP ≤100 mmHg), and altered mental status—the patient with suspected or confirmed infection is at risk of hospital mortality or prolonged critical care unit admission.

However, for the obstetric population, the regional working group does not suggest using qSOFA for the following reasons:

1. It has not been validated for the obstetric population.
2. The threshold values for the variables are not applicable to pregnant patients (e.g., baseline respiratory rate is higher, and systolic blood pressure is lower compared to the adult population).
3. Literature suggests that the sensitivity of EWS is higher than qSOFA in identifying patients at risk of negative evolution.
4. It is preferable to use a tool that identifies negative evolution risk not only among infected patients.

## How to identify and diagnose Sepsis/Septic Early

The diagnosis of sepsis/septic shock starts with a high index of clinical suspicion by the healthcare professional. The diagnosis of sepsis/septic shock must be suspected in the presence of one or more of these elements according to the algorithm shown in Figure 1:
- presence of risk factors for infection/sepsis (predisposition) (Table 1),
- signs and symptoms of infection,
- alteration of vital parameters,
- evidence of organ damage,
- foetal bradycardia or tachycardia, foetal or perinatal death, miscarriage or premature labour.

In clinical practice:
**- Predisposition:** Their recognition and traceability on clinical documentation facilitate the eventual diagnosis of infection/sepsis in the case of an as yet ill-defined clinical picture. At every hospital admission and at periodic visits during hospitalisation, it is therefore essential to assess the presence/new occurrence of risk factors for infection/sepsis (Table 1); this information must be recorded in the clinical documentation and clearly visible to all healthcare professionals involved in the pathway of the pregnant/pubertal patient.
**- Clinical suspicion of infection**: Infections in pregnancy are not uncommon and are potentially dangerous for mother and foetus. Clinical signs and symptoms of infection vary depending on the site and are often subtle due to the physiological changes typical of pregnancy. At each assessment, it is essential to take a thorough history (symptoms reported by the patient) and/or a careful clinical examination (signs/symptoms noted by the provider) to identify the possible presence of infection (Table 6).

In case of suspected or established infection, always consider the suspicion of sepsis/septic shock by looking for signs/symptoms of possible organ damage, first by assessing vital signs using the MEOWS score and blood tests, and then to confirm the diagnosis using the diagnostic criteria for organ damage (modified SOFA score).

| **Table 6. Clinical signs and symptoms suggestive of infection in pregnant women and during the puerperium:** |
| --- |

| Hyperpyrexia or hypothermia |  |
| --- | --- |

| Flu-like symptoms |  |
| --- | --- |

| Headache and/or neck stiffness and/or confusion/disorientation |  |
| --- | --- |

| Diarrhoea and/or vomiting |  |
| --- | --- |

| Skin rash |  |
| --- | --- |

| Abdominal/pelvic pain and tension |  |
| --- | --- |

| Purulent or foul-smelling vaginal discharge |  |
| --- | --- |

| Purulent or foul-smelling amniotic fluid |  |
| --- | --- |

| Signs of mastitis |  |
| --- | --- |

| Signs of cellulitis/surgical wound infection/perineal infection |  |
| --- | --- |

| Respiratory distress (increased respiratory rate and/or use of accessory muscles and/or hypoxemia) |  |
| --- | --- |

| Respiratory symptoms (productive cough, sore throat, etc.) |  |
| --- | --- |

| Urinary symptoms (dysuria, cloudy urine, etc.) |  |
| --- | --- |

| Signs of infection associated with an intravascular catheter (oedema, redness, etc.) |  |
| --- | --- |

| Signs of foetal or neonatal infection |  |
| --- | --- |

| Leucocytosis or leukopenia |  |
| --- | --- |

| Increased inflammatory markers (CRP, PCT) |
| --- |

**- Vital Parameter Alterations:** An alteration in vital parameters detected through the MEOWS alert system is often the first sign of infection/sepsis. In the presence of ≥2 yellow or ≥1 red MEOWS parameters, always consider the possibility of infection/sepsis/septic shock by searching for signs and/or symptoms related to suspected or confirmed infection and organ damage. In a patient with infection/sepsis/septic shock, some MEOWS parameters can themselves indicate organ damage, such as systolic blood pressure, respiratory rate/SpO2, or neurological evaluation. Therefore, it is essential to evaluate vital parameters using the MEOWS alert system during each obstetric patient assessment.
**- Presence of One or More Organ Damages:** Organ damage related to sepsis results from the body's uncontrolled response to infection. This is a dangerous event for both the mother and fetus and is often the first indication of sepsis that reaches the healthcare provider. Organ damage is suspected in the presence of abnormal MEOWS vital parameters (≥1 yellow and/or red parameter) and/or changes in laboratory tests (creatinine levels, bilirubin, and platelet count to identify renal, hepatic, and coagulation damage, respectively). If these alterations meet organ damage criteria (SOFA score modification ≥1) in the presence of certain or suspected infection, the diagnosis of sepsis is made. In the presence of unexplained organ damage, it is critical to promptly investigate infection to identify or rule out potential sepsis/septic shock.
**- Foetal and Perinatal Concerns:** Bradycardia or fetal tachycardia, fetal or perinatal death, abortion, or preterm labor can all result from maternal infection/sepsis. In these cases, it is crucial to promptly search for infection and organ damage to either exclude or confirm the diagnostic suspicion.

**Summary:**

- In clinical practice, it is essential to search for signs/symptoms of organ damage in all patients with suspected or confirmed infection, and in all patients with unexplained organ damage (e.g., hypotension or altered sensorium), search for signs/symptoms of suspected or confirmed infection to confirm or exclude the diagnosis of sepsis/septic shock.
- In patients with abnormal vital parameters (according to MEOWS: ≥2 yellow or ≥1 red), it is important to search for signs/symptoms of infection and potential organ damage to confirm or exclude the diagnosis of sepsis/septic shock.
- In all patients with suspected or confirmed infection, organ damage should be assessed using the MEOWS alert system and confirmed with hematological tests (sepsis panel) and SOFA score modification.

Figure 1: Diagnostic Algorithm for Sepsis/Septic Shock. *[Translator’s note: the text in the image is in Italian, but its content is already explained in the paragraph]*

## RISK IDENTIFICATION

In the case of a pregnant or postpartum woman arriving at the delivery point or already admitted, with suspected infection, the diagnostic-therapeutic care pathway should include the following:

- Evaluation by the Midwife and the on-call Gynecologist,
- Monitoring of vital signs according to the MEOWS alert/monitoring system,
- Fetal evaluation.

Subsequent actions are defined based on the detection of a clinical deterioration alarm identifiable by the MEOWS color code. The MEOWS color code identifies a risk level with the corresponding operational procedures in the presence of suspected or confirmed infection (Figure 2).

**LOW RISK (MEOWS 1 YELLOW PARAMETER AND SUSPECTED OR CONFIRMED INFECTION)**

The low-risk condition is defined by the presence of suspected or confirmed infection and the detection of 1 yellow parameter according to MEOWS.

In this condition, the Gynecologist evaluates the vital signs, fetal well-being, and determines whether laboratory tests ("sepsis panel": complete blood count with formula, lactate, electrolytes, blood urea nitrogen, creatinine, bilirubin, PT-PTT, CRP or PCT) and/or radiological exams are necessary, as well as any required specialist consultations. Therapeutic treatment is initiated based on the patient's clinical condition. Monitoring of vital signs should continue according to the MEOWS algorithm.

**INTERMEDIATE RISK (MEOWS 2 YELLOW OR 1 RED PARAMETERS AND SUSPECTED OR CONFIRMED INFECTION)**

The intermediate-risk condition is defined by the presence of suspected or confirmed infection and the detection of 2 yellow parameters or 1 red parameter according to MEOWS.

In this condition, the diagnostic-assistance pathway includes:

- Monitoring of vital signs according to the MEOWS algorithm,
- Support for vital functions where necessary,
- Laboratory tests “sepsis panel”: complete blood count with formula, lactate; electrolytes, blood urea nitrogen, creatinine, bilirubin, PT-PTT, CRP or PCT,
- Assessing for organ damage (diagnostic criteria for organ damage or modified SOFA score),
- Performing the "Sepsis Six",
- Fetal monitoring,
- Any specialist consultations,
- In the case of a Gynecologist working at a Spoke center, consideration of transferring the patient to an Hub center.

If there are no diagnostic criteria for organ damage (modified SOFA score <1) and the serum lactate value is <2 mmol/l, the patient is placed under observation, and signs/symptoms of infection and vital parameters are monitored according to the MEOWS algorithm. Based on clinical assessment and diagnostic test results, the doctor will determine the most appropriate therapeutic plan and any need for specialist consultations during clinical observation.

If there are one or more diagnostic criteria for organ damage (modified SOFA score ≥1) or the serum lactate value is ≥2 mmol/l, proceed as with high risk (see below).

**HIGH RISK (MEOWS >2 YELLOW OR >1 RED OR ≥1 ORGAN DAMAGE CRITERION OR LACTATE >2 WITH SUSPECTED OR CONFIRMED INFECTION)**

The high-risk condition is defined by the presence of suspected or confirmed infection associated with one of the following conditions:

- 2 yellow parameters and/or >1 red parameter detected in vital signs according to MEOWS,
- ≥1 diagnostic criteria for organ damage (modified SOFA score ≥1),
- Serum lactate value >2 mmol/l.

In this situation, the patient is jointly evaluated by the Anesthesiologist, Gynecologist, and if possible, the Infectious Disease Specialist (if not available, an Internal Medicine doctor and/or Infectious Disease Specialist consultation from the Hub center). The diagnostic-therapeutic care pathway should include the following (as in intermediate risk):

- Monitoring of vital signs according to the MEOWS algorithm,
- Support for vital functions where necessary,
- Laboratory tests “sepsis panel”,
- Assessing for organ damage (diagnostic criteria or modified SOFA score),
- Performing the “Sepsis Six” (Chapter 4),
- Fetal monitoring,
- Any specialist consultations,
- In the case of a Gynecologist working at a Spoke center, consideration of transferring the patient to the Hub center,
- Consideration for admission to a critical care unit.

**Figure 2**. Tool for risk identification in the case of suspected or confirmed maternal infection. [Translator’s note: modified English version of the image included in the original Operational Guidance Doc]


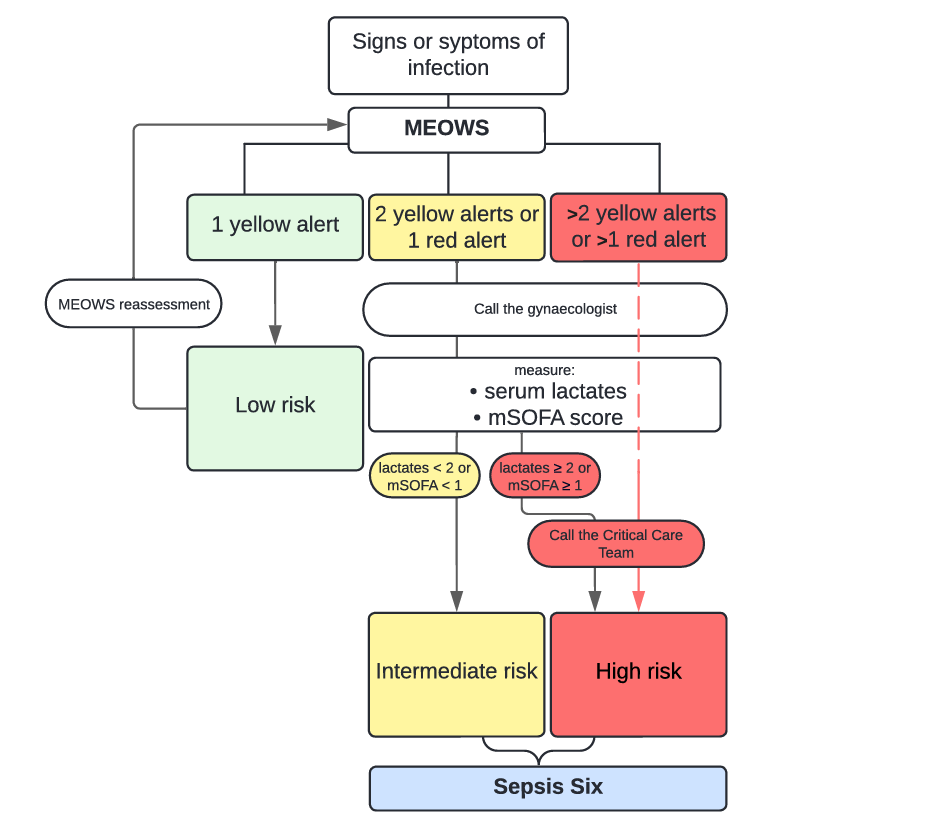


**Recommendations**

- At every obstetric assessment of the patient, whether for a new hospital admission or during hospitalization, always check for signs of organ damage in all patients with suspected or confirmed infection (e.g., hypotension or altered sensorium, etc.), and in all patients with organ damage, check for infection to confirm or exclude the diagnosis of sepsis/septic shock. In patients with altered vital parameters according to MEOWS (≥2 yellow parameters or ≥1 red), check for signs/symptoms of potential infection and organ damage.
- To identify the presence of an ongoing infection, conduct a thorough medical history (symptoms reported by the patient) and/or a careful clinical examination (signs/symptoms detected by the clinician).
- Identifying possible organ damage initially involves detecting one or more altered vital parameters (e.g., ≥1 yellow MEOWS parameter) and/or alterations in laboratory tests (creatinine, bilirubin, and platelet count). Only if these alterations in vital parameters and/or biochemical tests meet the diagnostic criteria for organ damage (modified SOFA score ≥1) in the presence of confirmed or suspected infection, the diagnosis of sepsis is made.
- In the case of suspected or confirmed infection, always assess the risk condition based on the MEOWS color code:
  - Low risk: suspected or confirmed infection + 1 yellow MEOWS parameter;
  - Intermediate risk: suspected or confirmed infection + 2 yellow or 1 red MEOWS parameters; this condition always requires the evaluation of diagnostic criteria for organ damage (modified SOFA score), serum lactate measurement, and the implementation of the Sepsis Six;
  - High risk: suspected or confirmed infection + >2 yellow or >1 red MEOWS parameters or the presence of ≥1 organ damage criterion (modified SOFA score ≥1) or lactate >2 mmol/l; this condition always requires all interventions of intermediate risk and joint evaluation by the anesthesiologist-intensivist, gynecologist, and, if possible, the infectious disease specialist.
- Follow the diagnostic-therapeutic-assistance pathway defined by the risk level based on MEOWS and/or the presence of one or more organ damage criteria (modified SOFA score) and/or lactate levels.
- Laboratory tests (sepsis panel) should be requested in all cases of intermediate and high risk and based on medical evaluation in low-risk cases.

**Actions**

- Involve relevant multidisciplinary expertise early based on the clinical scenario (Gynecologist, Anesthesiologist-Intensivist, Infectious Disease Specialist).
- In cases of suspected or confirmed sepsis/septic shock, assess the need for admission to a high-intensity care area and/or transfer to an Hub facility.
- In suspected or confirmed sepsis/septic shock, the Gynecologist and Anesthesiologist-Intensivist must inform the patient (if clinically appropriate) and/or the partner or closest family member. This communication should include details about the clinical condition and the potential risks to both the mother and fetus related to the condition.
- Establish a system for identifying and framing the case using a decision-making algorithm (thresholds for re-evaluation, alert levels, and risk levels) suitable for obstetric patients with suspected infection/sepsis/septic shock.
- Define action and intervention procedures based on different alert and risk levels.
- Establish a shared procedure with diagnostic criteria for sepsis (organ damage) and septic shock in the obstetric population.
- Implement standardized communication systems between healthcare providers (midwives and doctors) for prompt and complete communication of patient status (using the Situation, Background, Assessment, Recommendation – SBAR method).
- Set up a training and periodic update program on alert systems, diagnostic criteria for maternal sepsis/septic shock, diagnostic-therapeutic pathways, and the potential progression and lethality of these clinical conditions for all healthcare professionals involved in the care of pregnant/postpartum patients.

# 4. First Diagnostic-Therapeutic Interventions for Maternal Sepsis

**Rationale**

Clinical studies have shown that timely treatment of sepsis/septic shock in the obstetric population plays a crucial role in improving prognosis and reducing mortality. The use of standardized diagnosis and treatment protocols enables early and effective intervention.

Managing sepsis in pregnant patients does not appear to differ from managing it in the general adult population, provided the physiological changes of pregnancy and fetal well-being are considered. Fetal vitality assessment is particularly relevant, as the balance between oxygen supply and consumption in the fetus is often altered in cases of maternal sepsis. Maternal stabilization is therefore the best approach to support fetal vitality.

The Royal College of Obstetricians and Gynaecologists endorses the use of evidence-based recommendations proposed by the Surviving Sepsis Campaign (SSC 2016) for the management of maternal sepsis, even though the obstetric population was not specifically included in these guidelines. The primary diagnostic-therapeutic interventions (“resuscitation bundle”) proposed by the SSC have been adapted by the Surviving Sepsis Organization into a simple and effective approach known as "Sepsis Six" (a memorable acronym summarizing the “Six things to do in a septic patient”). The Sepsis Six framework, now an international reference in the initial management of sepsis, involves implementing 6 interventions within the first hour of recognizing/suspecting sepsis, consisting of: 3 diagnostic (blood culture collection, lactate measurement and other lab tests, urine output monitoring) and 3 therapeutic (oxygen administration, fluid administration, and antibiotic therapy), as shown in Figure 9.

The Sepsis Six approach, now validated and widely used in delivery settings in English-speaking countries (NHS Foundation), takes into account not only proven diagnostic-therapeutic interventions but also a clinical assessment of the patient, similar to the ABCDE approach. Therefore, the Regional Working Group recommends using the Sepsis Six for the diagnostic-therapeutic management of all cases of maternal sepsis, confirmed or suspected, with specific “precautions” related to the unique aspects of the obstetric population.

**Recommendations**

- Implement all Sepsis Six interventions in all cases of suspected or confirmed maternal sepsis or septic shock.
- Specifically, implement the Sepsis Six in all patients with:
  - **Intermediate risk**: signs/symptoms of infection + 2 yellow or 1 red MEOWS parameters;
  - **High risk**: signs/symptoms of infection + >2 yellow or >1 red MEOWS parameters or mSOFA ≥1.

The implementation of Sepsis Six in low-risk patients should only be considered on medical advice.

- Implement all Sepsis Six interventions within 1 hour of diagnostic suspicion.
- The implementation of Sepsis Six must involve a multi-professional and multidisciplinary approach; the core team must always include the Midwife, Gynecologist, Anesthesiologist-Intensivist, and Infectious Disease Specialist. Involvement of other specialists should be assessed on a case-by-case basis.

Figure 9. Sepsis Six

**Sepsis Six:**

3 Diagnostic Actions:
- Blood culture collection and other relevant cultures
- Lactate measurement and other laboratory tests (e.g., sepsis panel)
- Urine output monitoring

3 Therapeutic Actions:
- Evaluation of the need for oxygen therapy and/or ventilatory support
- Empiric antibiotic therapy
- Volume resuscitation

**Actions**

- Prepare an explanatory operating procedure on the indications, methods and timing of Sepsis Six implementation.

- Prepare a training and periodic update programme on the first diagnostic-therapeutic interventions (Sepsis Six) to be implemented in cases of suspected maternal sepsis/sepsis shock for all healthcare professionals involved in the care of the pregnant/puperium patient.

## BLOOD CULTURES AND OTHER SPECIMENS COLLECTION

Blood cultures (at least 2 sets of cultures for aerobic and anaerobic germs) and possibly other cultures depending on the suspected site of infection must be performed before starting antibiotic treatment. Their performance must in no way delay the administration of antibiotic therapy.

**Rational**

Microbiological examinations are essential for the aetiological diagnosis and modulation of empirical antibiotic therapy for sepsis. Identification of the pathogen promotes a better prognosis in cases of sepsis and septic shock; the strategy of de-escalation of antibiotic therapy reduces costs and the emergence of resistance and adverse effects. Blood culture is the essential diagnostic element for clinical diagnosis. All indicated cultures (blood, urine, sputum, faeces, material from collections, drains, devices, early urinary antigens, other) must be collected prior to the start of antibiotic therapy. The performance of culture examinations must not lead to an excessive delay (>45 min) in the initiation of antibiotic therapy. The methods of collection, storage, dispatch (‘pre-analytical phase’) and processing are essential to ensure reliable and useful results.

**Recommendations**

- **Before starting antibiotic therapy**, perform at least two sets of blood cultures (2 sets of cultures = 4 bottles), including one percutaneously and at least one from each vascular access (if in place for >48 hours). If antibiotic therapy has already started, it is advisable to perform the culture collection before the next antibiotic dose.
- Conduct cultures for all potential anatomical infection sites identified through clinical examination and patient history (e.g., urine culture, deep wound swab, or sample from purulent material, throat swab for influenza, sputum, urinary antigens for Legionella and Pneumococcus, cerebrospinal fluid, placental culture, vaginal secretions, etc.). Samples should be collected, stored, and sent according to local procedures.
- Ensure that appropriate cultures can be performed in all care units (including delivery rooms and operating rooms) and emergency departments (both general and obstetric). The site of care should not delay the collection and sending of culture tests.

**Actions**

- Develop specific operating instructions for performing culture tests (blood cultures and other relevant cultures), including sample collection, storage, sending from departments (including delivery and operating rooms), acceptance by the laboratory, processing, and communication of results. This should also be ensured in case the patient is transferred to another department.
- Create procedures for urgent processing of culture tests (e.g., blood cultures and other tests), ensuring they are available 7 days a week.
- Consider using validated technologies that reduce response times where possible.

## Lactates, Haemoglobin, and Organ Function Parameters

**Rationale:**

In septic populations, the early measurement of lactate levels plays a key role in risk stratification and therapeutic management. Elevated lactate levels indicate anaerobic metabolism, often due to tissue hypoperfusion, and are associated with a higher risk of clinical deterioration and poor prognosis. In obstetric patients showing signs or symptoms of infection, a lactate level >2 mmol/L defines the patient as high risk. A lactate level >2 mmol/L combined with severe refractory hypotension requiring vasopressor support to maintain a MAP ≥65 mmHg defines maternal septic shock. Lactate values, both absolute and in trend, are crucial for assessing the effectiveness of treatment, especially during volume resuscitation.

**Recommendations:**

- Measure lactate levels in all obstetric patients with signs and/or symptoms of suspected or confirmed infection, particularly those at **intermediate risk** (infection symptoms + 2 yellow or 1 red MEOWS) and **high risk** (infection symptoms + >2 yellow or >1 red MEOWS or modified SOFA ≥1).
- Lactate measurement should be done via arterial or venous access, preferably using point-of-care methods, to obtain results within 30 minutes.
- Measure organ function parameters (sepsis panel: complete blood count with differential, lactates, electrolytes, urea, creatinine, bilirubin, PT-PTT, CRP or PCT) to detect any organ damage not clinically visible and to calculate the modified SOFA score, which helps in diagnosing sepsis.
- **Transfusion of packed red blood cells** in cases of sepsis/septic shock (absent other conditions like myocardial ischemia, severe hypoxia, or massive hemorrhage) is recommended when hemoglobin levels fall below 7 g/dL.

**Actions:**

- Establish a procedure for point-of-care lactate measurement in all birth centers.
- Set up procedures for urgent laboratory test requests (including sample collection, sending, result communication, and recording) and, where possible, create a “sepsis panel” for easier test requests.

## URINE OUTPUT MONITORING

**Rationale**

Hourly urine output is a sensitive measure of renal perfusion. Oliguria or anuria, with output <0.5 ml/kg/h, may indicate impaired renal perfusion due to hemodynamic compromise. These conditions could be early signs of septic renal damage, which can be confirmed by renal function tests.

**Recommendations**

- Monitor urine output in all obstetric patients with suspected or confirmed infection/sepsis/septic shock.
- Place a urinary catheter in all obstetric patients with sepsis or septic shock to monitor hourly urine output.

## OXYGEN THERAPY

**Rationale**

Sepsis physiology is characterized by increased metabolic oxygen demand but reduced availability and utilization at the peripheral level due to reduced oxygen transport and extraction. Supplemental oxygen therapy helps improve oxygen transport, correcting potential arterial desaturation. The treatment goal is to achieve an arterial oxygen saturation (SpO2) >94%, which is a threshold to prevent fetal hypoxia, which can have severe consequences.

**Recommendations**

- Evaluate and monitor respiratory function (respiratory rate and SpO2) in all patients with suspected or confirmed sepsis.
- Administer oxygen therapy (humidified) with 100% FiO2 via a non-rebreathing face mask with a reservoir in obstetric patients with suspected or confirmed sepsis.

**Actions**

- Develop an operating procedure for oxygen therapy administration and respiratory function monitoring.

## ANTIBIOTIC THERAPY

**Rational**

Timely administration of appropriate antibiotic therapy within the first hour of sepsis identification and after appropriate cultures have been taken is essential for effective treatment: each hour of delay is associated with a significant increase in mortality. Initial empiric antibiotic therapy is based on clinical and epidemiologic criteria and usually includes one or more drugs with a broad spectrum of action, active against possible pathogens (bacteria and/or fungi), at effective dosages and with characteristics that ensure tissue penetration into the infection foci. In particular, the choice of antibiotic scheme must take into account the following: the main septic source in the obstetric setting is the genital tract, followed by the urinary tract and wounds (Table 11, Annex 4); from international studies, the microorganisms most frequently isolated in the obstetric setting are E. coli, Group B Streptococcus and anaerobic germs; polymicrobial infections (Gram positive and negative) are often present (Table 12, Annex 4). In Italy, the rate of carbapenem-resistance in enterobacterales, due to spread of carbapenemases and extended-spectrum ß-lactamases (ESBLs), is higher than the European average and with an increasing trend in recent years. Considering the high mortality associated with inappropriate initial treatment (in the general population a reduction in survival of up to 5-fold was estimated if empiric antibiotic therapy in septic shock did not cover the pathogen involved), it is considered preferable that the chosen treatment scheme err in the direction of “over-inclusiveness.” In order to facilitate the choice of the most appropriate empirical antibiotic therapy, the spectrum of action of the most commonly used antibiotics for the treatment of patients with sepsis/septic shock is shown in Figure 10.

Figure 10. Spectrum of action of the main antibiotics used in the treatment of sepsis in obstetrics.

Modified from Royal College of Obstetricians and Gynaecologists (RCOG) Sepsis in Pregnancy, Bacterial (Green-top Guideline No. 64a), 2012

*[Translator’s note: This guideline has been archived and are not actually available online. Here you can find the table about antimicrobial spectrum in the replacement guideline, Green-top Guideline No. 64 Identification and management of maternal sepsis during and following pregnancy, 2024.]*


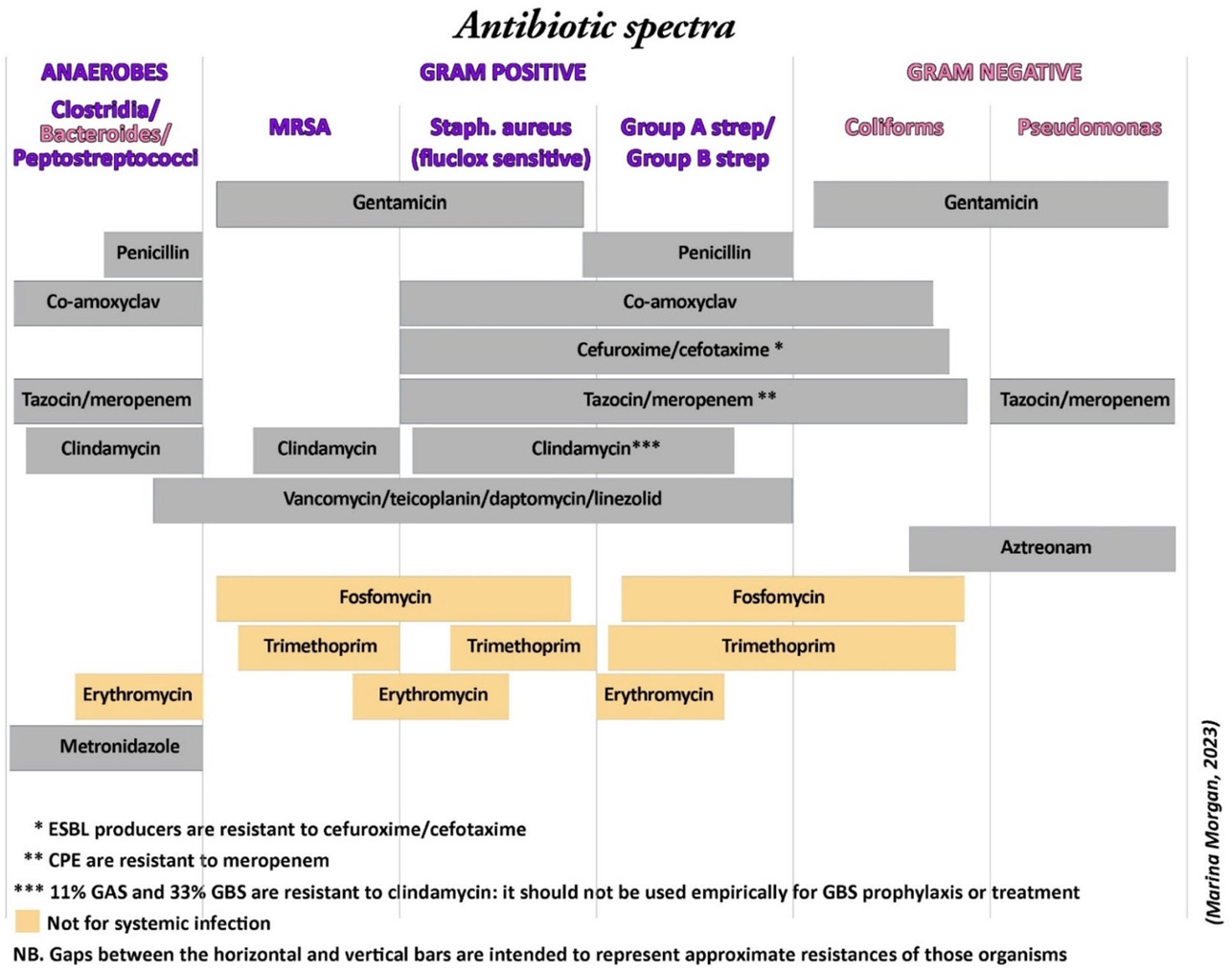


Table 13 (Appendix 4) shows the standard dosages for intravenous administration of the different drugs used in empiric antibiotic therapy of sepsis/septic shock. Recall the importance of always administering the appropriate loading dose and maintenance dose to achieve effective plasma concentrations, as failure to achieve peak plasma concentrations is associated with the risk of clinical failure.

Some proposed empirical antibiotic treatment schemes for sepsis and septic shock in the obstetric patient are shown in Table 7. Please refer to Appendix 4 for details on the choice of antibiotic regimen, its duration and modulation.

Table 7. Antibiotic regimens to be considered for empirical antibiotic treatment of sepsis/septic shock in the obstetric patient with a genital tract, urinary tract or wound infection.

| Antibiotic | Dosage | Notes |
| --- | --- | --- |
| Piperacillin/tazobactam + Gentamicin or  Amikacin | - 4.5 g every 6 h  - 5 mg/kg/day in a single administration  - 15mg/kg/day in a single administration |  |
| Meropenem +  Gentamicin or  Amikacin | - 1 g every 8 h  - 5 mg/kg/day in a single administration  - 15mg/kg/day in a single administration | Only in case of recent infection with ESBL bacteria, colonization condition by ESBL bacteria or recent fluoroquinolones and/or cephalosporins exposure |
| Ciprofloxacin +  Gentamicin ±  Metronidazole (for anaerobes) | - 400 mg every 8-12 h  - 5 mg/kg/day in a single administration  - 1g loading dose, then maintenance 500 mg every 6h | If severe allergy to penicillin (anaphylaxis: bronchospasm, angioedema, hypotension) |
| Piperacillin/tazobactam + Gentamicin +  Daptomycin or  Vancomycin | - 4.5 g every 6 h  - 5 mg/kg/day in a single administration  - 8 mg/kg/day in a single administration  - 25 mg/kg loading dose, then 500 mg  every 6 h | If suspected MRSA infection (see text). |

**Recommendations**

- Administer intravenous antibiotic treatment as soon as possible and in any case within the first hour in all cases of suspected or established sepsis/septic shock and more specifically in intermediate and high-risk patients.

- Empiric treatment should be initiated with one or more broad-spectrum antibiotics that can cover the pathogens most likely to be involved in the etiology of sepsis or septic shock.

- Once the responsible pathogen is identified and an antibiogram is obtained and/or clinical improvement is documented, antibiotic treatment should be modulated.

**Actions**

- Prepare local empiric antibiotic therapy protocols with specification of dosages, mode of administration, duration and remodulation (de-escalation).

- Verify ready availability or on-call availability of antibiotics at possible sites for 24/7 use.

- Set up procedures for consulting Infectivologist (active guard or on-call) and where not possible Physician Internist and/or consulting Infectivologist Hub center.

## SOURCE CONTROL

**Rational**

Source control of the septic focus, with its eradication where indicated, is essential for the successful treatment of sepsis and septic shock. Therefore, it is necessary to promptly identify the septic focus by the most appropriate physical examination and imaging diagnostics in relation to the clinical suspicion. The different stages of pregnancy and puerperium can also point to the possible septic focus and suggest the correct diagnostic-therapeutic course; as an example: in all cases of sepsis in puerperium it is essential to perform pelvic ultrasonography to identify collections or presence of material in the cavity, in patients diagnosed with endometritis to exclude the presence of pelvic abscesses or uterine microabscesses, the diagnosis of necrotizing vulvitis represents a 'surgical emergency etc. The main procedures for controlling septic focus in the obstetric setting are shown in Table 8.

Table 8. Main procedures for control of septic outbreak in obstetric settings.

| 1) evacuation of retained products of conception |
| --- |
| 2) wound bed sanitation (debridement) in wound infections and soft tissue infections |
| 3) drainage of abscesses |
| 4) stenting or percutaneous nephrostomy for obstructive pyelonephritis |
| 5) delivery in cases of chorionamnionitis |
| 6) hysterectomy if myometrial necrosis |

**Recommendations**

- Identify and perform source control with eradication of the septic foci where indicated, within 6- 12 hours of the diagnosis of sepsis/septic shock.

- Achieve source control using the least invasive and most risk-beneficial eradication technique (drainage, debridement, removal of potentially infected devices) particularly in pregnant patients.

- If the source control is performed, take samples of the infected biological material (biological fluids or tissue biopsies) and send it for culture examination.

- Always consider possible non-obstetric septic outbreaks, (e.g., appendicitis, pancreatic abscess, intestinal infarction, influenza).

- Promptly remove venous catheters thought to be possible septic foci as soon as other vascular accesses have been placed.

- Evaluate the decision to carry out delivery in the setting of obstetric sepsis by balancing risks related to gestational age, maternal and fetal conditions. Stabilization of maternal condition is prioritized before delivery due to compromised fetal well-being, as intervention on the mother could improve the condition of the fetus.

**Actions**

- Develop appropriate diagnostic pathways for identification of septic foci (with emphasis on imaging during pregnancy) and formulation of treatment interventions.

- Establish procedures related to patient care during the different stages of diagnostics.

- Develop multidisciplinary protocols for specialized treatment for source control (surgery, radiology, interventional radiology).

- Verify the actual operational possibilities with particular reference to interventional radiology and the availability of the operating theatre and the necessary team available urgently over 24 hours and define alternative possibilities (possible transfer to a Hub center).

- Verify how the multidisciplinary team will be activated.

## VOLEMIC RESUSCITATION

**Rational**

The pathophysiology of sepsis is characterized by alterations in tissue perfusion in which hypovolemia (absolute and/or relative) plays an essential role. Timely volume replacement can correct the hypovolemia and often the hypotensive state and ameliorate the hypoperfusion dangerous to mother and fetus. The goal of fluid resuscitation is to obtain a PAM ≥ 65mmHg, this value should be considered in relation to the patient's usual pressor parameters and other indices of perfusion (diuresis and lactates). A lactate clearance of 10% in 2 hours has been shown to be a simple and adequate tool to assess the effectiveness of volemic resuscitation and the evolution of the clinical picture.

It must be remembered that in some cases fluid replacement, due to the permeabilized state typical of sepsis, may be responsible for lesional pulmonary edema resulting in respiratory distress. Such a “complication” is not a clinical error but the result of an organ conflict (heart-lung) that will need to be addressed by suspension/reduction of fluid resuscitation and respiratory assistance.

A hypotensive condition with the need to administer vasopressor drugs to maintain a PAM ≥65mmHg and a lactacidemia >2mmol/L after adequate fluid resuscitation defines a picture of septic shock; adequate volemic resuscitation is defined as the administration of 30ml/kg of crystalloids in refracted boluses of 500 ml in 1 hour. To define a patient in septic shock, it is essential to assess the response to volemic load: if arterial hypotension associated with a lactate value >2mmol/L persists, the diagnosis is septic shock; if it resolves, the diagnosis is sepsis.

It must be considered that alterations in the cardiovascular system typical of the pregnant patient have a significant impact on clinical framing and hemodynamic management in shock: 1) in pregnancy the blood volume increases up to 50% , for clinical signs of hypovolemia become evident late, only after a 30% loss of blood volume; 2) in case of maternal hypotension the placenta is the organ that responds with greater vasoconstriction with secondary fetal hypoxia and acidosis; 3) fetal distress may be the first sign of hemodynamic instability; 4) from the 20th week the gravid uterus may cause aorto-caval compression reducing venous return to the heart resulting in hypotension.

Timely hemodynamic management that is “attentive” to the peculiarities of the pregnant patient is essential to improve prognosis in this population.

**Recommendations**

- In patients with hypotension in cases of suspected or established sepsis/septic shock, perform fluid loading of 30ml/kg of crystalloids in refracted 500-mL boluses within the 1st hour; it is recommended after the 20th week of pregnancy to position the patient in left lateral decubitus to exclude aorto-caval compression as a cause/concause of arterial hypotension.

- Perform volemic resuscitation with crystalloids by alternating saline with balanced electrolyte solution; albumin use in addition to crystalloids is suggested in patients with high fluid requirements.

- Monitor the following vital parameters during volemic resuscitation: blood pressure, heart rate, hourly diuresis, SpO2, respiratory rate.

- Assess the response to volemic resuscitation both in terms of efficacy (PA improvement, peripheral perfusion: diuresis and lactates) and complications.

- Continue volemic resuscitation until the goal of a PAM ≥65mmHg is achieved for a maximum dose of 30ml/kg; additional fluid doses should be prescribed by the Reimaging Anesthesiologist if necessary.

- Monitor blood levels of lactates in all cases of hypoperfusion and overt shock to assess the response to volemic resuscitation and the evolution of the clinical picture.

- Assess fetal viability in all cases of hypoperfusion or overt shock.

**Actions**

- Preparation of a “volemic resuscitation” protocol indicating the type, amount, and mode of fluid administration, maternal and fetal monitoring, and intended goals.

- Verification of the availability of appropriate infusion systems (infusion pumps).

- Verification of the availability of the indicated solutions (quantity, quality) at the sites of use (delivery room delivery room, operating room, etc.).

- Verify procedure for requesting specialist expertise (Anesthesiologist- Resuscitator).

## VENOUS THROMBOEMBOLISM PROPHYLAXIS.

**Rational**

Sepsis in the obstetric setting is considered a risk factor for venous thromboembolism: the underlying pathogenetic mechanisms are not fully elucidated, but are believed to be the consequence of multiple factors such as immobility, activation of the inflammatory cascade, disseminated intravascular coagulation, and venous stasis.

Prophylactic doses of LMWH in the obstetrical patient (pregnancy and puerperium) are: deltaparin 5000 IU s.c. every 24 hours; nadroparin 2850 IU s.c. every 24 hours; enoxaparin s.c. 40 IU s.c. every 24 hours as defined in the guidelines of the American College of Chest Physicians (2012). At extremes of body weight, the dosage should be adjusted.

**Recommendations**

- Pharmacologic prophylaxis of venous thromboembolism with low-molecular-weight heparin (LMWH) is recommended in the septic patient, unless contraindicated to the use of this drug.

- Whenever possible, pharmacologic prophylaxis should be combined with mechanical prophylaxis (elastic stockings or intermittent pneumatic compression). Use only prophylaxis by mechanical means if there are clinical contraindications to LMWH prophylaxis.

**Action**

- Prepare protocols for thromboembolic prophylaxis in the pregnant woman and the puerpera with sepsis.

# ATTACHMENTS

## ANNEX 1. PARAMETERS OF THE MEOWS SHEETS

At each assessment, the following should be noted as basic parameters:

- respiratory rate,

- temperature,

- heart rate (maternal pulse),

- blood pressure,

- level of consciousness (AVPU score).

Other useful parameters:

1. O2 saturation,

2. diuresis,

3. appearance of the woman,

4. pain scale,

5. lochiazioni.

**RESPIRATORY RATE**

Respiratory rate represents one of the most significant parameters being the first and most sensitive indicator of the patient's deteriorating clinical condition (Johnson and Taylor, 2010). This parameter is measured by counting the respiratory acts for 30 seconds and then doubling, if the breath rate is constant, otherwise continue counting up to 60 seconds. Another method of measurement is to rest the woman's pulse on her chest and detect chest excursions.

**Recommendation**

- Respiratory rate should be taken at each assessment immediately after heart rate.

- It is important that the woman does not perceive that her respiratory rate is being taken, otherwise she may change her breathing pattern, resulting in an inaccurate reading.

- Evaluation of the respiratory system should also include objective examination (auscultation).

- Respiratory rate values considered normal in MEOWS records are between 10 and 19 respiratory acts/minute.

- Tachypnoea represents one of the earliest and most important signs in sepsis, and its presence always requires thorough clinical evaluation.

- Dyspnoea is a fairly common symptom in pregnancy, can occur in any trimester, has a gradual onset and is usually noticed by the woman when she speaks or when she lies down.

The main causes of dyspnoea in infection/sepsis/septic shock in the obstetric population are shown in Table 9.

Table 9. Main causes of dyspnoea in pregnancy in case of infection/sepsis/septic shock

| Condition | Signs | Symptoms | Investigations | Treatment |
| --- | --- | --- | --- | --- |
| Pneumonia | Tachypnoea, dyspnoea, fever, desaturation | Cough, fever, malaise | Chest x-ray, chest CT scan, EGA,  Specific haematochemical examinations | Oxygen Antibiotics  Respiratory support |
| Lesional pulmonary edema | Tachypnoea, dyspnoea  Desaturation  Heart failure | Orthopnoea, dyspnoea, foamy, pinkish sputum, chest pain | Chest x-ray,  EGA,  ECG, echocardiogram, Invasive monitoring | Oxygen, Diuretics,  Vasoactive amines,  Transfer to intensive care unit |

**OXYGEN SATURATION**

The percentage oxygen saturation levels of arterial haemoglobin depend on the efficiency of respiratory exchange in the lungs and are detectable by SpO2.

**Recommendations**

- SpO2 may not be measured routinely (it is part of the possible additional parameters) but should definitely be measured in certain circumstances:

- if the respiratory rate (RF) is within the abnormality parameters (red or yellow),

- if there is a medical/obstetric condition that could alter respiratory exchanges (e.g. respiratory disorders, intensive care).

- The accuracy of oxygen saturation assessment depends on the peripheral blood flow in the area of sensor application. If peripheral circulation is compromised or the woman's clinical condition is critical, SpO2 detection may be inaccurate or undetectable.

- Normal saturation parameters in MEOWS are between 96 and 100 per cent.

**TEMPERATURE**

The absence of fever does not exclude sepsis, as the administration of paracetamol or other antipyretics may reduce it. Conversely, “the absence of hyperpyrexia in sepsis should be cause for alertness”.

**Recommendations**

- Temperature should be taken at the most appropriate site (tympanic).

- Report the temperature taken in the MEOWS card.

- Temperature parameters considered normal in MEOWS are between 36 and 37.4°C.

- Hypothermia may be a significant finding indicative of infection and should not be ignored.

- Hyperpyrexia may be masked by the administration of antipyretics.

- In cases of hyperpyrexia consider screening for sepsis and specific antibiotic therapy

early.

**HEART RATE**

The most frequently used site for heart rate measurement is the radial artery due to its easy accessibility. The brachial artery is used in the measurement of blood pressure and the carotid and femoral artery can be assessed in the case of hypotension, when cardiac output cannot be detected in the peripheral circulation (Johnson & Taylor, 2010).

The radial artery should be palpated using the index and middle finger, resting the woman's pulse on her chest, and the heart rate should be assessed for 30 seconds and then doubled if regular, or for 60 seconds if irregular (Kozier et al, 1998).

Some instruments (Saturimeter, ECG monitor) provide a heart rate reading.

**Recommendations**

- The heart rate should be assessed for 30 seconds and then doubled if regular, or for 60 seconds if irregular (Kozier et al, 1998).

- The heart rate can be measured with electronic instruments (saturimeter, ECG monitor).

- The heart rate must be reported on the MEOWS sheet.

- The parameters considered normal heart rate are between 60 and 99 bpm.

**ARTERIAL PRESSURE**

Systolic and diastolic blood pressure values are recorded separately in the MEOWS table. It is important, in order to obtain correct blood pressure measurements, to use sphygmomanometers suitable for the patient's arm circumference. Electronic BP measurement is now widely used and reliable; however, in case of doubt, manual measurement is also recommended.

**Recommendations**

- Systolic blood pressure (PAS) and diastolic blood pressure (PAD) are reported separately to facilitate the detection of alert values.

- The pressure should be measured using a sphygmomanometer appropriate to the woman's arm size.

- Systolic blood pressure should be detected at the clearly audible I Korotkoff sound, and diastolic pressure at the V Korotkoff, when the sound is no longer detectable.

- The electronic BP reading can be inaccurate. In the case of major alterations, it is good practice to re-evaluate it by manual measurement.

- PAS values considered normal in MEOWS are between 100 and 139 mmHg, and between 50 and 89 for PAD.

**Hypotension** is a late sign of deteriorating clinical condition in the pregnant patient. Physiological hypotension in pregnancy and the puerperium may lead to a delay in early recognition of a possible haemodynamic alteration.

**Hypertension** - the conventional definition of hypertension in pregnancy is:

- Two readings of 140/90 mmHg at least 4 hours apart (NCCWCH, 2010). The AIPE guidelines adopt the definition of gestational hypertension in the presence of systolic blood pressure values >140 mmHg and/or diastolic blood pressure values >90 mmHg, in at least two consecutive measurements, at least 6 hours apart, after the 20th week of pregnancy, in a woman who is normotensive before pregnancy and before the 20th week (National High Blood Pressure Report, 2000).

- An increase of 15 mmHg above the blood pressure measured at the first visit

- A measurement of 160/100 or greater.

**DIURESIS**

**Proteinuria**: may indicate the presence of infection, underlying renal disease resulting from hypertension or may be a contaminated specimen (from fluid or vaginal secretions). Transient positive tests are usually meaningless and are due to the physiological changes of pregnancy with the appearance of minute amounts of albumin or globulin in the urine. To exclude the presence of infection, a urine culture specimen must be sent.

**Glycosuria**: this is a common finding in pregnancy due to the physiological changes that occur in kidney function. However, glucose can also appear in the urine in cases of:

- increased glucose levels (hyperglycaemia)

- changes in renal function and thus renal absorption

- transient after administration of corticosteroids e.g. betamethasone/dexamethasone.

**Recommendations**

- Urine examination by urine stick should be performed and documented in the MEOWS card on the following occasions:

- In the presence of specific maternal disorders such as hypertension, diabetes or typical signs/symptoms of hypertensive complications of pregnancy, even in the absence of hypertension.
- In the presence of clinical symptoms such as dysuria.

- The frequency of urine examination after admission depends on the clinical evaluation and the

diagnosis made.

**NEUROLOGICAL STATUS ASSESSMENT - AVPU SCALE**

The neurological response is a measure of the state of consciousness. The AVPU scale indicates:

- A - alert and oriented towards people, place, time and event (Alert).

- V - responds to voice/verbal stimuli (e.g. postoperative) (Verbal).

- P - responds to painful stimuli with voluntary or involuntary movements (Pain).

- U - non-responsive - the patient does not respond to any type of stimulus (Unresponsive).

**Recommendations**

The neurological assessment should be reported in the MEOWS card.

- Vigilant (A): white box.

- Voice response (V): yellow box.

- Pain response (P): red box.

- No response (U): red box.

Any change in the level of consciousness (AVPU scale) should always be considered significant and immediate action should be taken.

**PAIN ASSESSMENT**

An important datum that must always be included in the overall clinical picture of the woman is the assessment of pain using a pain rating scale from 0-10 (o: no pain, 10: extreme pain). Since this datum is characterised by an important subjective component, it is not included with a score in the MEOWS card, but it should always be considered.

In the Irish reference document, other pain rating scales are proposed that can be used. Figure 11 shows the pain rating scale proposed by the panel.

Figure 11. Pain rating scale

**Recommendation**

- There is no score assigned to the pain scale, but any element of concern in relation to pain reported by the woman should be cause for involvement of the midwife on duty, the gynaecologist or the anaesthetist.

## ANNEX 2. INTENSIFICATION OF MONITORING

- Intensify the frequency of measurements every 15 minutes,

- monitor O2 saturation and administer O2 with face mask,

- if prenatal, position the woman in left lateral decubitus at 15-20° and start

cardiotocographic monitoring (CTG) once the woman is stabilised,

- assess venous access,

- assess therapy sheet and correct administration of medication, report any delays

of administration, especially of antihypertensive drugs,

- ensure that experienced obstetric staff are available and assess the most appropriate place to

manage the situation,

- organise the possible transfer,

- assess the best bed position (whether lithotomic or sitting),

- verify that the environment is safe,

- verify the sending of laboratory samples/ receipt of examinations,

- bring monitoring equipment, blood-gas-analysis syringes and devices for further investigations

further investigations (e.g. culture),

- report everything in detail in the chart,

- ask the patient to report any symptoms/signs of worsening condition

clinical conditions and ensure that adequate documentation is obtained,

- inform the patient and her family about the care plan.

## ANNEX 3. IMPLEMENTATION AND OBSTACLES TO THE USE OF THE MEOWS CARD

The high workload, due to the chronic shortage of staff and the increased complexity of the obstetric population, may conflict with the implementation of the monitoring foreseen by the MEOWS card; the collection of parameters may be judged by some as "excessive" and unnecessary in a physiological condition such as pregnancy.

In order to achieve better staff compliance with the new monitoring procedure, it is certainly necessary to consider and implement organisational aspects and appropriate training of all professionals involved in the care of pregnant women.

The problems encountered in the use of the MEOWS cards are reported in Table 10.

Table 10. Perceived barriers to EWS implementation

| Perceived barriers to EWS implementation (n = 107) | n | (%) |
| --- | --- | --- |
|  |  |  |
| Overlap with partogram use | 46 | 43.4 |
| Lack of instruction/training (n = 106) | 23 | 21.7 |
| Insufficient staff to adequately complete MEOWS boards | 21 | 19.6 |
| Lack of support for MEOWS boards from midwives | 18 | 16.8 |
| Lack of support for MEOWS boards from doctors | 17 | 15.9 |
| Use of other parameter sheets | 15 | 14 |
| Lack of evidence and validation of MEOWS in midwifery care | 14 | 13.1 |
| Lack of prioritisation of other care procedures | 13 | 12.1 |
| Other | 13 | 12.1 |
| Too much time needed | 10 | 9.3 |
| Inappropriate trigger parameters for the pregnant woman | 9 | 8.4 |
| Impact on woman of frequent interruptions | 4 | 3.7 |

In 21% of the cases, delays in calling the doctor were reported, usually due to the high workload. There is often no clear delineation of the process of intensified monitoring and involvement of a multidisciplinary team. The most important barrier observed was the overlap with the compilation of the partogram (43%). Recall that the MEOWS card is not validated for the woman in labour. Therefore, for the monitoring of the woman in labour one has to stick to the protocol that does not foresee the use of MEOWS cards.

The lack of guidance/training on the use of the MEOWS card was the second acknowledged obstacle (22%) to which is added the lack of personnel.

The implementation of the use of the MEOWS card requires training for gynaecologists, midwives and anaesthetists, regarding the correct way to detect parameters and how to intensify monitoring as parameters change. In addition, audits, feedback and clinical case reviews need to be initiated.

## ANNEX 4. Choice of antibiotic regimen, remodulation and DURATION

Table 11. Pathogens most frequently involved in sepsis in the obstetric setting according to the source of infection (from Joseph et al. 2009).

| Infection that may lead to sepsis and micro-organisms most frequently involved (indicated in parantheses) | Micro-organisms most frequently causing sepsis in pregnancy/ puerperium |
| --- | --- |
| Pyelonephritis (1, 4) | 1 Escherichia coli |
| Perinephric abscess (1, 4) | 2 Bacteroides (anaerobes) |
| Pneumonia (6, 7) | 3 Clostridium (anaerobes) |
| Chorioamniositis (1, 2, 8–12) | 4 Klebsiella spp. |
| Endometritis (1, 2, 5, 9, 12) | 5 Pseudomonas aeruginosa |
| Necrotising fasciitis (2, 3, 6, 9) | 6 Streptococcus species |
| Septic abortion (1, 3) | 7 Staphylococcus aureus |
| Caesarean section wound infection (1, 2, 6, 7) | 8 Group B Streptococcus |
| Mastitis (7) | 9 Peptostreptococcus (anaerobes) |
|  | 10 Enterococcus spp. |
|  | 11 Listeria monocytogenes |
|  | 12 Enterobacter spp. |
|  |  |

Table 12. Pathogens isolated in women with sepsis at different stages of pregnancy (from Knowles et al. 2015).

|  | Pregnancy | Peripartum | Postpartum |
| --- | --- | --- | --- |
| Escherichia coli | 55% | 22% | 42% |
| Group B Streptococcus | 4.2% | 43% | 9.2% |
| Anaerobes | 8.5% | 8% | 8.5% |
| Staphylococcus | 8.5% | 5% | 9.2% |
| Enterococcus | 4.2% | 5% | 4.6% |
| Group A Streptococcus | 0 | 2% | 7.6% |
| Klebsiella | 2% | 2% | 1.5% |
| H. influenzae | 6.4% | 1% | 0 |
| Others | 11.2% | 11% | 11.2% |
| Total | 47 | 99 | 130 |

Table 13. Recommended dosages and infusion modes for antibiotics commonly used in sepsis.

| ANTIBIOTIC | STANDARD E.V. DOSE IN ADULTS DIAGNOSED WITH SEPSIS |
| --- | --- |
| Amoxicillin/clavulanate | 2.25 g q6h |
| Amikacin | 15 mg/Kg die |
| Ampicillin/sulbactam | 3 g q6h |
| Azithromycin | 500 mg die (one single administration) |
| Ceftriaxone | 2 g die (one single administration) |
| Clindamycin | 900 mg q8h |
| Ciprofloxacin | 400 mg q8-12h |
| Daptomycin | 8 mg/kg die (one single administration) |
| Gentamicin | 5 mg/kg die (one single administration) |
| Levofloxacin | 750 mg die (if body weight >70 kg 500 mg q12h) |
| Meropenem | 2 g loading dose then 1 g q6-8h (extended infusion in 4-6h) |
| Metronidazole | 15 mg/kg loading dose then 7.5 mg/kg q6h |
| Piperacillin/tazobactam | 4.5 g q6h (first dose rapid bolus then extended infusion in 4-6h) |
| Vancomycin | 25 mg/kg loading dose then 500 mg q6h |

Possible regimens (Table 7 and Table 13) for initial empirical antibiotic therapy of sepsis in the obstetric setting include a broad-spectrum carbapenem (meropenem; imipenem/cilastatin; ertapenem; doripenem) or a penicillin/beta-lactamase inhibitor combination (e.g. piperacillin/tazobactam) in combination with an aminoglycoside (e.g. gentamicin; amikacin). Combination therapy (using 2 different antibiotic classes in combination) seems biologically plausible and probably clinically useful in severe infection (particularly in septic shock) although there is no conclusive evidence of benefit in bacteremia and sepsis without shock. However, in patients with Gram-negative sepsis (pathogens prevalent in pregnancy and puerperium sepsis) it has been reported that the beta-lactam plus aminoglycoside combination significantly reduces the likelihood of inadequate initial therapy, which is an independent predictor of mortality (Micek 2010).

The increase in carbapenem-resistant Gram-negative bacteria (European Centre for Disease Prevention and Control 2017) dictates that the use of these antibiotics should only be limited in infections complicated by extended-spectrum ß-lactamase-producing bacteria -ESBL- (patients with a recent microbiologically established infection with ESBL bacteria, with a colonisation condition by ESBL bacteria or with a recent exposure to fluoroquinolones and/or cephalosporins). In the absence of these conditions, the combination of a beta-lactam + aminoglycoside is preferred for empirical therapy of sepsis in obstetrics (Elton and Chaudhari, 2015; Burton and Sibai 2012; Rhodes 2016). The initial dose of beta-lactam can be administered as a bolus or rapid infusion to rapidly reach therapeutic blood levels. After the initial dose, it appears that a prolonged/continuous infusion is more effective than intermittent dose administration.

For aminoglycosides, in the case of renal impairment it is necessary to monitor the plasma level of the drug to ensure that concentrations are low enough to minimise the risk of renal toxicity and to calibrate the interval between doses.

Due to the possibility of cross-reaction between penicillins and carbapenems (estimated at about 1 per cent of cases), in the case of anaphylactic shock or severe allergic reaction to penicillin in history, carbapenems are contraindicated. In these patients, a possible alternative is the combination of ciprofloxacin + aminoglycoside.

The therapeutic schemes suggested above are not appropriate in the case of infection with methicillin-resistant S. aureus (MRSA). In Italy, the phenomenon of community MRSA infections is considered to be much lower than in the United States, while the prevalence of MRSA in nosocomial infections is high. Since there is no specific score for assessing the risk of colonisation by MRSA in the obstetric setting, one can consider the possibility of colonisation by MRSA in the presence of one or more of the following risk factors

- exposure to previous antibiotic therapy (within 6 months) with fluoroquinolones, cephalosporins, carbapenems;

- hospitalisation or intravenous therapy within 12 months prior to delivery;

- transfer from another health facility;

- presence of a bladder catheter or vascular catheters at the time of admission to hospital.

In these cases, an anti-MRSA drug, such as vancomycin, daptomycin or teicoplanin, should be added to the treatment schedule suggested above.

Table 14 shows safety data on the main antibiotics used in the management of sepsis for use during pregnancy (reference is made to the Food and Drug Administration classification) and during lactation (data from the LACTMED database).

The rescheduling of treatment, especially when combined antibiotic therapy is initially imposed, is of benefit both to local epidemiology because it reduces the risks of developing bacterial resistance, but also to the individual patient (risks of coinfection and mortality). Patients starting empirical antibiotic therapy for sepsis or septic shock will have to be evaluated later, ideally after the first 72 hours, by the infectious disease specialist for remodulation of therapy. Once culture test results are available, eliminate unnecessary antibiotics and replace broad-spectrum antibiotics with more specific agents. Evaluation to remodulate antibiotic therapy should be done on a daily basis.

Duration of treatment: a duration of antibiotic treatment of 7-10 days is adequate for most infections associated with sepsis or septic shock. Longer treatments are appropriate for patients with a slow clinical response, with undrainable foci of infection, bacteraemia or in patients with immunodeficiency. Shorter treatments are appropriate in patients with rapid clinical resolution after control of the initial focus in abdominal or urinary sepsis. In cases of bacteraemia, 2 sets of blood cultures are recommended at 48-72 hours after the first positive blood cultures to document blood culture negativity and control of the infection.

Table 14. Safety data for use of antibiotics in pregnancy and lactation

| Antibiotic | Pregnancy (FDA Category*) | Lactation (database LACTMED) |
| --- | --- | --- |
| Clindamycin | B | Monitor the infant for gastrointestinal side effects (diarrhoea, candidiasis or rarely melena) |
| Ciprofloxacin | C | Acceptable in lactation, but recommended monitoring the infant for changes in intestinal flora (diarrhoea or candidiasis). Leaving an interval of 3-4 h between drug administration and feeding reduces the infant's exposure to the drug. |
| Daptomycin | C | Limited information, but adverse effects on neonate not expected due to high molecular weight, high protein binding and low oral bioavailability of the drug. No special precautions necessary. |
| Gentamycin | D | Considered compatible with breastfeeding |
| Meropenem | B | Despite the absence of information on the use of Meropenem during breast-feeding, beta-lactams are not expected to cause adverse effects in the infant. Monitoring of the infant for changes in intestinal flora is suggested. |
| Metronidazole | B | Earlier studies had suggested a possible mutagenic property of the drug in humans, raising concerns about exposure of infants through breastfeeding. The relevance of these findings has been questioned and there are no conclusive studies in the human species. Some sources recommend suspension of breast-feeding 12 to 24 hours after administration. |
| Piperacillin/tazobactam | B | Considered compatible with lactation |
| Vancomycin | C | Limited data indicate that vancomycin is found at low concentrations in breast milk and as the oral bioavailability of the drug is low, adverse effects on the infant are unlikely.  No special precautions are necessary. |
| * Category B: animal studies have shown no risk to the foetus, but there are no methodologically valid and controlled studies in pregnant women OR animal studies have found toxicity, which has not been confirmed by methodologically valid and controlled studies in women at first and subsequent trimesters of pregnancy.  Category C: Animal studies have found foetal toxicity and there are no methodologically valid and controlled studies in humans, however the potential benefits of the drug may justify its use in pregnant women despite the potential risks to the foetus.  Category D: Human studies and pharmacovigilance data have shown a risk to the foetus, but the potential benefits of the drug may justify its use in pregnant women despite the potential risks to the foetus. | | |

## ANNEX 5. Organisational Check-list

Considering that each organisation already has in use a Logistical-Organisational Check-list, already proposed by Regional Decree no. 7517 of 5/8/2013 ‘Integrated strategies to reduce hospital mortality associated with severe Sepsis’, it is considered important to continue with this monitoring by identifying specific indicators (Structure, Process and Outcome Indicators).

Among the process indicators, the attached Organisational Logistics Check-list has been modified to include the organisational aspects proposed by this document specific to birth points. The use of this tool allows the verification of these aspects, the analysis of critical points and the identification of possible corrective solutions (e.g. removal of barriers).

It also represents a valid benchmarking tool for regional health agencies, making it possible to create a systematic comparison and the creation of a standard of excellence in relation to performance, assessing the correct use of resources and thus defining the changes necessary for improvement.

Company_____________________________ Presidium__________________________________ LOGISTIC ORGANISATIONAL CHECK-LIST

Clinical care pathway

1) Does your hospital have a codified company procedure for the clinical classification of patients on admission (in the PS and inpatient wards) with the simultaneous definition of the decision-making algorithm (alert criteria and threshold, re-evaluation times)?

YES NO

if YES, is the use of a specific scoring system (MEWS-type alert score) envisaged?

YES NO

If YES, please indicate in which wards: PS or in-patient wards; which score: ......................

If YES, is re-evaluation of the score foreseen at codified times? YES NO

If YES, is there provision for nurses/obstetricians to carry out diagnostic and

therapeutic manoeuvres according to the protocols and confirmation of the doctor on call? YES NO

1bis) Is there in use in your (Maternity centre) department/hospital a codified procedure for clinical framing and monitoring with simultaneous definition of the decision-making algorithm (such as MEOWS score) for the obstetrical patient outside the labour phase?

a) YES NO If YES

b) Is the use of a specific score/colour system (MEOWS type) foreseen? - MEOWS (specify which version) YES NO......................

- other (specify) YES NO..................... ;

c) Not Relevant

2) Is there a specific clinical-assistance pathway within your hospital for the identification of septic patients?

YES NO

seSI

1. Is there a training programme for sharing it? YES NO
2. the relevant protocols are: - paper-based YES NO
   if YES, available in the department elsewhere - computer-based YES NO

2bis) Is there a specific diagnostic-therapeutic-assistance protocol (PDTA) for obstetric sepsis in use in your department/hospital that is available to all health care professionals at the birthplace?

a)YES NO If YES

b) What is the method of consultation of the protocol? - paper-based YES NO

if YES, available on the ward elsewhere ______________________________ - computer support YES NO ;

if YES, open to all - restricted access___________________________ - last updated:.......................

3) Is there a procedure for calling the emergency team in case of need at your hospital? SI NO

(sintetica descrizione della procedura) _______________________________________________________________________________________ _______________________________________________________________________________________ _______________________________________________________________________________________ ________________________

Laboratory

4) Does your Microbiology Laboratory accept and process blood cultures 24 hours a day, 7 days a week?

YES NO

if NO: how do you accept and process them (days and times)?

descrizione________________________________________________________________________ ___________________________________________________________________________ ___________________________________________________________________________

5) Are there any instructions for storing blood cultures prior to acceptance by the laboratory?

YES NO

6) Is a company procedure for performing blood cultures in use in your hospital?

YES NO

if YES, a) please indicate date protocol was drafted: .../..../.......; date last updated: .../..../......;

b) protocol type

- hard copy YES NO ;

if YES, available in the department elsewhere
- computer support YES NO

7) Is your Microbiology Laboratory expected to provide (a) an immediate alert in the event of a positive test with relevant bacterioscopy and Gram staining, and (b) a preliminary negative response if the blood culture is still negative at 48h?

a)YES NO b)YES NO

8) Is your Biochemistry Laboratory able to accept and process urgent examinations 7 days a week?

YES NO

if NO: how do you accept and process them (days and times)?

___________________________________________________________________________ ___________________________________________________________________________ ___________________________________________________________________________

9) Is 24-hour emergency lactate measurement possible in all wards (point of care and/or central laboratory)?

YES NO (if partial, please specify) _______________________________________________

10) On average, how long does it take to obtain a lactate measurement result?

- in the emergency department: immediate <1hr >1h <2hr

- in inpatient area: immediate <1hr >1h <2hr >2hr

Central venous access

11) Is it possible to place a central venous catheter h24 in all in-patient wards?

YES NO

if NO, please indicate whether you require transfer to: PS or ICU YES

NO NO

operating room:

Infusion pumps

12) Is there availability of infusion pumps in all wards?

YES NO

if NO, please indicate in which wards: PS or inpatient wards

Urinometers

13) Are urinometers available in all wards?

YES NO

if NO, please indicate in which wards: PS or inpatient wards

YES

Non-invasive volemic monitoring

14) Is it possible to have non-invasive monitoring with ultrasound of the volemic status (and responsiveness) at the patient's bedside in all wards?

YES NO

if NO, please indicate in which wards: PS or inpatient wards (note)

Antibiotic therapy

15) Do you have empirical antibiotic therapy guidelines?

YES NO

if YES, is a training programme in use for sharing? YES NO

if YES, a) please indicate: date protocol was drafted: .../..../.......; date last updated: .../..../......;

b) what is the method of consulting the protocols? - paper YES NO ;

if YES, available in the department elsewhere - computer support YES NO

15a)) Do you have empirical antibiotic therapy guidelines for the obstetric patient?

a) YES NO

if YES, is a training programme in use for sharing? YES NO

if YES, a) please indicate: date protocol was drafted: .../..../.......; date last updated: .../..../......; b) what is the method for consulting the protocols?

- paper YES NO ;

if YES, available in the department elsewhere

- computer support YES NO ;

if YES, open to all with restricted access

- last updated: .....................

16) Which categories of these antibiotics do you always have available in your PS? Quinolonics YES NO , Carbapenems YES NO , Glycopeptides YES NO

Macrolides YES NO , Metronidazole YES NO , Cephalosporins III YES NO

17) Which categories of these antibiotics do you always have available in your medical wards? Quinolones YES NO , Carbapenems YES NO , Glycopeptides YES NO

Macrolides YES NO , Metronidazole YES NO , Cephalosporins III YES NO

17.1) Which categories of these antibiotics do you always have available in obstetrics?

Penicillin/beta lactamase inhibitors association YES NO , Macrolides Glycopeptides YES NO Quinolones YES NO

18) Do you have to make a reasoned request for certain antibiotics? YES NO

if YES, can you still be supplied on holidays or overnight?

Source control

19) How can the surgeon's consultation be activated?

on call active guard

How can the consultation of the Infectivologist be activated?

on call active guard

20) Is the consultant infectivologist 'routinely' contacted in cases of severe sepsis and septic shock (choice/dose Atb)?

YES NO

21) How are the surgical team and emergency room activated for source eradication?

Active guarding YES Availability YES

22) Do you have in your Hospital codified protocols shared with Surgeons aimed at eradicating the septic source?

YES NO

if YES, is a training programme in use for sharing? YES NO

if YES, a) please indicate: date protocol was drafted: .../..../.......; date last updated: .../..../......;

b) what is the method of consulting the protocols? - paper YES NO ;

if YES, available on the ward elsewhere - computer support YES NO

23) Do you have in your hospital codified and shared protocols aimed at the diagnostic and therapeutic pathway for patients with suspected meningitis?

YES NO

if YES, is a training programme in use for sharing? YES NO

if YES, a) indicate: date protocol was drafted: .../..../.......; date last updated: .../..../......;

b) what is the method of consulting the protocols? - paper YES NO ;

if YES, available in the department elsewhere

- computer support YES NO

24) Do you have in your hospital codified and shared protocols aimed at the diagnostic and therapeutic pathway of the patient with suspected central venous catheter infection?

YES NO

if YES, is a training programme for sharing in use? YES NO

if YES, a) please indicate: date protocol was drafted: .../..../.......; date last updated: .../..../......;

b) what is the method of consulting the protocols? - paper YES NO ;

if YES, available on the ward elsewhere - computer support YES NO

25) Do you have in your hospital codified and shared protocols aimed at the diagnostic and therapeutic pathway for patients with suspected community pneumonia?

YES NO

if YES, is a training programme for sharing in use? YES NO

if YES, a) please indicate: date protocol was drafted: .../..../.......; date last updated: .../..../......;

b) what is the method of consulting the protocols? - paper YES NO ;

if YES, available on the ward elsewhere - computer support YES NO

26) Does your hospital have an interventional radiology service for source eradication?

YES NO

if YES, is it easy to activate? YES if NO, do you have a referring/convenient hospital structure?

Various

27) Is radiological examination reporting available 7 days a week h24?

YES NO

if NO, please indicate time slots ____________________ if NO, please indicate if on call: YES NO

YES

NO NO

28) Is a surveillance system for infections/sepsis/septic shock in pregnancy/puperium used in your department/hospital?

a) YES NO

if YES, please specify which and frequency....................................................................

29) In your maternity unit, are the risk factors (give examples: BMI, social risk, ethnicity, smoking, lifestyle, etc.) for infection/sepsis once identified systematically reported on the obstetrical chart or other health documentation and easily identifiable by all operators during the pregnancy/puperium patient's journey?

a) YES NO

if YES, please specify how: ........................ ...................................

30) Report any difficulties encountered in the diagnostic-therapeutic pathway of the septic patient in your hospital or comments on the completion of the LO check-list: A)_________________________________________________________________________________ B)_________________________________________________________________________________ C)_________________________________________________________________________________

31) Has the Corporate Improvement Group been identified YES NO

If YES, please indicate who it is composed of (role, function) and indicate a Contact Person with NAME - SURNAME - MAIL and PHONE CONTACT) .................................................................................................................

b) .................................................................................................................

c) .................................................................................................................

d) .................................................................................................................

e) .................................................................................................................

f) .................................................................................................................

g) .................................................................................................................

h) .................................................................................................................

i) .................................................................................................................

j) .................................................................................................................

k) .................................................................................................................

l) .................................................................................................................

m) .................................................................................................................

n) .................................................................................................................

Rapporteur:

Nome..................................................... Cognome.......................................................... e.mail ............................................................................................................

contatto telefonico .............................................................................................................................................................................

# BIBLIOGRAPHY

- Acosta CD, Kurinczuk JJ, Lucas DN, Tuffnell DJ, Sellers S, Knight M; United Kingdom Obstetric Surveillance System. Severe maternal sepsis in the UK, 2011-2012: a national case-control study. PLoS Med. 2014;11:e1001672.

-  AGENAS. Linee di indirizzo clinico-organizzative per la prevenzione delle complicanze legate alla gravidanza. 2017

-  Agency for Healthcare Research and Quality (AHRQ) Patient Safety Indicators Technical Specifications Updates - Version 6.0, September. Disponibile in: http://www.qualityindicators.ahrq.gov/modules/PSI_TechSpec.aspx.

-  Albright CM, Ali TN, Lopes V, Rouse DJ, Anderson BL. The Sepsis in Obstetrics Score: a model to identify risk of morbidity from sepsis in pregnancy. Am J Obstet Gynecol. 2014;211:39.e1-8.

-  Albright CM, Has P, Rouse DJ, Hughes BL. Internal validation of the sepsis in obstetrics score to identify risk of morbidity from sepsis in pregnancy. Obstet Gynecol 2017; 130: 747-755.

-  Angus DC and van der Poll T. Severe Sepsis and Septic Shock. N Engl J Med 2013; 369: 840-851

-  Angus DC, Linde-Zwirbe WT, Lidicker J et Al. Epidemiology of severe sepsis in United States: Analysis of

incidence, outcome, and associated costs of care. Crit Care Me 2001; 9: 1303-1310

-  Bagshaw SM, Lapinsky S, Dial S, et al; Cooperative Antimicrobial Therapy of Septic Shock (CATSS) Database Research Group: Acute kidney injury in septic shock: clinical outcomes and impact of duration of hypotension prior to initiation of antimicrobial therapy. Intensive Care Med 2009; 35:871–881.

-  Barton JR, Sibai BM. Severe sepsis and septic shock in pregnancy. Obstet Gynecol. 2012;12:689-706.

-  Bates SM, Greer IA, Middeldorp S, Veenstra DL, Prabulos AM, Vandvik PO. VTE, thrombophilia, antithrombotic therapy, and pregnancy: Antithrombotic Therapy and Prevention of Thrombosis, 9th ed: American College of Chest Physicians Evidence-Based Clinical Practice Guidelines. Chest. 2012;141:e691S- e736S.

-  Bauer ME, Bateman BT, Bauer ST et Al. Maternal Sepsis Mortality and Morbidity During Hospitalization for Delivery: Temporal Trends and Indipendent Associations for Severe Sepsis. Anesth Analg 2013; 117: 944-50.

-  Berni G, Francois C, Tonelli L. National Early Warning Score (NEWS) Misurazione standardizzata della gravità della malattia. Linea guida Consiglio Sanitario Regionale Regione Toscana. Traduzione e adattamento dalla linea guida originale della Royal College of Physicians. Aggiornamento 2016.

-  Bonet M, Nogueira Pileggi V, Rijken MJ, Coomarasamy A, Lissauer D, Souza JP,G√olmezoglu AM. Towards a consensus definition of maternal sepsis: results of a systematic review and expert consultation. Reprod Health. 2017;14:67.

-  Bowyer L, Robinson HL, Barrett H, et al.: SOMANZ guidelines for the investigation and management sepsis in pregnancy.Aust NZ Obstet Gynaecol 2017;57:540-551

-  Bryan CS, Reynolds KL, Moore EE. Bacteremia in obstetrics and gynecology. Obstet Gynecol 1984;64:155– 8.

-  Buppasiri P, Lumbiganon P, Thinkhamrop J, Thinkhamrop B. Antibiotic prophylaxis for third- and fourth- degree perineal tear during vaginal birth. Cochrane Database Syst Rev. 2014:7;(10):CD005125.

-  Cantwell R, Clutton-Brock T, Cooper G, et al. Saving Mothers' Lives: Reviewing maternal deaths to make motherhood safer: 2006-2008. The Eighth Report of the Confidential Enquiries into Maternal Deaths in the United Kingdom. BJOG. 2011;118 Suppl 1:1-203.

-  CEMACH 2007. The Seventh Report of the Confidential Enquiries into Maternal Deaths in the United Kingdom.

-  Chongsomchai C, Lumbiganon P, Laopaiboon M. Prophylactic antibiotics for manual removal of retained placenta in vaginal birth. Cochrane Database Syst Rev. 2014:20;(10):CD004904.

-  Cole MF. A modified early obstetric warning system. BJ of Midwifwery 2014;22: 862-68

-  Cordioli RL, Cordioli E, Negrini R, Silva E. Sepsis and pregnancy: do we know how to treat this situation?

Rev Bras Ter Intensiva. 2013;25:334-44.

-  Creanga AA, Syverson C, Seed K, Callaghan WM. Pregnancy-Related Mortality in the United States, 2011- 2013. Obstet Gynecol. 2017;130:366-373.

-  Daniels et al. The sepsis six and the severe sepsis resuscitation bundle: a prospective observational cohort study. Emerg Med J 2011 28: 507-512.

-  Dellinger RP, Levy MM, Rhodes A, et al. Surviving Sepsis Campaign: International guidelines for management of severe sepsis and septic shock: 2012. Crit Care Med 2013; 41:580–637.

-  Donati S, Senatore S, Ronconi A. Maternal mortality in Italy: a record-linkage study. BJOG. 2011;118:872- 9.

-  Drees M, Gerber JS, Morgan Dj. Research Methods in Healthcare Epidemiology and Antimicrobial Stewardship: Use of Administrative and Surveilance Databases. Infect Control Hosp Epidemiol 2016: 1-10.

-  Drugs and Lactation Database (LactMed). Disponibile in: https://toxnet.nlm.nih.gov/newtoxnet/lactmed.htm.

-  European Centre for Disease Prevention and Control. Antimicrobial resistance surveillance in Europe 2015. Annual Report of the European Antimicrobial Resistance Surveillance Network (EARS-Net). Stockholm: ECDC; 2017.

-  Fernandez-Perez ER, Salman S, Pendem S, Farmer JC. Sepsis during pregnancy. Crit Care Med 2005;33:S286–93.

-  Ferrer R, Martin-Loeches I, Phillips G, et al: Empiric antibiotic treatment reduces mortality in severe sepsis and septic shock from the first hour: results from a guideline-based performance improvement program. Crit Care Med 2014; 42:1749–1755

-  Ford JM, Scholefield H. Sepsis in obstetrics: cause, prevention, and treatment. Curr Opin Anaesthesiol. 2014;27:253-8.

-  Geller SE, Rosenberg D, Cox SM, Brown LM, Simonsons L, Driscoll CA, Kilpatrick SJ. The continuum of maternal morbidity and mortality: Factors associated with severity. Am J Obstet Gynecol 2004, 191: 939- 44.

-  Giani T, Pini B, Arena F, Conte V, Bracco S, Migliavacca R; AMCLI-CRE Survey Participants, Pantosti A, Pagani L, Luzzaro F, Rossolini GM. Epidemic diffusion of KPC carbapenemase-producing Klebsiella pneumoniae in Italy: results of the first countrywide survey, 15 May to 30 June 2011. Euro Surveill. 2013;18.

-  Government of South Australia. Perinatal practice guidelines, Clinuical Guidelines, Sepsis in Pregnancy 2017, CG 190.

-  Henderson E, Love EJ. Incidence of hospital-acquired infections associate with caesarean section. J Hosp Infect. 1995;29:245-55.

70

-  Hussein J, Mavalankar DV, Sharma S, D'Ambruoso L. A review of health system infection control measures in developing countries: what can be learned to reducematernal mortality. Global Health. 2011;7:14.

-  Intrapartum Fetal Surveillance Clinical Guidelines. RANZCOG 2014. Disponibile
in: https://www.ranzcog.edu.au/intrapartum-fetal-surveillance-clinical-guidelines.html.

-  Joseph J, Sinha A, Paech M, Walters BNJ. Sepsis in pregnancy and early goal-directed therapy. Obstetric Medicine. 2009;2:93-99.

-  Kourtis AP, Read JS, Jamieson DJ. Pregnancy and infection. N Engl J Med. 2014;370:2211-8.

-  Knowles SJ, O'Sullivan NP, Meenan AM, Hanniffy R, Robson M. Maternal sepsis incidence, aetiology and

outcome for mother and fetus: a prospective study. BJOG.2015;122:663-71.

-  Kumar A, Roberts D, Wood KE, et al: Duration of hypotension before initiation of effective antimicrobial therapy is the critical determinant of survival in human septic shock. Crit Care Med 2006; 34:1589–1596

-  Mabie WC, Barton JR, Sibai B. Septic shock in pregnancy. Obstet Gynecol 1997;90:553–61.

-  Martin GS, Mannino DM, Eaton S, Moss M. The epidemiology of sepsis in the United States from 1979

through 2000. N Engl J Med 2003; 348:1546–54.

-  Micek ST, Welch EC, Khan J, Pervez M, Doherty JA, Reichley RM, Kollef MH. Empiric combination antibiotic therapy is associated with improved outcome against sepsis due to Gram-negative bacteria: a retrospective analysis. Antimicrob Agents Chemother. 2010;54:1742-8.

-  Ministero della Salute. Near miss ostetrici in Italia: la sepsi, l’eclampsia, l’embolia di liquido amniotico e l’emoperitoneo spontaneo - Progetto finanziato dal programma CCM 2016 del Ministero della Salute.

-  Morgan J, Roberts S. Maternal Sepsis. Obstet Gynecol Clin North Am. 2013; 40 (1):69-87.

-  National Clinical Effectiveness Committee (NCEC). Sepsis Management. National Clinical Guideline No. 6.

Disponibile in: www.health.gov.ie/patientsafety/ncec www.hse.ie/sepsis

-  National Institute for Health and Clinical Excellence. Acutely ill patients in hospital. Recognition of and

response to acute illness in adults in hospital. NICE clinical guideline 50. London: NICE, 2007.

-  National Institute for Health and Clinical Excellence. Pregnancy and complex social factors: a model for service provision for pregnant women with complex social factors, NICE Guideline 2010, updated 2018.

-  Olvera L, Dutra D. Early recognition and management of maternal sepsis. Nurs Womens Health 2016;2:182-95.

-  Parfitt SE, Bogat ML, Roth C. Sepsis in obstetrics: treatment, prognosis and prevention. Am J Matern Child Nurs 2017: 42:206-209.

-  Patterson C et al. Early warning systems in the UK: variation in content and implementation strategy has implications for a NHS early warning system. Clin Med 2011; 11: 424-7.

-  Plante LA. Management of sepsis and septic shock for the obstetrician-gynecologist. Obstet Gynecol Clin North Am. 2016; 43:659-678.

-  Report of the National High Blood Pressare Education Program. Working group report in high blood pressure in pregnancy. Am J Obstet Gynecol 2000, 183: S1-22.

-  Rhodes et al. Surviving Sepsis Campaign: International Guidelines for Management of Sepsis and Septic Shock 2016. Critical Care Medicine, 2017 ;45: 486–552.

-  Robson WP, Daniel R. The Sepsis Six: helping patients to survive sepsis. Br J Nurs. 2008;17:16-21.

-  Royal College of Obstetricians & Gynaecologist. Bacterial Sepsis following Pregnancy. 2012 Green-top Guideline No.64b.

-  Royal College of Physician. National Early Warning Score (NEWS) Standardising the assessment of acute- illness severity in the NHS. Report of a working party July 2012.

-  Shields LE, Wiesner S, Klein C., Pelletrea B, Hedrian HL. Use of maternal early warning trigger tool reduces maternal morbidity. American Journal of Obstetrics and Gynecology, 214(4), 527.

-  Singer M, Deutschman CS, Seymour CW. The Third International Consensus Definitions for Sepsis and Septic Shock (Sepsis-3). JAMA 2016; 315: 801-810.

-  Smaill, FM, Gyte GM. Antibiotic prophylaxis versus no prophylaxis for preventing infection after cesarean section. Cochrane Database Syst Rev, 2010(1): p. CD007482.

-  World Health Organization, UNICEF, UNFPA, The World Bank. Maternal Mortality 1990 to 2008: Estimates Developed by WHO, UNICEF, UNFPA and The World Bank. Geneva: WHO, 2010.

-  World Health Organization, UNICEF, UNFPA, The World Bank. Maternal Mortality 1990 to 2008: Estimates Developed by WHO, UNICEF, UNFPA and The World Bank. Geneva: WHO, 2010.

-  World Health Organization. Statement on Maternal Sepsis, 2017. Disponibile in: http://www.who.int/reproductivehealth/publications/maternal_perinatal_health/maternalsepsis- statement/en/

-  Zaidi AK, Huskins WC, Thaver D, Bhutta ZA, Abbas Z, Goldmann DA. Hospital-acquired neonatal infections in developing countries. Lancet. 2005;365:1175-88.
