# Supplementary material for: A Multicenter Study in Northern Italy to Evaluate the Impact of a Sepsis Bundle in Obstetric Settings: The SOS Study
Source: Open Forum Infect Dis. 2025 Jun 16;12(7):ofaf337. doi: 10.1093/ofid/ofaf337 (PMC12216898; doi:10.1093/ofid/ofaf337)
Supplement: ofaf337_Supplementary_Data [file ofaf337_supplementary_data.zip › SOS supplements.docx]

**A multi-center study in Northern Italy to evaluate the impact of *Sepsis bundle* in**
**Obstetric Settings: the SOS study**

**SUPPLEMENTARY MATERIAL
Figure S1** Modified Early Obstetric Warning Score (MEOWS)
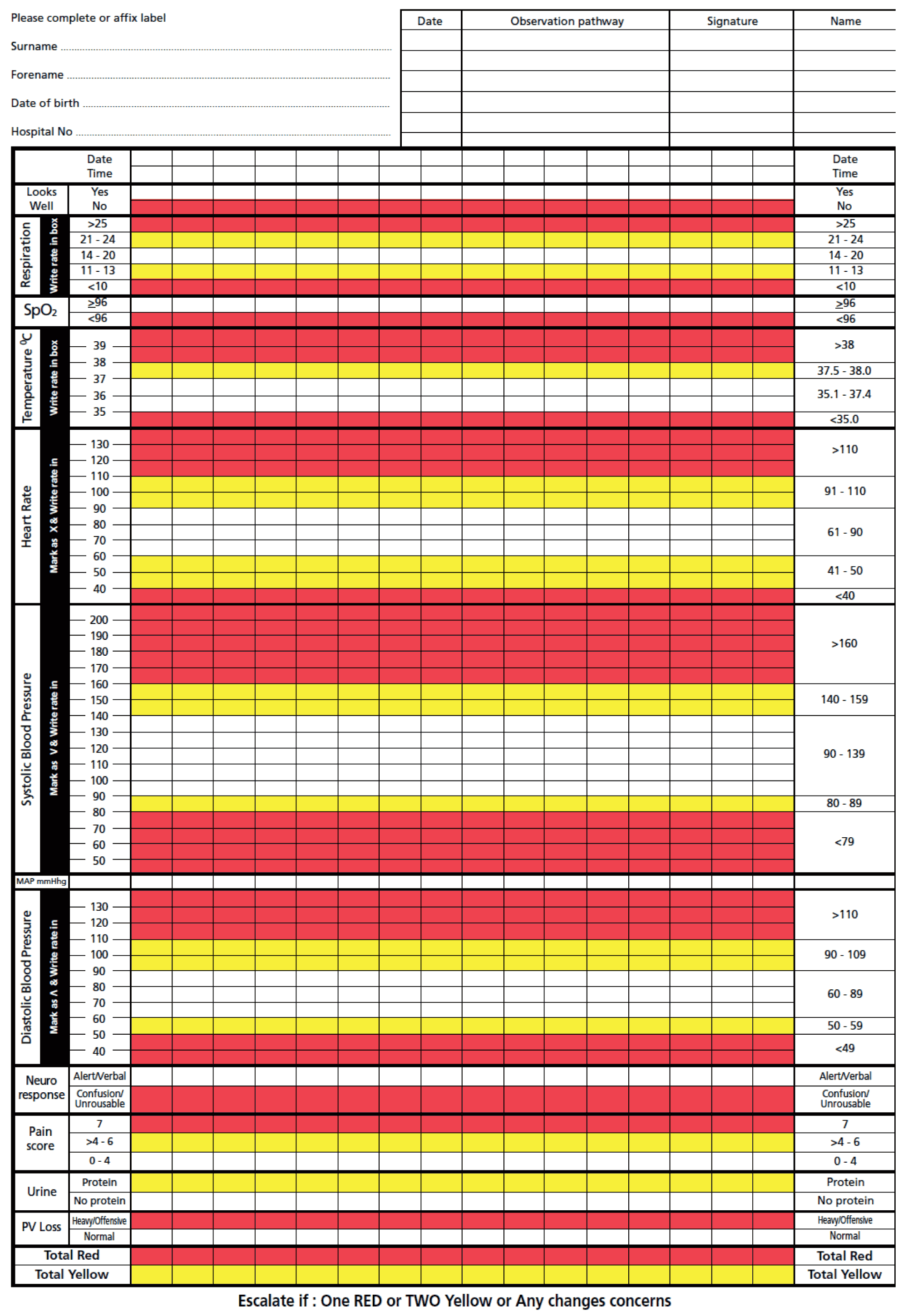


**Table S1** Microbial isolates in urine, foetus/newborn cultures placenta and vaginal/cervical swab.

|  | **N** | **Overall**, N=80^1^ | **Pre-budle**, N=24^1^ | **Post-budle**, N=56^1^ | **p-value**^2^ |
| --- | --- | --- | --- | --- | --- |
| **Urine cultures performed** |  | 57(71.3%) | 15(62.5%) | 42 (75.0%) | 0.6 |
| **Positive urine cultures** | 57 | 26(45.6%) | 6(40.0%) | 20(47.6%) | 0.6 |
| **Isolated pathogens** | 23 |  |  |  | 0.3 |
| *Escherichia coli^3^* |  | 15(65.2%) | 4(100.0%) | 11(57.9%) |  |
| *Enterococcus spp.^4^* |  | 4(17.4%) | 0(0.0%) | 4(21.1%) |  |
| *Klebsiella pneumoniae* |  | 2(8.7%) | 0(0.0%) | 2(10.5%) |  |
| *Enterobacter aerogenes* |  | 1(4.3%) | 0(0.0%) | 1(5.3%) |  |
| *Pseudomonas aeruginosa* |  | 1(4.3%) | 0(0.0%) | 1(5.3%) |  |
| **Fetus or Newborn Cultures performed** |  | 3(3.8%) | 2 (8.3%) | 1 (1.8%) | >0.9 |
| **Fetus or Newborn Cultures positive** | 3 | 3(100.0%) | 2(100.0%) | 1(100.0%) | na |
| **Isolated pathogens** | 4 |  |  |  | >0.9 |
| *Escherichia coli* |  | 1 (25.0%) | 0 (0.0%) | 1(50.0%) |  |
| *Klebsiella pneumoniae* |  | 2 (50.0%) | 1(50.0%) | 1(50.0%) |  |
| *Listeria monocytogenes* |  | 1 (25.0%) | 1(50.0%) | 0(0.0%) |  |
| **Placenta Cultures performed** |  | 9(3.8%) | 2(8.3%) | 7 (1.8%) | 0.5 |
| **Placenta Cultures positive** | 9 | 6(67%) | 2(100.0%) | 4 (57.1%) | 0.5 |
| **Isolated pathogens** | 7 |  |  |  | 0.6 |
| *Escherichia coli* |  | 3(42.8%) | 1 (50.0%) | 2 (40.0%) |  |
| *Klebsiella pneumoniae* |  | 2(28.6%) | 0 (0.0%) | 2 (40.0%) |  |
| *Staphylococcus epidermidis^5^* |  | 1(14.3%) | 1 (50.0%) | 0 (0%) |  |
| *Enterococcus faecalis* |  | 1(14.3%) | 0(0.0%) | 1 (25.0%) |  |
| **Vaginal/cervical Swab Cultures performed** |  | 40(50.0%) | 14(58.3%) | 26(46.4%) | 0.6 |
| **Vaginal/cervical Swab positive** | 40 | 14(35.0%) | 7 (50.0%) | 7 (26.9%) | 0.2 |
| **Isolated pathogens** | 17 |  |  |  | 0.3 |
| *Candida spp* |  | 4(23.5%) | 3(37.5%) | 1(11.1%) |  |
| *Escherichia coli* |  | 4(23.5%) | 2(25.0%) | 2(22.2%) |  |
| *Streptococcus spp.* |  | 4(23.5%) | 3(37.5%) | 1(11.1%) |  |
| *Gardnerella vaginalis* |  | 1(5.9%) | 0 (0%) | 1(11.1%) |  |
| *Klebsiella pneumoniae* |  | 1(5.9%) | 0 (0%) | 1(11.1%) |  |
| *Ureaplasma spp.* |  | 1(5.9%) | 0 (0%) | 1(11.1%) |  |
| *Citrobacter koseri* |  | 1(5.9%) | 0 (0%) | 1(11.1%) |  |
| *Prevotella bivia* |  | 1(5.9%) | 0 (0%) | 1(11.1%) |  |

*^1^* n (%)

*^2^* Pearson’s Chi-squared test and Fisher’s exact test

*^3^ 1/15 was an Extended-spectrum beta-lactamases producing E.coli.*

*^4^ Enterococcus spp includes 3/4 (75%) Enterococcus faecalis and 1/4 (25%) Enterococcus faecium*

*^5^ Methicillin Resistant Staphylococcus epidermidis (MRSE)*

*No Methicillin Resistant Staphylococcus aureus, Vancomycin-Resistant E.faecium, AmpC/Carbapenemases producing Enterobacterales have been found*
