# Supplementary material for: A Multicenter Study in Northern Italy to Evaluate the Impact of a Sepsis Bundle in Obstetric Settings: The SOS Study
Source: Open Forum Infect Dis. 2025 Jun 16;12(7):ofaf337. doi: 10.1093/ofid/ofaf337 (PMC12216898; doi:10.1093/ofid/ofaf337)
Supplement: ofaf337_Supplementary_Data [file ofaf337_supplementary_data.zip › Operational Guidance Document_7691.pdf]

RegioneLombardia

DECRETO N. 7691 Del 28/05/2018

Identificativo Atto n. 287

DIREZIONE GENERALE WELFARE

Oggetto:

APPROVAZIONE DEL DOCUMENTO DI INDIRIZZO OPERATIVO PER L'IDENTIFICAZIONE PRECOCE E LA GESTIONE DELLA SEPSI IN OSTETRICIA

L'atto si compone di ________ pagine

di cui _______ pagine di allegati

parte integrante

RegioneLombardia

IL DIRETTORE GENERALE

PREMESSO che:

- la sepsi materna, è una condizione pericolosa per la vita definita come disfunzione d'organo causata da un'infezione insorta in gravidanza, durante il parto, a seguito di aborto o nel periodo post-natale;
- rappresenta una condizione clinica di difficile gestione associata a una mortalità elevata in particolare se complicata da uno stato di shock;
- nel nostro Paese la sepsi risulta la seconda causa di morte materna diretta, dopo l'emorragia ostetrica, con una percentuale pari al 10%;
- in Lombardia, secondo un'analisi delle SDO, l'incidenza della sepsi materna è pari allo 0,33/1000 parti/anno (analisi effettuata su 49.984 parti dal 2009 al 2013);
- malgrado si sia assistito negli ultimi anni ad una riduzione delle morti materne legate ad infezioni e sepsi, la sepsi materna rappresenta a tutt'oggi, una delle principali cause prevenibile di morte;

RICHIAMATI i seguenti provvedimenti regionali:

- il decreto DG Sanità (ora Welfare) n. 7846 del 29/07/2009 "*Prevenzione e controllo delle infezioni ospedaliere e correlate all'assistenza sanitaria – indicazioni per l'organizzazione e rendicontazione delle attività di indirizzo della Direzione Generale Sanità e delle strutture sanitarie accreditate di ricovero e cura*", che, tra gli altri, ha previsto l'attivazione di Gruppi di Approfondimento Tecnico (GAT) su specifiche tematiche, fra le quali anche la gestione dei casi di sepsi severa;
- il decreto DG Salute n. 7517 del 5/08/2013 che ha approvato il documento tecnico "*Strategie integrate per ridurre la mortalità ospedaliera associata alla sepsi grave*" dove sono indicati i percorsi e le attività per la gestione degli interventi nei casi di sepsi;

RICHIAMATA la d.g.r. n. X/7600 del 20/12/2017 "*Determinazioni in ordine alla*

gestione del servizio sociosanitario per l'esercizio 2018" che tra i temi ritenuti centrali e prioritari nel 2018 per l'attuazione dei progetti aziendali di Risk management, riconferma l'attenzione al contesto del travaglio/parto;

CONSIDERATO che nella popolazione ostetrica, i fattori chiave in grado di migliorare significativamente la prognosi sono il riconoscimento rapido e la gestione tempestiva della sepsi e che ciò si può attuare grazie ad un approccio clinico-organizzativo del percorso assistenziale;

ATTESO che la DG Welfare si avvale dei professionisti esperti del sistema sociosanitario regionale in base alle loro specifiche competenze cliniche, scientifiche e alla consolidata esperienza professionale, al fine di elaborare, condividere documenti e linee guida per assicurare appropriatezza, qualità e sicurezza diagnostico-terapeutica dei percorsi di presa in carico;

DATO ATTO che allo scopo presso la DG Welfare, è stato attivato un tavolo di lavoro multidisciplinare composto da Medici Ginecologi, Anestesisti-Rianimatori, Ostetriche e Risk Manager, con l'obiettivo di proporre, secondo gli indirizzi regionali di Risk Management, un documento operativo rivolto a tutte le strutture regionali per l'identificazione precoce e la gestione della sepsi in ostetricia;

VISTO il "*Documento di indirizzo operativo per l'identificazione precoce e la gestione della sepsi in ostetricia"* elaborato dal gruppo multidisciplinare di esperti in questione:

CONSIDERATO che il documento di cui trattasi contiene raccomandazioni che si pongono quale riferimento per la pratica clinica in una logica tempo-dipendente, multiprofessionale e multidisciplinare;

RITENUTO di approvare il "*Documento di indirizzo operativo per l'identificazione precoce e la gestione della sepsi in ostetricia*" - Allegato parte integrante del presente provvedimento;

RITENUTO che le raccomandazioni contenute nel documento in argomento si intendono rivolte a tutte le strutture sanitarie del territorio lombardo che al loro

interno sono provviste di servizi di Ginecologia e Ostetricia e a quelle strutture sanitarie che pur essendone prive, si dovessero comunque trovare a gestire problematiche inerenti la gravidanza;

RITENUTO di dare mandato alle ATS di disporre la massima diffusione del documento approvato con il presente provvedimento presso le strutture sanitarie pubbliche e private afferenti al proprio territorio di competenza;

RITENUTO di disporre la pubblicazione del documento sul Portale di Regione Lombardia, www.regione.lombardia.it;

VISTI:

- la l.r. n. 20/2008, nonché i provvedimenti organizzativi della XI legislatura;
- la l.r. n. 23/2015 "*Evoluzione del sistema sociosanitario lombardo: modifiche al Titolo I e al Titolo II della legge regionale 30 dicembre 2009 n. 33 (Testo Unico delle leggi regionali in materia di sanità)";*
- il *Piano Socio Sanitario Regionale (PSSR) 2010-2014* - approvato con d.c.r. n. IX/88 del 17/11/2010 - la cui validità è stata prorogata dalla d.g.r. n. X/2989 del 23/12/2014 fino all'approvazione di un nuovo Piano;

DECRETA

1. **Di approvare** il "*Documento di indirizzo operativo per l'identificazione precoce e la gestione della sepsi in ostetricia*" - Allegato parte integrante del presente provvedimento.

2. **Di stabilire** che le raccomandazioni contenute nel documento in argomento si intendono rivolte a tutte le strutture sanitarie del territorio lombardo che al loro interno sono provviste di servizi di Ginecologia e Ostetricia e a quelle strutture sanitarie che pur essendone prive, si dovessero comunque trovare a gestire problematiche inerenti la gravidanza.

3. **Di dare mandato** alle ATS di predisporre la massima diffusione del documento di cui al punto 1, presso le strutture sanitarie pubbliche e private

afferenti al proprio territorio di competenza.

4. **Di disporre** la pubblicazione del documento sul Portale di Regione Lombardia, www.regione.lombardia.it.

IL DIRETTORE GENERALE
GIOVANNI DAVERIO

Atto firmato digitalmente ai sensi delle vigenti disposizioni di legge

Documento di indirizzo operativo per l'identificazione precoce e la gestione della sepsi in ostetricia

A seguito del Decreto Regionale n. 7517 del 5/8/2013 "Strategie integrate per ridurre la mortalità ospedaliera associata a Sepsi grave", e della "Raccomandazione per l'identificazione, la gestione e il trattamento della sepsi in età pediatrica" del 15/1/2014 il Gruppo di Lavoro "Lotta alla Sepsi in Ostetricia" di Regione Lombardia ha elaborato, secondo le indicazioni Regionali di Risk Management, un documento con l'obiettivo di proporre le definizioni e le procedure d'identificazione e gestione della sepsi nella popolazione ostetrica.

Obiettivi specifici:

1) Proporre degli elementi clinico-organizzativi per definire un percorso diagnostico-terapeutico-assistenziale in grado di garantire l'applicazione di raccomandazioni e strategie di provata efficacia per ridurre in modo significativo la morbilità e la mortalità associate alla sepsi materna in tutti i punti nascita;
2) proporre le azioni per l'applicazione delle strategie descritte.

Gli strumenti applicativi qui descritti (strumenti di prevenzione, criteri di diagnosi, strumenti d'identificazione e monitoraggio, indicazioni terapeutiche ed indicatori di struttura) rappresentano una proposta operativa per facilitarne l'implementazione e l'adattamento nelle singole strutture sanitarie.

TAVOLO DI APPROFONDIMENTO TECNICO

Moscheni Maristella	Risk Manager	ASST Nord Milano	Coordinatore Tavolo Regionale
Burato Enrico	Risk Manager	ASST Mantova	
Comberti Enrico	Risk Manager	ASST Spedali Civili Brescia	
Cetin Irene	Ostetrico Ginecologo	ASST FBF-Sacco	Coordinatore Progetti Regionali/ISS
Antonazzo Patrizio	Ostetrico Ginecologo	ASST FBF-Sacco	
Cromi Antonella	Ostetrico Ginecologo	ASST Sette Laghi	
Ferrazzi Enrico	Ostetrico Ginecologo	IRCCS CA' Granda Osp. Maggiore Policlinico	
Ghezzi Fabio	Ostetrico Ginecologo	ASST Sette Laghi	
Locatelli Anna	Ostetrico Ginecologo	ASST Vimercate	
Lojacono Andrea	Ostetrico Ginecologo	ASST Spedali Civili Brescia	
Maini Isabella	Ostetrico Ginecologo	ASST Monza	
Muggiasca Maria Luisa	Ostetrico Ginecologo	ASST FBF-Sacco	
Vergani Patrizia	Ostetrico Ginecologo	ASST Monza	
Colciago Elisabetta	Ostetrica	Università Milano Bicocca	
Del Bo Elsa	Ostetrica	IRCCS Polic. San Matteo Pavia	
Miglietta Marinella	Ostetrica	Osp. "Sacra Famiglia" FBF Erba	
Nelli Elisabetta	Ostetrica	ASST Lecco	
Monti Gianpaola	Anestesista	ASST Grande Ospedale Metropolitano Niguarda	Coordinatore Tecnico Scientifico
D'andrea Luca	Anestesista	ASST Monza	
Moise Gabriella	Anestesista	ASST Nord Milano	
Porro Giuliana	Anestesista	IRCCS CA' Granda Osp. Maggiore Policlinico	

Bersani Maurizio	Dirigente Regione Lombardia
Mozzanica Davide	Dirigente Regione Lombardia
Picchetti Chiara	Segreteria RM Regione Lombardia

Consulenze:
Dalla Gasperina Daniela - Malattie *infettive* e Tropicali, ASST Sette Laghi, Varese
Grossi Paolo Antonio - Malattie *infettive* e Tropicali, ASST Sette Laghi, Varese

SOMMARIO

Sommario delle raccomandazioni ... 5
1. Prevenzione ... 5
2. Sistemi di allerta e sepsi materna: identificazione e monitoraggio ... 5
3. Nuove definizioni della sepsi materna e identificazione del rischio ... 6
4. Primi interventi diagnostico-terapeutici per la sepsi materna ... 7

Premessa ... 10

1. La PREVENZIONE e i FATTORI di RISCHIO ... 11
Razionale ... 11
FATTORI DI RISCHIO ... 13
MISURE PREVENTIVE ... 13
PREVENZIONE IN AMBIENTE OSPEDALIERO ... 13
PREVENZIONE IN GRAVIDANZA ... 15
PROFILASSI ANTIBIOTICA ... 15
Raccomandazioni ... 16
Azioni ... 16

2. SISTEMI DI ALLERTA E SEPSI MATERNA: IDENTIFICAZIONE E MONITORAGGIO ... 17
Razionale ... 17
La scheda MEOWS: il suo uso e la risposta clinica ... 18
Raccomandazioni ... 20
Azioni ... 21
Intensificazione di cura ... 21
Raccomandazioni ... 22
Azioni ... 24
Utilizzo dei MEOWS nell'identificazione e nel monitoraggio della paziente potenzialmente settica ... 24
Raccomandazioni ... 25
Azione ... 25
Razionale ... 26
Raccomandazioni ... 26
Azioni ... 26

3. Nuove definizioni della sepsi materna e IDENTificazione del rischio ... 27
Razionale ... 27
DEFINIZIONI ... 27
COME IDENTIFICARE E FARE DIAGNOSI PRECOCEMENTE DI SEPSI/SHOCK SETTICO ... 30
IDENTIFICAZIONE del rischio ... 34
RISCHIO BASSO (MEOWS 1 parametro giallo e sospetta o certa infezione) ... 34
RISCHIO INTERMEDIO (MEOWS 2 gialli o 1 rosso e sospetta o certa infezione) ... 34
RISCHIO ALTO (MEOWS>2 gialli o >1 rosso O ≥1 CRITERIO DI DANNO D'ORGANO o lattati >2 in presenza di sospetta o certa infezione) ... 35
Raccomandazioni ... 36
Azioni ... 37

4. PRIMI INTERVENTI DIAGNOSTICO-TERAPEUTICI PER LA SEPSI MATERNA ... 37
Razionale ... 37
Raccomandazioni ... 38
Azione ... 39
PRELIEVO PER EMOCOLTURA ed ESAMI COLTURALI ... 39
Razionale ... 39
Raccomandazioni ... 40
Azioni ... 40
LATTATI, EMOGLOBINA E PARAMETRI DI FUNZIONALITA' D'ORGANO ... 40
Razionale ... 40
Raccomandazioni ... 41

Azioni **41**
MONITORAGGIO DIURESI 41
Razionale **41**
Raccomandazioni **42**
OSSIGENOTERAPIA 42
Razionale **42**
Raccomandazioni **42**
Azione **42**
TERAPIA ANTIBIOTICA 43
Razionale **43**
Raccomandazioni **45**
Azioni **45**
CONTROLLO DEL FOCOLAIO SETTICO 45
Razionale **45**
Raccomandazioni **46**
Azioni **46**
RESUSCITAZIONE VOLEMICA 46
Razionale **46**
Raccomandazioni **47**
Azioni **48**
PROFILASSI DEL TROMBOEMBOLISMO VENOSO 48
Razionale **48**
Raccomandazioni **48**
Azione **48**
ALLEGATI 49
Allegato 1. I parametri delle schede MEOWS 49
Frequenza respiratoria 49
Raccomandazione **49**
Saturazione di Ossigeno 50
Raccomandazioni **50**
Temperatura 51
Raccomandazioni **51**
Frequenza cardiaca 51
Raccomandazioni **51**
Pressione arteriosa 52
Raccomandazioni **52**
Diuresi 52
Raccomandazioni **53**
Valutazione dello stato neurologico – scala AVPU 53
Raccomandazioni **53**
Valutazione del dolore 53
Raccomandazione **54**
Allegato 2. Intensificazione di monitoraggio 55
Allegato 3. Implementazione e Ostacoli all'utilizzo della scheda MEOWS 56
Allegato 4. Scelta del regime antibiotico, rimodulazione e durata del trattamento 58
ALLEGATO 5. Check-list logistico organizzativa 63
BIBLIOGRAFIA 69

SOMMARIO DELLE RACCOMANDAZIONI

1. PREVENZIONE

- **Attuare nella popolazione ostetrica misure preventive d'infezione/sepsi la cui efficacia è comprovata da solide prove di evidenza.**

  La prevenzione si attua principalmente attraverso l'identificazione dei fattori di rischio, il buon uso di pratiche clinico-assistenziali mirate al controllo delle infezioni (es. metodiche e dispositivi appropriati per l'igiene e la disinfezione, protocolli di profilassi antibiotica) e l'educazione sanitaria alla popolazione ostetrica.
- **Utilizzare sistemi di monitoraggio e allerta (Modified Early Obstetric Warning Score, MEOWS) come strumento per una precoce identificazione di infezione/sepsi nella popolazione ostetrica.**
- **Riconoscere e riportare i fattori di rischio nella documentazione sanitaria in modo ben visibile ad ogni accesso ospedaliero e/o durante il ricovero della paziente ostetrica.**
- **Programmare la formazione del personale sanitario sui fattori di rischio e sulle misure preventive d'infezione/sepsi nella popolazione ostetrica.**

2. Sistemi di allerta e sepsi materna: identificazione e monitoraggio

- **In tutte le donne in gravidanza o entro 42 giorni dal suo esito giunte al punto nascita (Pronto Soccorso ostetrico o generico) o già ricoverate, la rilevazione e il monitoraggio dei parametri vitali deve essere effettuato utilizzando uno score di allerta precoce specifico per la popolazione ostetrica (MEOWS).**
- **Nella donna in travaglio, dal momento dell'accesso in sala parto, la rilevazione dei parametri vitali, che segue il protocollo locale, deve essere invece riportata nel partogramma.**
- **Nella donna all'uscita dalla sala parto o dalla camera operatoria l'ultima rilevazione dei parametri vitali deve essere riportata sulla scheda MEOWS prima del trasferimento della paziente in reparto.**
- **La frequenza di rilevazione dei parametri del MEOWS è determinata dal rischio evolutivo, dalla diagnosi d'ingresso e/o complicanza clinica e dall'ultima valutazione (punteggio codice colore); il livello di allerta del MEOWS è determinato unicamente dalla valutazione (punteggio del codice colore).**
- **In caso di rilevazione di ≥1 parametro giallo MEOWS considerare ed escludere il sospetto di infezione/sepsi/shock settico ricercando segni/sintomi di un'eventuale infezione e di un eventuale danno d'organo.**
- **In caso di rapido deterioramento del quadro clinico, predisporre un percorso di "intensificazione di cura".**
- **In caso di rapido deterioramento del quadro clinico, nel caso in cui non sia possibile una valutazione immediata da parte del medico di guardia, considerare il coinvolgimento di un altro Medico Ginecologo-Ostetrico e/o Anestesista-Rianimatore più esperto o coinvolgere precocemente il Direttore / Primario. L'impossibilità di una valutazione immediata da parte del medico di guardia**

è una precisa indicazione alla chiamata del Medico Reperibile.

- In caso di rapido deterioramento del quadro clinico, valutare l'ipotesi di un trasferimento della paziente in una struttura più idonea, se localmente non è possibile garantire l'intensificazione del percorso di cura. Ove il trasferimento non fosse possibile, NON si deve ritardare l'esecuzione degli interventi diagnostico-terapeutici indicati (es. esami di laboratorio, ECG, EGA o Rx torace).
- In caso di rapido deterioramento del quadro clinico:
  - effettuare la sequenza di valutazione ABCDE *(Airway, Breathing, Circulation, Delivery, Esecuzione del piano di cura);*
  - effettuare una rivalutazione continua delle condizioni cliniche della paziente, inclusi la sequenza ABCDE e l'esame obbiettivo, oltre alla revisione della documentazione clinica e la raccolta anamnestica;
  - intensificare il monitoraggio e l'assistenza alla paziente da parte dell'Ostetrica, secondo schemi prestabiliti, in attesa della valutazione da parte del Medico;
  - coinvolgere precocemente competenze multidisciplinari (Medico Ginecologo, Infettivologo, Anestesista Rianimatore).

3. NUOVE DEFINIZIONI DELLA SEPSI MATERNA E IDENTIFICAZIONE DEL RISCHIO

DEFINIZIONI

– La diagnosi di Sepsi materna prevede il riscontro, durante la gravidanza, il parto, il periodo postaborto o postpartum, di un'infezione presunta o accertata associata al danno di uno o più organi che si può evidenziare con uno o più dei seguenti criteri:
  - Necessità O2 per mantenere SpO2>95% o PaO2/FiO2 <400;
  - Valore di piastrine <100 x 10^6/L;
  - Valore di bilirubinemia > 1,2mg/dL;
  - PAS <90 mmHg o PAM<75mmHg;
  - Paziente risvegliabile su stimolo verbale, doloroso o incosciente;
  - Valore di creatininemia >1,2mg/dL.

Il riscontro di uno o più di questi criteri di danno d'organo corrisponde ad un punteggio del SOFA (Sequential Organ Failure Assessment) score modificato ≥1 .
La diagnosi di sepsi prevede pertanto il riscontro di un'infezione certa o presunta ed un punteggio del SOFA score modificato ≥1.

– La diagnosi di shock settico prevede il riscontro di un'infezione presunta o accertata associata ad ipotensione con necessità di somministrare farmaci vasopressori per mantenere una PAM ≥65mmHg e una lattacidemia >2mmol/L dopo adeguata resuscitazione volemica.

DIAGNOSI

– Per identificare l'eventuale presenza di un'infezione in atto, effettuare un'accurata anamnesi (sintomi riferiti dalla paziente) e/o un attento esame clinico (segni/sintomi rilevati dall'operatore).

– Per identificare un eventuale danno d'organo effettuare la rilevazione dei parametri vitali (MEOWS) e la misura degli esami di laboratorio (pannello sepsi).

– La diagnosi di danno d'organo si basa sul riscontro di ≥1 criteri di danno d'organo sopracitati evidenziabili da un'alterazione dei parametri vitali e/o degli esami di laboratorio (punteggio del SOFA score Modificato ≥1).

- **Nella pratica clinica è importante ricercare in tutte le pazienti con un'infezione presunta o accertata segni/sintomi di danno d'organo e in tutti pazienti con danno d'organo non altrimenti spiegabile ricercare segni/sintomi di infezione presunta o accertata per poter confermare o escludere la diagnosi di sepsi/shock settico; nei pazienti con alterazione dei parametri vitali secondo MEOWS (≥2 gialli o ≥1 rosso) è importante ricercare segni/sintomi di un'eventuale infezione e di un eventuale danno d'organo per poter confermare o escludere la diagnosi di sepsi/shock settico**.

MODALITA' OPERATIVE

- **Step 1: in tutti i casi di sospetta o certa infezione il percorso diagnostico-terapeutico assistenziale deve prevedere quanto di seguito riportato:**
  - **valutazione dell'Ostetrica e del Medico Ginecologo di guardia;**
  - **rilevazione dei parametri vitali secondo il sistema di allerta/ monitoraggio MEOWS;**
  - **valutazione fetale.**
- **Step 2: le azioni successive sono definite in base alla rilevazione o meno di un allarme di deterioramento identificabile con il codice colore MEOWS. Sul codice colore MEOWS in presenza di sospetta o certa infezione viene definito un livello di rischio con le relative modalità operative qui di seguito riportate:**
  - **RISCHIO BASSO: sospetta o accertata infezione + 1 parametro giallo MEOWS**; in tale condizione il Ginecologo valuta i parametri vitali, il benessere fetale, stabilisce l'eventuale esecuzione di esami ematochimici (*pannello sepsi) e/o esami radiologici (ricerca fonte settica) e/o l'eventuale richiesta di consulenza specialistica, procedendo al trattamento terapeutico più adeguato in base alle condizioni cliniche della paziente. Il monitoraggio dei parametri vitali prosegue secondo l'algoritmo MEOWS.
  - **RISCHIO INTERMEDIO:** sospetta o accertata infezione +2 parametri gialli o 1 rosso MEOWS; tale condizione richiede sempre:
    - la valutazione dei criteri diagnostici di danno d'organo (SOFA score modificato) e quindi l'invio degli esami ematochimici (*pannello sepsi);
    - la misurazione dei lattati sierici
    - l'effettuazione dei Sepsis Six;
    - monitoraggio dei PV secondo algoritmo MEOWS;
    - supporto delle funzioni vitali dove necessario;
    - monitoraggio fetale.
  - **RISCHIO ALTO: sospetta o accertata infezione+ >2 parametri gialli o >1 rosso MEOWS o la presenza di uno o più criteri di danno d'organo (SOFA score modificato ≥1) o lattati >2 mmol/l;** tale condizione richiede sempre tutti gli interventi del rischio intermedio e la valutazione congiunta dell'anestesista-rianimatore, ginecologo ed infettivologo (quest'ultimo ove possibile).
- **Gli esami di laboratorio (pannello sepsi) devono essere richiesti in tutti i casi di rischio intermedio e rischio alto e su valutazione medica in caso di rischio basso.**

  * Si definisce "pannello sepsi" l'insieme dei seguenti esami: emocromo completo con formula, lattati, elettroliti, azotemia, creatininemia, bilirubinemia, PT-PTT, PCR o PCT.

4. Primi interventi diagnostico-terapeutici per la sepsi materna

- **Attuare tutti gli interventi dei Sepsis Six in tutti i casi di sospetta o accertata sepsi materna o shock settico, entro la prima ora dal sospetto diagnostico.**

  Lo schema Sepsis Six prevede l'attuazione di 6 interventi, di cui: 3 diagnostici (prelievo per emocoltura, misurazione dei lattati ed altri esami di laboratorio, monitoraggio della diuresi) e 3 terapeutici (somministrazione di ossigeno, di fluidi e antibioticoterapia).

- **In particolare attuare i Sepsis Six in tutte le pazienti con:**
  - **rischio intermedio: segni/sintomi infezione + 2 gialli o 1 rosso MEOWS;**
  - **rischio elevato: segni/sintomi d'infezione +>2 gialli o >1 rosso MEOWS o mSOFA≥1;**
  - **l'attuazione dei Sepsis Six nella paziente a rischio basso è da considerarsi unicamente su indicazione medica;**
- **Prima di somministrare l'antibiotico eseguire almeno 2 set di emocolture; se la terapia antibiotica fosse già in corso, eseguire il prelievo prima della somministrazione successiva.**
- **Effettuare esami colturali per tutte le sedi anatomiche che potrebbero essere focolaio di partenza dell'infezione.**
- **Gli esami colturali appropriati devono poter essere effettuati in tutte le unità di degenza ed in Pronto Soccorso.**
- **Misurare il valore ematico dei lattati in tutte le pazienti ostetriche con segni e/o sintomi di sospetta o presunta infezione, tramite prelievo arterioso o venoso da accesso periferico o centrale mediante una metodica point of care.**
- **Misurare i parametri di funzionalità d'organo ("pannello sepsi").**
- **La trasfusione di emazie concentrate in caso di sepsi/shock settico (in assenza di altre patologie quali ischemia miocardica, ipossiemia severa o emorragia massiva) è consigliata quando il livello di Hb è < 7 g/dL.**
- **Monitorare la diuresi in tutte le pazienti ostetriche con sospetta o accertata infezione/sepsi/shock settico.**
- **Valutare e monitorare la funzione respiratoria (frequenza respiratoria e SpO2) in tutte le pazienti con sospetta o accertata sepsi.**
- **Somministrare ossigenoterapia con FiO_2 100% con maschera facciale con reservoir, dotata di valvola non–rebreathing nelle pazienti ostetriche con sospetta o accertata sepsi.**
- **Somministrare il trattamento antibiotico per via endovenosa non appena possibile e comunque entro la prima ora in tutti i casi di sospetta o accertata sepsi/shock settico e più nello specifico nelle pazienti a rischio intermedio e alto.**
- **Iniziare il trattamento empirico con uno o più antibiotici ad ampio spettro che possano coprire i patogeni più probabilmente coinvolti nell'eziologia della sepsi o dello shock settico.**

  La principale fonte settica in ambito ostetrico è il tratto genitale, seguito dalle vie urinarie e dalle ferite; da studi internazionali i microrganismi più frequentemente isolati in ambito ostetrico sono l'E.Coli, lo Streptococco di Gruppo B ed i germi anaerobi; spesso sono presenti infezioni miste (Gram positivi e negativi). La combinazione penicillina/inibitore delle beta lattamasi in associazione ad un aminoglicosìde può essere considerata come prima scelta nel trattamento della sepsi materna.

- **Una volta identificato il patogeno responsabile e ottenuto l'antibiogramma e/o documentato un miglioramento clinico, occorre modulare il trattamento antibiotico.**
- **Identificare la sede del focolaio settico e ove possibile eradicarla.**

- **In caso di eradicazione di un focolaio settico, prelevare campioni del materiale biologico infetto (liquidi biologici o biopsie tissutali) e inviarlo per esame colturale.**
- **Considerare sempre possibili focolai settici non ostetrici (es. appendicite, ascesso pancreatico, infarto intestinale, influenza).**
- **Rimuovere prontamente gli accessi vascolari ritenuti possibili focolai settici, non appena siano stati posizionati altri accessi vascolari.**
- **Valutare la vitalità fetale in tutti i casi d'ipoperfusione o di shock conclamato; valutare la decisione di espletare il parto nell'ambito della sepsi ostetrica bilanciando i rischi legati all'epoca gestazionale, le condizioni materne e quelle fetali. La stabilizzazione delle condizioni materne è prioritaria prima di espletare il parto per compromissione del benessere fetale, poiché l'intervento sulla madre potrebbe migliorare la condizione del feto.**
- **Nelle pazienti con ipotensione in caso di sospetta o accertata sepsi/shock settico, effettuare un carico volemico di 30ml/kg di cristalloidi in boli refratti di 500 ml entro la prima ora; dopo la 20° settimana di gravidanza, posizionare la paziente in decubito laterale sinistro.**
- **Effettuare la resuscitazione volemica con cristalloidi alternando soluzione fisiologica con soluzione elettrolitiche bilanciate; si suggerisce di utilizzare albumina in aggiunta ai cristalloidi nelle pazienti che abbiano necessità di elevata quantità di fluidi.**
- **Valutare la risposta alla resuscitazione volemica sia in termini di efficacia (miglioramento PA, perfusione periferica: diuresi e lattati), che in termini di complicanze.**
- **Proseguire la resuscitazione volemica fino al raggiungimento dell'obbiettivo di una PAM ≥65mmHg per una dose massima di 30ml/kg; dosi aggiuntive di fluide devono essere eventualmente prescritte dal Medico Anestesista Rianimatore.**
- **Nella sepsi materna è raccomandata la profilassi farmacologica del trombo-embolismo venoso.**

PREMESSA

La Sepsi materna, è una condizione pericolosa per la vita definita come disfunzione d'organo causata da un'infezione insorta in gravidanza, durante il parto, a seguito di aborto o nel periodo post-natale (World Health Organization, Statement on Maternal Sepsis, 2017), rappresenta una condizione clinica di difficile gestione e a tutt'oggi associata a una mortalità elevata in particolare se complicata da uno stato di shock.

La competenza immunologica in gravidanza non diminuisce ma va incontro ad una modificazione che consente all'organismo materno di adattarsi al feto. Quindi assistiamo ad una condizione immunologica modulata e non a uno stato di immunosoppressione. Infatti la riduzione della produzione di IgG, è compensata da un aumento dei leucociti. La riduzione del numero e della funzione delle cellule CD4+, CD8+ e natural killer potrebbe influenzare le risposte antivirali, antimicotiche o antiparassitarie e ritardare la clearance del microrganismo patogeno. Tuttavia, l'aumento dell'immunità innata osservata durante la gravidanza può previene le infezioni e spiegare l'assenza di una maggiore suscettibilità ai patogeni. Infatti l'evidenza di una maggiore suscettibilità alle infezioni nelle donne in gravidanza è piuttosto debole (Kourtis, 2014). Anche se le donne in gravidanza non sembrano essere più propense a contrarre infezioni in generale, le modificazioni dell'organismo materno associate alle alterazioni immunologiche che avvengono con l'avanzare della gravidanza possono determinare un aumento della gravità della malattia causata da alcuni patogeni (Kourtis et al, 2014).

Le stime d'incidenza di sepsi in gravidanza sono basate principalmente su studi retrospettivi e variano considerevolmente in ragione del metodo di rilevazione e delle definizioni utilizzate. In base ai dati di letteratura l'incidenza di sepsi in gravidanza varia dallo 0,1 allo 0,4/1000 parti per anno (Van Dillen 2013, Italian Obstetric Surveillance System 2014-2015). In Regione Lombardia secondo un'analisi delle SDO (Bauer, 2013; Angus, 2013; AHRQ), l'incidenza della sepsi materna è pari allo 0,33/1000 parti/anno (analisi effettuata su 49.984 parti dal 2009 al 2013).
Le stime di mortalità per sepsi materna variano dallo 0,6 all' 1,1/100.000 parti secondo dati australiani ed americani. La sepsi in gravidanza risulta responsabile del 10,7% delle morti materne (2003-2012) secondo un'analisi eseguita dall'Organizzazione Mondiale della Sanità. Egualmente nel nostro paese, dai risultati del progetto pilota sulla Sorveglianza della Mortalità Materna dell'Istituto Superiore di Sanità, la sepsi risulta la seconda causa di morte materna diretta, dopo l'emorragia ostetrica, con una percentuale pari al 10%. Da studi recenti si rileva che la sepsi materna ha una mortalità che va dall'1,8 al 17,6% e aumenta al 28-33% in caso di shock settico. Malgrado si sia assistito negli ultimi anni ad una riduzione delle morti materne legate ad infezioni e sepsi, la sepsi materna rappresenta a tutt'oggi una delle principali cause prevenibile di morte.

Nella popolazione ostetrica, come sottolineato dalle Linee Guida della Surviving Sepsis Campaign (SSC 2016) per la popolazione adulta, i fattori chiave in grado di migliorare significativamente la prognosi sono il riconoscimento rapido e la gestione tempestiva della sepsi; ciò si può attuare mediante l'impiego sistematico di una procedura di valutazione, di rivalutazione programmata e la definizione di soglie di allerta, con conseguente attivazione di azioni di risposta in base ad algoritmi decisionali, in sintesi grazie ad un "approccio clinico-organizzativo" del percorso assistenziale.

Al fine di proporre un documento operativo indirizzato a tutte le strutture regionali per l'identificazione precoce e la gestione della sepsi in ostetricia, Regione Lombardia ha attivato un

tavolo di lavoro multidisciplinare composto da Medici Ginecologi, Anestesisti-Rianimatori, Ostetriche e Risk Manager. Le raccomandazioni emerse si pongono quale riferimento per la pratica clinica in una logica tempo-dipendente, multiprofessionale e multidisciplinare e non sono da intendersi come indicazioni rigidamente assolute.
Il documento comprende i seguenti capitoli:

1. Prevenzione e fattori di rischio.
2. Sistemi di allerta e sepsi materna: identificazione e monitoraggio.
3. Nuove definizioni della sepsi materna e identificazione del rischio.
4. Primi interventi diagnostico terapeutici per la sepsi materna.

1. LA PREVENZIONE E I FATTORI DI RISCHIO

Questo capitolo esamina gli interventi mirati alla riduzione del rischio di infezioni/sepsi durante la gravidanza, il parto e periodo post-natale.

Razionale

Con il termine prevenzione s'intende l'insieme delle strategie e dei comportamenti "Evidence Based" atti a ridurre i fattori di rischio d'infezione/sepsi nella popolazione ostetrica. La prevenzione, soprattutto primaria, gioca un ruolo fondamentale per tutti noi.
La prevenzione è possibile grazie ad adeguati programmi di controllo delle infezioni che comprendono oltre ad uno stretto monitoraggio del fenomeno, la formazione del personale sulle pratiche di controllo delle infezioni con particolare attenzione ai fattori di rischio, l'utilizzo di metodiche e dispositivi appropriati per la pulizia, l'igiene e la disinfezione, l'introduzione di screening preventivi pre-chirurgici/pre-parto e una diffusa educazione sanitaria alla popolazione ostetrica. Nel maggio 2017 l'Organizzazione Mondiale della Salute (OMS) ha approvato una risoluzione mirata a migliorare non solo la diagnosi e la cura della sepsi, ma soprattutto la prevenzione, ponendo una particolare attenzione al controllo delle infezioni ed alla limitazione di un'ulteriore diffusione della resistenza antibiotica. E' infatti ampiamente dimostrato che l'attuazione delle misure di prevenzione, con particolare attenzione ai fattori di rischio, sia efficace nel ridurre l'incidenza di infezioni/sepsi nella popolazione ostetrica con un miglioramento della morbilità e mortalità ad esse associate.
Importante tenere sempre in considerazione i cambiamenti fisiologici in corso di gravidanza e l'impatto che possono avere sulla diagnosi e conseguentemente sulla gestione clinica (Tabella 1).

Tabella 1. Cambiamenti fisiologici in corso di gravidanza ed impatto sulla diagnosi e sulla gestione (Cordioli, 2013)

SISTEMA	CAMBIAMENTI fisiologici	IMPATTO SULLA DIAGNOSI/GESTIONE
**Cardiovascolare**	< Resistenze vascolari periferiche	Mascheramento dei segni/sintomi iniziali di sepsi
	< Pressione arteriosa	
	> Frequenza cardiaca	
	> Portata cardiaca	
**Sangue**	> Volume plasmatico<br>> Volume dei globuli rossi<br>Anemia	Segni di ipovolemia tardivi<br>Riduzione significativa dell'apporto di O2 ai tessuti
**Respiratorio**	> Tidal volume<br>< Volume residuo<br>> Ventilazione/minuto del 30-40%<br>> Frequenza respiratoria<br>< PaCO2	Ritardo nel compenso dell'alcalosi metabolica<br>Compromissione ossigenazione
**Renale**	Dilatazione uretero-pelvica<br>< Pressione ureterale dovuta a rilassamento muscolatura liscia Flaccidità vescicale<br>> Pressione intravescicale da compressione da parte dell'utero gravido<br>> Reflusso vescico-ureterale<br>> Flusso plasmatico renale<br>> Velocità filtrazione glomerulare<br>< Valori di azotemia e creatinina	Ritardata identificazione di danni renali dovuti alla sepsi<br>Predisposizione a Infezioni delle vie urinarie
**Gastrointestinali**	< Tono muscolare intestino<br>Ritardato svuotamento gastrico<br>Elevazione del diaframma<br>Modificazione della composizione biliare<br>> Produzione di citochine proinfiammatorie da parte delle cellule di Kupffer	> Rischio di traslazione batterica<br>> Rischio di polmonite da aspirazione<br>> Rischio di colestasi, iperbilirubinemia, ittero
**Coagulazione**	> Fattori VII,VIII, IX, X, XII di Von Willebrand e fibrinogeno<br>< Proteina S<br>< Attività fibrinolitica	> Rischio di eventi trombotici<br>> Rischio di DIC
**Genitale**	< pH vaginale<br>> Glicogeno nell'epitelio vaginale	> Rischio di corionamniosite

FATTORI DI RISCHIO

La valutazione dei fattori di rischio e la loro “presa in carico” costituisce il primo strumento di prevenzione delle infezioni/sepsi nella popolazione ostetrica.
I fattori di rischio sono relativi a fattori costituzionali e alla storia clinica della paziente ostetrica; sono inoltre differenti a seconda dei trimestri di gravidanza (Tabella 2).

MISURE PREVENTIVE

Le principali misure preventive delle infezioni/sepsi nella popolazione ostetrica comprendono:

- Il buon uso di pratiche clinico-assistenziali mirate al controllo delle infezioni (per es. metodiche e dispositivi appropriati per la pulizia, l’igiene e la disinfezione, protocolli di profilassi antibiotica, ecc).
- L’utilizzo di sistemi di monitoraggio e allerta (Modified Early Obstetric Warning Score, MEOWS) per favorire una precoce identificazione di infezione/sepsi (vedi capitolo 2).
- Programmi di educazione sanitaria sia per il personale ospedaliero che per la popolazione ostetrica per aumentare la consapevolezza del problema.
- L’utilizzo di check-list logistico-organizzative e la realizzazione di audit clinici per favorire l’attuazione delle misure di prevenzione e la diagnosi precoce.
- Il monitoraggio del fenomeno.

PREVENZIONE IN AMBIENTE OSPEDALIERO

- Corretto lavaggio delle mani (progetto OMS; formazione)
- Utilizzo da parte di operatori e visitatori di gel alcoolico per le mani.
- Evitare la rasatura dei peli pubici e perineali.
- Evitare, in assenza di fattori di rischio, esplorazioni vaginali in gravide con rottura prematura delle membrane.
- Evitare esplorazioni vaginali frequenti durante il travaglio a basso rischio.
- Attendere i tempi necessari per il terzo stadio senza utilizzare manovre invasive non opportune.
- Evitare l’esecuzione di tagli cesarei inappropriati.
- Mantenere rigorosa asepsi in sala operatoria.
- Rispettare il protocollo aziendale per il cateterismo vescicale.
- Rispettare il protocollo aziendale per la gestione degli accessi venosi periferici e centrali.
- Evitare l’uso non giustificato di antibiotici (attenersi ai protocolli aziendali) per rischio di antibioticoresistenza.
- Sensibilizzare all’atto della dimissione le pazienti ad identificare e segnalare precocemente gli eventuali sintomi di infezione: brividi, febbre, malessere, ipotensione, vomito, dolori addominali, perdite vaginali anomale.

Tabella 2. Fattori di rischio per infezioni/sepsi nella popolazione ostetrica.

ANTEPARTUM
**COSTITUZIONALI E ABITUDINI DI VITA**
Età > 40 e < 18 anni
BMI > 30
Fumo di sigarette
Basso livello socioeconomico
Minoranza etnica
**ANAMNESI CLINICA**
Diabete
Anemia
Patologie del sistema immunitario
Uso di farmaci immunosoppressori
Stretto contatto con persone/membri della famiglia affetti da infezioni batteriche/virali
**GRAVIDANZA**
Amniocentesi o altra procedura invasiva
Cerchiaggio cervicale
Rottura prolungata delle membrane amniocoriali
Secrezioni vaginali patologiche
**INTRAPARTUM**
Corionamniosite
Ripetute esplorazioni vaginali in travaglio
Parto operativo vaginale
Rimozione manuale della placenta
Taglio cesareo
Emorragia precoce
Ematomi e traumi perineali
**PERIODO POSTNATALE**
Ritenzione materiale placentare
Infezioni vie urinarie
Infezione ferita chirurgica/episiorrafia
Emorragia tardiva
Infezione della sede di inserzione del cateterino peridurale
Mastite

PREVENZIONE IN GRAVIDANZA

- Educazione alimentare:
- Evitare gli alimenti che possono trasmettere infezioni gravi come la listeriosi, salmonellosi, toxoplasmosi (linee guida del Ministero della Salute sull'alimentazione in gravidanza).
- Alimentazione corretta per prevenire un eccessivo aumento ponderale e l'insorgenza di sindrome metabolica.
- Igiene personale e lavaggio frequente delle mani.
- Evitare ambienti affollati.
- Evitare contatti diretti e continui con bambini ed adulti affetti da infezioni delle prime vie aeree (attenzione alle donne che lavorano con bambini piccoli per il rischio di infezione da Streptococco di Gruppo A).
- Sconsigliare viaggi in zone ad alto rischio infettivo.
- Raccomandare la vaccinazione antinfluenzale in ogni epoca della gestazione (il Ministero della Salute raccomanda la vaccinazione antiinfluenzale nel II e III trimestre).
- Prevenire e trattare l'anemia (linee guida ISS emorragia postpartum).
- Individuare alla prima visita ostetrica le gravide con basso livello socioeconomico e le donne che appartengono a minoranze etniche ed offrire loro un'assistenza addizionale (counselling ostetrico addizionale, supporto sociale addizionale e servizio di mediazione culturale) (NICE Guideline 2010, updated 2018).

PROFILASSI ANTIBIOTICA

La profilassi antibatterica ha lo scopo di prevenire le infezioni in presenza di fattori di rischio o durante le procedure invasive, causate dagli agenti patogeni che compongono la fisiologica flora endogena. Comunemente l'antibiotico è somministrato prima dell'esposizione potenziale al fine di ottenere livelli di concentrazione terapeutica tissutale al momento della procedura e per una durata limitata (<24 h). La molecola deve essere a lunga durata d'azione, con spettro d'azione ristretto al/i patogeno/i, economica, con scarsi effetti collaterali. Nella Tabella 3 è riportata una sintesi delle principali indicazioni.

Tabella 3. Indicazione e assenza di indicazione alla profilassi antibiotica in ambito ostetrico (WHO Recommendations for prevention and treatment of maternal peripartum infections, 2015).

RACCOMANDATA	NON RACCOMANDATA
In travaglio: con tampone positivo o in presenza di fattori di rischio per infezione da Streptococco di Gruppo B.	Fuori travaglio: anche se tampone positivo per infezione da Streptococco di Gruppo B.
Rottura prolungata delle membrane	Minaccia di parto prematuro a membrane integre.
Taglio cesareo elettivo e/o urgente	Liquido amniotico tinto di meconio.
Rimozione manuale della placenta	
Lacerazioni perineali di III e IV grado	

Raccomandazioni

- **Attuare nella popolazione ostetrica misure preventive d'infezione/sepsi la cui efficacia è comprovata da solide prove di evidenza.**
- **Utilizzare sistemi di monitoraggio e allerta (MEOWS) come strumento per una precoce identificazione di infezione/sepsi nella popolazione ostetrica.**
- **Riconoscere e riportare i fattori di rischio nella documentazione sanitaria in modo ben visibile ad ogni accesso ospedaliero e/o durante il ricovero della paziente ostetrica.**
- **Programmare la formazione del personale sanitario sui fattori di rischio e sulle misure preventive d'infezione/sepsi nella popolazione ostetrica.**

Azioni

- **Predisporre dei protocolli sulle misure di prevenzione d'infezione/sepsi raccomandati e specifici per la popolazione ostetrica.**
- **Programmare la formazione del personale sanitario sui fattori di rischio e sulle misure preventive d'infezione/sepsi nella popolazione ostetrica.**

2. SISTEMI DI ALLERTA E SEPSI MATERNA: IDENTIFICAZIONE E MONITORAGGIO

Razionale

La gravidanza, il parto e il periodo post-natale sono eventi fisiologici; il monitoraggio clinico durante il percorso nascita deve in ogni caso rappresentare una parte integrante del processo assistenziale al fine di poter riconoscere precocemente un eventuale deterioramento delle condizioni generali e prevenire esiti infausti. Emblematico è il caso della sepsi in cui le alterazioni fisiologiche della gravidanza possono mascherarne i segni e i sintomi e contribuire ad un ritardato riconoscimento finchè non sia evidente un severo deterioramento clinico (Tabella 1). *"Woman with serious illness, especially sepsis, may appear deceptively well before suddenly collapsing, often with little or no warning;"* (Centre for Maternal and Child Enquiries 2011). A supporto di ciò, i dati più recenti di mortalità materna indicano la sepsi come la seconda causa di morte materna diretta sottolineando la necessità di delineare dei percorsi dedicati di identificazione del rischio e di risposte appropriate di fronte alle patologie acute più frequenti e spesso letali.

I sistemi di allerta precoce ("Early Warning Score", EWS) sono stati sviluppati negli anni '90 sull'evidenza che minime alterazioni dei parametri fisiologici precedono il deterioramento clinico e che il loro riconoscimento con un'appropriata risposta possano migliorare l'esito di un paziente in stato di acuzie. Gli EWS adattati alla popolazione ostetrica ("Modified Early Obstetric Warning Score", MEOWS) sono stati introdotti più recentemente nei punti nascita con l'obiettivo di facilitare il riconoscimento del rischio e di delineare un percorso terapeutico assistenziale a seconda della gravità clinica per le donne che hanno o stanno sviluppando una patologia critica. Infatti i segni di shock imminente spesso non vengono riconosciuti precocemente in questa popolazione e la risposta è spesso disorganizzata in un quadro clinico molto complesso. Gli EWS/MEOWS si basano sulla rilevazione dei parametri vitali in una scala a punteggio o a codice colore che consente una veloce e condivisa valutazione dello stato clinico con l'obiettivo di fornire una misura riproducibile di quanto e se un paziente sia a rischio di deterioramento. Questo sistema di allerta permette inoltre di identificare (in base al punteggio/codice colore) il tipo di risposta da attivare in termini di tempi (frequenza di rilevazione/monitoraggio) e di modi (livello di allerta). Tali procedure favoriscono un approccio standardizzato al benessere materno con particolare attenzione al percorso d'intensificazione di cura (ove necessario) oltre a facilitare il lavoro di squadra e la comunicazione fra il personale sanitario.

L'implementazione del sistema di allerta MEOWS (con scheda cartacea o elettronica) segue le raccomandazioni del report sulle morti materne del 2007 e del 2011 (Confidential Maternal Enquiry Report, McClure et al, 2011) e dall'Istituto Superiore di Sanità del 2016 (Agenas: Linee di indirizzo clinico-organizzative per la prevenzione delle complicanze legate alla gravidanza) per l'inquadramento, il monitoraggio e la gestione della paziente ostetrica. I MEOWS hanno infatti un'alta sensibilità (89%) nel predire la morbilità e una ragionevole specificità (79%) per supportare il loro utilizzo nella popolazione ostetrica. I MEWS comunemente utilizzati per la popolazione adulta si sono dimostrati uno strumento accurato nell'identificare i pazienti con infezione a rischio di deterioramento clinico sottolineandone il ruolo chiave come strumento di screening in una patologia tempo-dipendente come la sepsi. Il precoce riconoscimento e quindi un trattamento tempestivo della sepsi rappresentano nella popolazione ostetrica (come in quella adulta) gli interventi chiave in grado di migliorarne la morbilità e mortalità. L'obiettivo di questo capitolo è quello di facilitare l'identificazione e la gestione della popolazione ostetrica potenzialmente settica attraverso l'utilizzo di un sistema di allerta precoce standardizzato (MEOWS).

Il presente documento fornisce, oltre allo strumento MEOWS, indicazioni sulla frequenza del monitoraggio clinico, sull'urgenza dell'intervento e sulle competenze richieste nei diversi livelli di gravità con particolare menzione ai casi di sepsi; mette inoltre in evidenza l'importanza che una risposta organizzata sia definita e condivisa in tutte le strutture ospedaliere con un punto nascita per favorire un precoce riconoscimento ed un intervento tempestivo nelle pazienti potenzialmente settiche.

LA SCHEDA MEOWS: IL SUO USO E LA RISPOSTA CLINICA

Il Gruppo di Lavoro Regionale propone l'utilizzo del sistema di allerta MEOWS presentato nelle Linee Guida Nazionali Irlandesi del 2014. Il MEOWS proposto si fonda sulla misurazione di 7 parametri fisiologici; il loro significato clinico e le modalità di registrazione sono riportati nell'Allegato 2.
Ciascun parametro è graduato in livelli, a ciascuno dei quali è attribuito un codice colore. La somma dei codici colore fornisce la misura dello scostamento dalla fisiologia normale ed il tipo di risposta da attuare. Per facilitarne l'uso e per favorire la standardizzazione dello strumento, viene proposta una scheda MEOWS a colori, che consente di cogliere immediatamente l'allarme di deterioramento (Figura 1).
Il sistema MEOWS con la relativa scheda prevede:

- la rilevazione dei parametri vitali secondo una frequenza determinata dal livello di rischio, dalla diagnosi di ricovero/complicanza e dall'ultima rilevazione (Tabella 6);
- un livello di allerta a seconda del punteggio dei codici colore che modula l'urgenza della risposta clinica e il livello di competenza professionale dei soccorsi (Figura 2 e Figura 3); il punteggio MEOWS fornisce 3 livelli di allerta corrispondenti rispettivamente ad 1 PARAMETRO GIALLO, 2 GIALLI o 1 ROSSO, >2 GIALLI o >1 ROSSO.

Il MEOWS deve essere utilizzato sistematicamente per fornire misure standardizzate dei livelli di gravità nelle patologie acute e come strumento di sorveglianza per tracciare l'andamento clinico e per avvertire di ogni eventuale possibile situazione di deterioramento.

Figura 1. Scheda Modified Early Obstetric Warning Score (MEOWS).

Contattare il medico se si rileva un trigger ROSSO o due GIALLI in qualsiasi momento																			
Anno	Data																		
	Ora																		
Freq resp/ min.	≥ 25																		≥ 25
	≥ 20																		≥ 20
	11 - 19																		11 - 19
	≤ 10																		≤ 10
SpO2	96 - 100%																		96 - 100%
	≤ 95%																		≤ 95%
Temp.°C	≥ 38.0																		≥ 38.0
	37.5-37.9																		37.5-37.9
	36.0-37.4																		36.0-37.4
	35.1-35.9																		35.1-35.9
	≤ 35.0																		≤ 35.0
Freq cardiaca	≥ 120																		≥ 120
	100 - 119																		100 - 119
	61 - 99																		61 - 99
	60 - 51																		60 - 51
	≤ 50																		≤ 50
Pressione sistolica	≥ 160																		≥ 160
	140 - 159																		140 - 159
	101 - 139																		101 - 139
	91 - 100																		91 - 100
	≤ 90																		≤ 90
PA diastolica	≥ 100																		≥ 100
	90 - 99																		90 - 99
	51 - 89																		51 - 89
	41 - 50																		41 - 50
	≤ 40																		≤ 40
Urine	Proteine																		Proteine
	Glucosio																		Glucosio
	Altro																		Altro
Risposta neurol.	Allerta (A)																		Allerta (A)
	Voce (V)																		Voce (V)
	Dolore (D)																		Dolore (D)
	Non risposta (NR)																		Non risposta (NR)
Totale zone GIALLE																			Totale zone GIALLE
Totale zone ROSSE																			Totale zone ROSSE
	Iniziali																		Iniziali

Tabella 4. Frequenza di rilevazione dei parametri MEOWS.

Situazione clinica	Frequenza minima di rilevazione parametri MEOWS
**Gravida a basso rischio con gravidanza non complicata (ricoverata)**	Valutazione completa al ricovero, quindi controlli ogni 12 ore incrementabili su indicazione clinica
**Postpartum donna a basso rischio con gravidanza e parto non complicati**	Valutazione completa di tutti i parametri dopo il parto. Quindi parametri MEOWS valutati ogni 12 ore fino alla dimissione se non vi è diversa indicazione clinica.
**Antenatale o postnatale**	**Frequenza minima di rilevazione parametri MEOWS**
**Disordini ipertensivi**	Rilevazione giornaliera di tutti i parametri inclusa analisi urine con valutazione dei parametri MEOWS ogni 4 ore
**Infezione materna sospetta o confermata**	Rilevazione giornaliera di tutti i parametri con valutazione dei parametri MEOWS ogni 4 ore
**Qualsiasi altro dubbio clinico**	Valutazione completa di tutti i segni vitali registrati almeno ogni 12 ore e successivamente sulla base del quadro clinico complessivo
**Situazione di emergenza**	Su valutazione clinica
**Trasfusione sangue**	Seguire i protocolli locali
**Dopo taglio cesareo o dopo chirurgia durante la gravidanza / periodo postnatale***	
**U**na valutazione completa dei segni vitali (esame delle urine non applicabile) deve essere registrata:<br>— ogni 15 minuti in sala risveglio (“recovery room”)<br>— poi ogni 30 minuti per 2 ore nel postpartum<br>— poi ogni 4-8 ore per 48 ore<br>— una volta al giorno fino alla dimissione<br><br>*La frequenza di rilevazione dei parametri deve seguire le indicazioni della scheda MEOWS	

Raccomandazioni

- **In tutte le donne in gravidanza o entro 42 giorni dal suo esito giunte al punto nascita (Pronto Soccorso ostetrico o generico) o già ricoverate, la rilevazione e il monitoraggio dei parametri vitali deve essere effettuato utilizzando uno score di allerta precoce specifico per la popolazione ostetrica (MEOWS).**
- **Nella donna in travaglio, dal momento dell’accesso in sala parto, la rilevazione dei parametri vitali, che segue il protocollo locale, deve essere invece riportata nel partogramma.**
- **Nella donna all’uscita dalla sala parto o dalla camera operatoria l’ultima rilevazione dei parametri vitali deve essere riportata sulla scheda MEOWS prima del trasferimento della paziente in reparto.**

— **La frequenza di rilevazione dei parametri del MEOWS è determinata dal rischio evolutivo, dalla diagnosi d'ingresso e/o complicanza clinica e dall'ultima valutazione (punteggio codice colore); il livello di allerta del MEOWS è determinato unicamente dalla valutazione (punteggio del codice colore).**

Figura 2. Diagramma per il percorso di applicazione della scheda MEOWS.

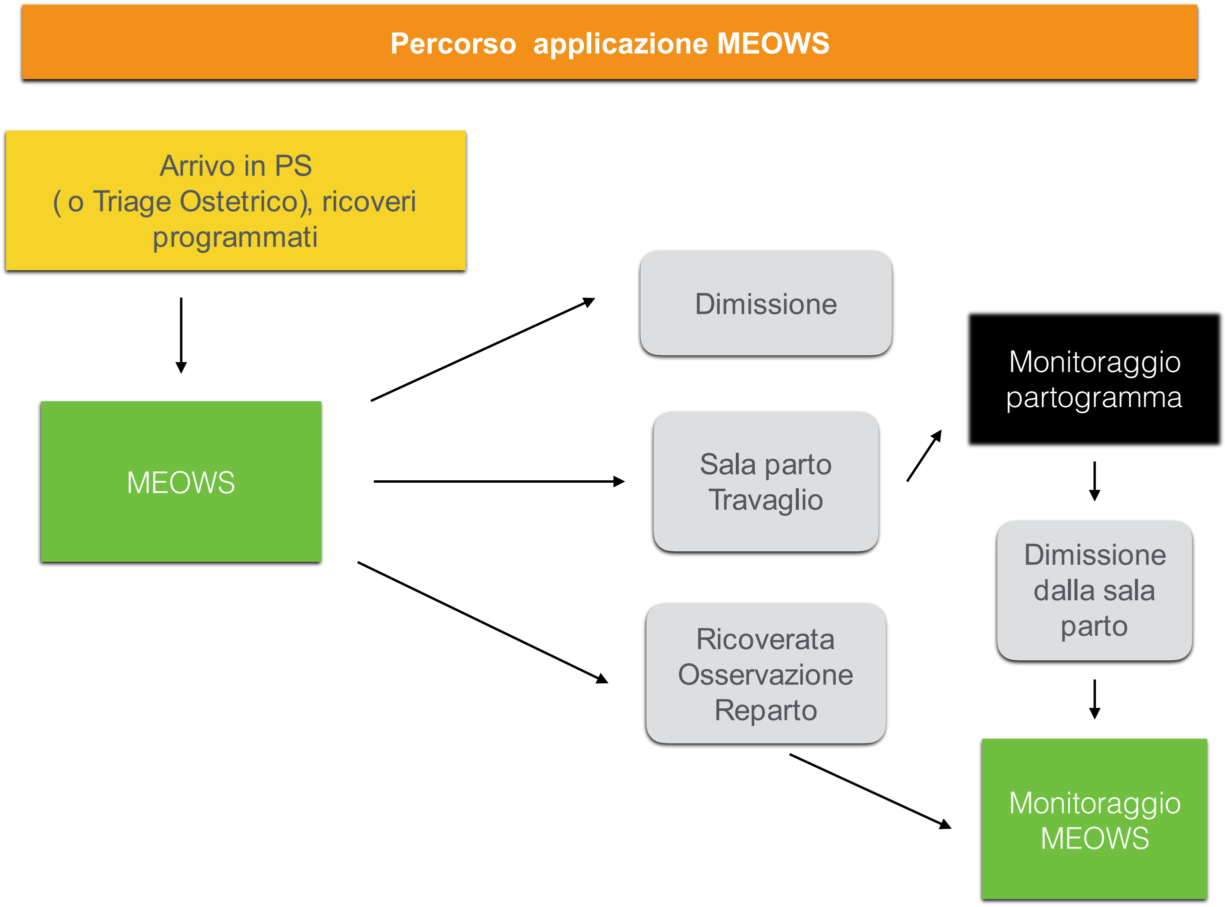

Azioni

— **Predisporre un programma di formazione sul sistema di valutazione sistematica e allerta standardizzato per la popolazione ostetrica (MEOWS) e sul suo utilizzo nella pratica clinica per tutti i professionisti sanitari coinvolti nell'assistenza della paziente in gravidanza/puerperio**.

INTENSIFICAZIONE DI CURA

In caso di scostamento dalla fisiologia normale, il punteggio dei codice colore dei MEOWS fornisce tre livelli di allerta che modulano l'urgenza della risposta clinica e il livello di competenza professionale dei soccorsi. La rilevazione di parametri vitali Gialli o Rossi comporta la necessità di aumentare la frequenza del monitoraggio e/o di richiedere oltre all'intervento dell'Ostetrica l'intervento del Medico Ginecologo e dell'Anestesista Rianimatore. I livelli di allerta modulano

l'urgenza della risposta clinica e il livello di competenza professionale dei soccorsi come indicato nel diagramma di flusso (Figura 3).

Figura 3. Diagramma di flusso MEOWS.

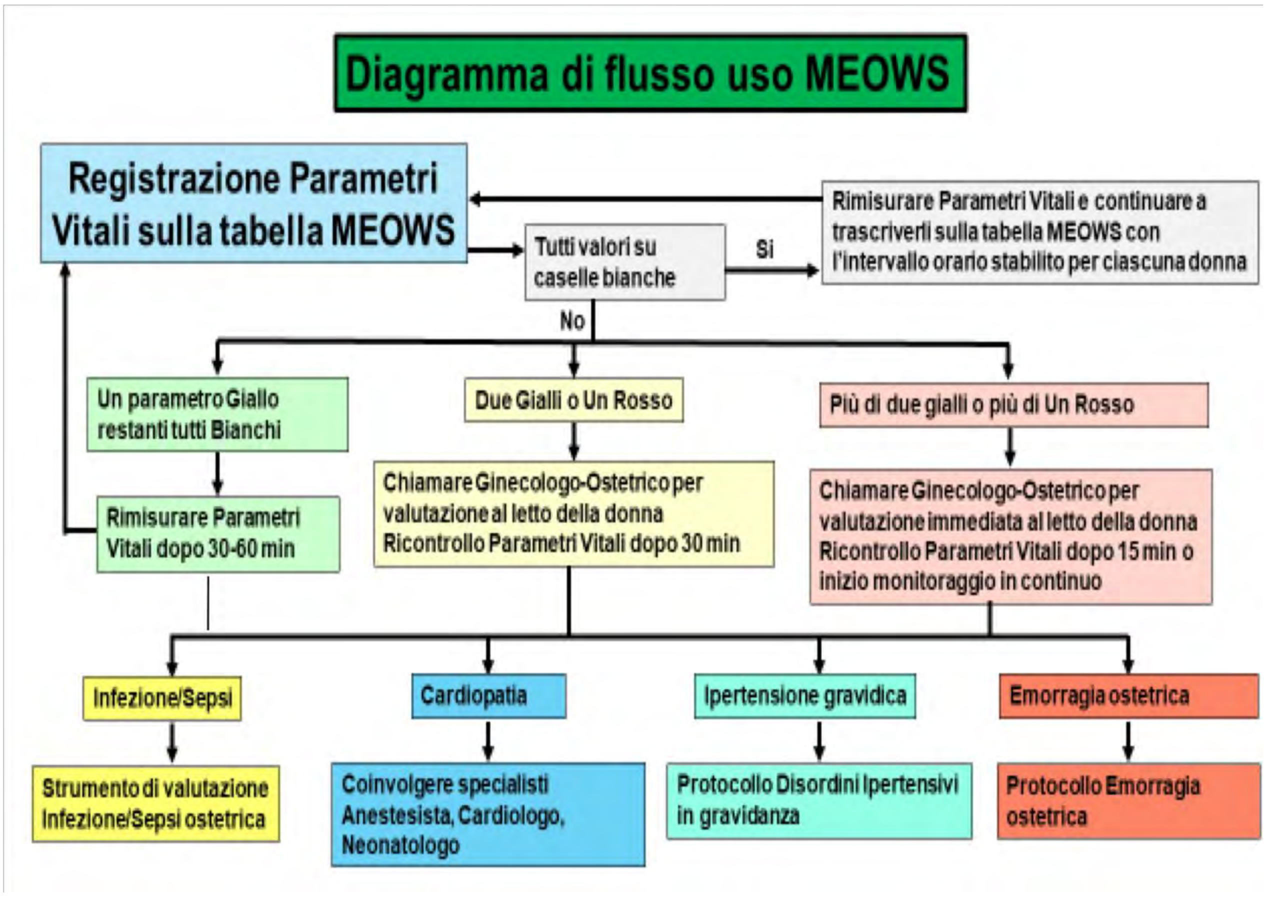

Raccomandazioni

- **Si raccomanda in caso di rilevazione di ≥1 parametro giallo MEOWS di considerare ed escludere il sospetto di infezione/sepsi/shock settico ricercando segni/sintomi di un'eventuale infezione e di un eventuale danno d'organo per poter confermare o escludere la diagnosi di sepsi/shock settico.**
- **In caso di rapido deterioramento del quadro clinico si raccomanda di predisporre un percorso di "intensificazione di cura"; tale percorso può richiedere l'intervento di personale più esperto, o di un consulto multidisciplinare per l'inquadramento, la valutazione e la gestione della paziente (Allegato 2).**
- **In caso di rapido deterioramento del quadro clinico nel caso in cui non sia possibile una valutazione immediata da parte del medico di guardia, è opportuno considerare il coinvolgimento di un altro Medico Ginecologo-Ostetrico e/o Anestesista-Rianimatore più esperto o coinvolgere precocemente il Direttore / Primario. L'impossibilità di una valutazione immediata da parte del medico di guardia è una precisa indicazione alla chiamata del medico reperibile.**

- In caso di rapido deterioramento del quadro clinico si raccomanda di valutare l'ipotesi di un eventuale trasferimento della paziente in una struttura più idonea se localmente non è possibile garantire l'intensificazione del percorso di cura. Ove il trasferimento non fosse possibile NON si deve ritardare l'effettuazione degli interventi diagnostico-terapeutici indicati (per es. esami di laboratorio, ECG, EGA o Rx torace, ecc).
- In caso di rapido deterioramento del quadro clinico, si raccomanda di effettuare la sequenza di valutazione ABCDE (Figura 4): Airway, Breathing, Circulation, Delivery, Esecuzione del percorso di cura. Il percorso di cura della gravida critica è illustrato in Figura 5.
- In caso di rapido deterioramento del quadro clinico si raccomanda di effettuare una rivalutazione continua delle condizioni cliniche della paziente, inclusi la sequenza ABCDE e l'esame obbiettivo, oltre alla revisione della documentazione clinica e la raccolta anamnestica.
- In caso di deterioramento del quadro clinico, in attesa della valutazione da parte del Medico, si raccomanda all'Ostetrica di intensificare il monitoraggio e l'assistenza alla paziente secondo lo schema riportato nella Tabella 4.
- In caso di deterioramento del quadro clinico si raccomanda di coinvolgere precocemente competenze multidisciplinari (Medico Ginecologo, Infettivologo, Anestesista Rianimatore).

Figura 4. Sequenza ABCDE per la valutazione della paziente con segni/sintomi di deterioramento clinico.

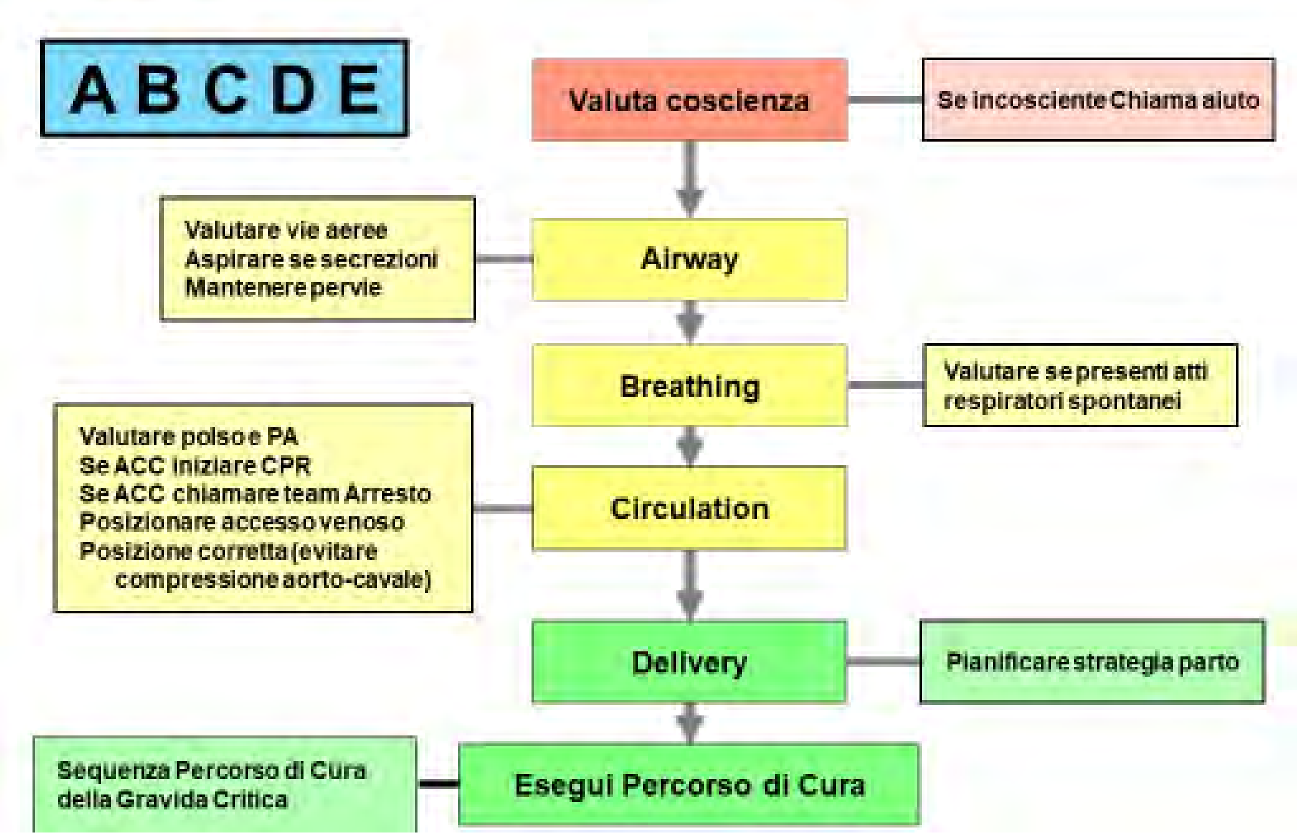

Figura 5. Percorso di cura della gravida critica.

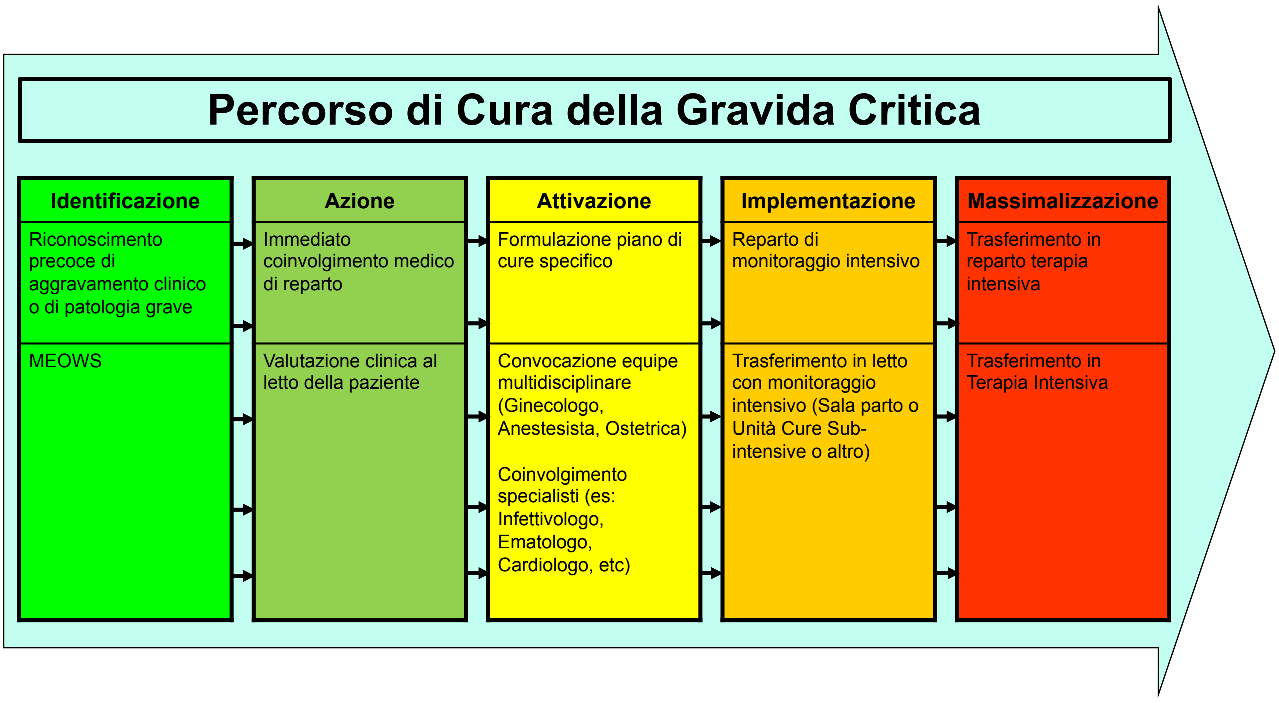

Azioni

— **Predisporre delle procedure operative sul percorso d'intensificazione di cure della paziente ostetrica in deterioramento clinico.**

— **Definire le procedure di attivazione (tempi e modi) dell'equipe equipe multidisciplinare coinvolte nella gestione della paziente ostetrica in deterioramento clinico.**

UTILIZZO DEI MEOWS NELL'IDENTIFICAZIONE E NEL MONITORAGGIO DELLA PAZIENTE POTENZIALMENTE SETTICA

L'utilizzo degli EWS nell'identificazione delle fasi acute di malattie e nella predisposizione di un adeguato piano assistenziale ne sottolinea la loro funzione nelle patologie tempo-dipendenti come la sepsi. La diagnosi precoce e il trattamento tempestivo della sepsi sono nella paziente ostetrica, come nel paziente adulto, gli interventi chiave in grado di migliorarne la morbilità e la mortalità. Nello specifico i MEOWS possono giocare un ruolo chiave nel percorso assistenziale della sepsi materna attraverso:

1. L'identificazione di pazienti con infezioni/sepsi; un'alterazione dei parametri vitali rilevata secondo il sistema di allerta MEOWS è spesso il primo segnale di infezione/sepsi come nel caso di un'alterazione del sensorio, di tachicardia o febbre evidenziabili da uno o più codici colore giallo e/o rosso. In caso di riscontro di ≥1 parametro giallo MEOWS bisogna considerare sempre il sospetto di un'infezione/sepsi/shock settico ricercando i segni e/o sintomi relativi alla presenza di una presunta o accertata infezione e segni e/o sintomi relativi alla presenza di un danno d'organo non altrimenti spiegabile (Figura 1); il binomio: presunta o accertata infezione + danno d'organo non altrimenti spiegabile sancisce la diagnosi di sepsi.

2. L'identificazione di un eventuale danno d'organo nella paziente con sospetta o accertata infezione; in una paziente con segni/sintomi d'infezione alcuni parametri MEOWS possono essi stessi se alterati (parametri giallo o rosso) smascherare un danno d'organo, come nel caso di ipotensione, incremento della frequenza respiratoria e riduzione della SpO2 o alterazione del sensorio. Ad ogni valutazione della paziente ostetrica ed in particolare nel sospetto di una infezione è pertanto fondamentale effettuare una valutazione dei parametri vitali mediante il sistema di allerta MEOWS.

3. L'identificazione del rischio di deterioramento fra le pazienti con infezioni/sepsi; come proposto dal Gruppo di Lavoro regionale in caso di una donna in gravidanza o durante il periodo post-natale, giunta al punto nascita o già ricoverata, in presenza di infezione il sistema di allerta MEOWS è in grado di stratificare il rischio evolutivo e di definire il corrispettivo percorso diagnostico-terapeutico assistenziale come indicato nel Capitolo 3:
    - RISCHIO INTERMEDIO (segni/sintomi d'infezione + 2 gialli o 1 rosso MEOWS) richiede sempre la valutazione del SOFA score modificato, la misurazione dei lattati sierici e l'effettuazione dei Sepsis Six.
    - RISCHIO ALTO (segni/sintomi d'infezione + >2 gialli o >1 rosso MEOWS o SOFA score modificato ≥1 o lattati >2 mmol/l) richiede sempre l'intervento dell'Anestesista-Rianimatore e l'effettuazione dei Sepsis Six.

Raccomandazioni

- **In caso di riscontro di ≥1 codice colore giallo del MEOWS bisogna considerare sempre il sospetto di un'infezione/sepsi/shock settico ricercando i segni e/o sintomi relativi alla presenza di una presunta o accertata infezione e segni e/o sintomi relativi alla presenza di un danno d'organo non altrimenti spiegabile.**
- **Ad ogni valutazione della paziente ostetrica ed in particolare nel sospetto di infezione è fondamentale effettuare una valutazione dei parametri vitali mediante il sistema di allerta MEOWS con l'obbiettivo di smascherare un eventuale danno d'organo.**
- **In una paziente ostetrica con segni/sintomi d'infezione completare sempre la valutazione dei parametri vitali mediante MEOWS; tale approccio suggerisce una stratificazione del rischio, la definizione del corrispettivo percorso diagnostico-terapeutico assistenziale.**
- **In qualsiasi donna in cui vi sia il sospetto di sepsi, va iniziato immediatamente il percorso d'intensificazione di cura; l'eventuale trasferimento in un reparto intensivo deve essere valutato dall'Anestesista Rianimatore.**

Azione

- **Predisporre procedure operative sull'utilizzo dei MEOWS per la paziente ostetrica con sospetta infezione/sepsi/shock settico.**

Razionale

Una documentazione non adeguata (es. mancanza di indicazioni di monitoraggio, prescrizioni terapeutiche imprecise, calligrafia illeggibile, sigle non convenzionali) è possibile fonte di eventi avversi nella gestione della paziente critica.

Raccomandazioni

— **Si raccomanda di riportare costantemente tutti i parametri vitali e le risposte cliniche del sistema di allerta MEOWS annotando ora ed operatore**.

Azioni

— **Predisporre l'utilizzo di una scheda MEOWS standardizzata come unica scheda di registrazione dei parametri vitali.**

— **Predisporre dei sistemi di monitoraggio di compliance all'utilizzo dei MEOWS.**

3. NUOVE DEFINIZIONI DELLA SEPSI MATERNA E IDENTIFICAZIONE DEL RISCHIO

Razionale

I dati di letteratura sono concordi nell'affermare che la precoce identificazione nella popolazione ostetrica delle pazienti con sepsi/shock settico ed il conseguente intervento terapeutico tempestivo hanno un impatto significativo sulla sopravvivenza. Ridurre la mortalità comporta quindi definire percorsi condivisi in merito al precoce riconoscimento delle infezioni e delle insufficienze d'organo sepsi correlate al di fuori dell'area critica in una popolazione in cui l'evoluzione clinica è più rapida e spesso più letale. La sepsi materna è quindi da definirsi un'emergenza ostetrica.
Questo documento si propone di fornire un approccio pratico alla valutazione delle pazienti con infezione/sepsi/shock settico in un contesto di cure primarie (in pronto soccorso, in sala parto o in degenza), da parte di Medici Ginecologi e Ostetriche includendo strumenti pratici per valutare i fattori di rischio, oltre ad un elenco dettagliato e strutturato di potenziali segni e sintomi clinici di interesse. Si ritiene che l'utilizzo di uno strumento di valutazione semplice e standardizzato possa aiutare i professionisti sanitari a identificare le pazienti più gravi, che richiedono trattamenti potenzialmente salvavita.

DEFINIZIONI

Definizione di sepsi per la donna in gravidanza e in puerperio

Nel 2016 l'Organizzazione Mondiale della Sanità (OMS) ha proposto la nuova definizione di sepsi materna:

> "La Sepsi materna è una condizione pericolosa per la vita definita come una disfunzione d'organo conseguente a un'infezione contratta durante la gravidanza, il parto, e il periodo post-aborto o il periodo post-partum (ossia lasso di tempo fra la rottura delle membrane o il parto e il 42° giorno del puerperio)".

Tale definizione è stata sviluppata sulla base di una revisione sistematica delle definizioni esistenti (inclusa la Third International Consensus Definitions for Sepsis and Septic Shock 2016 per la popolazione adulta) ed attraverso una consulenza tecnica internazionale. Questa nuova proposta della OMS si allinea alla recente definizione di sepsi nell'adulto considerando la sepsi come una risposta sregolata e rischiosa per la vita causata da un'infezione.

Criteri diagnostici di sepsi e shock settico per la donna in gravidanza e in puerperio

La nuova definizione stabilisce che il binomio: infezione + danno d'organo è alla base della diagnosi di sepsi materna come di quella dell'adulto. Tuttavia l'OMS non ne ha definito i criteri diagnostici considerando il sistema di valutazione del danno d'organo proposto per la sepsi dell'adulto (il SOFA score –Sequential Organ Failure Assessment) non sufficientemente validato per la sepsi materna sia per le alterazioni fisiologiche caratteristiche della gravidanza che per la sua complessità in quanto basato non unicamente su criteri clinici "bedside". In attesa dell'elaborazione dei criteri diagnostici dell'OMS rimandiamo alla proposta del Gruppo di Approfondimento Tecnico (GAT) di Regione Lombardia che suggerisce l'utilizzo di criteri diagnostici già utilizzati per la popolazione adulta adattati alla condizione fisiologica della gravidanza. Tale strategia è in accordo con quella proposta dall'Associazione di Medicina Ostetrica dell'Australia e Nuova Zelanda.

Pertanto secondo le definizioni:

**La SEPSI MATERNA è una condizione pericolosa per la vita definita come una disfunzione d'organo risultante da un'infezione durante la gravidanza, il parto, il periodo postaborto o il periodo postpartum**

Criteri diagnostici suggeriti. La diagnosi di Sepsi materna prevede il riscontro di un'infezione presunta o accertata associata al danno di uno o più organi, che si può evidenziare con uno o più dei seguenti criteri:

- Necessità O2 per mantenere SpO2>95% o PaO2/FiO2 <400.
- Valore di piastrine <100 x 10^6/L.
- Valore di bilirubinemia > 1,2mg/dL.
- PAS <90 mmHg.
- Paziente risvegliabile su stimolo verbale/doloroso o incosciente.
- Valore di creatininemia >1,2mg/dL.

Tali criteri sono riassunti con un incremento del punteggio di SOFA score modificato ≥1 (Tabella 5).

Tabella 5. SOFA score modificato

Organo/apparato	Parametro	Punteggio 0	Punteggio 1
**RESPIRATORIO**	Necessità O2 per mantenere SpO2>95%<br>PaO2/FiO2 (se disponibile EGA)	NO<br>>400	SI<br><400
**COAGULAZIONE**	Piastrine x 10^6/L	>100	<100
**EPATICO**	Bilirubinemia mg/dL	<1,2	>1,2
**CARDIOVASCOLARE**	PAS mmHg	>90	<90
**NEUROLOGICO**	Scala AVPU	Sveglia (A)	Risvegliabile su stimolo verbale (V), doloroso (D) o incosciente
**RENALE**	Creatininemia mg/dL	<1,2	>1,2

Il SOFA (Sequential Organ Failure Assessment) score è un punteggio per la valutazione della disfunzione d'organo nei pazienti critici che si basa su parametri vitali ed esami ematochimici. Il SOFA score attribuisce ad ogni organo/apparato considerato (respiratorio, cardiovascolare, coagulatorio, epatico, renale e nervoso) un parametro di valutazione (es. valore bilirubina per valutare la funzionalità del fegato) a cui, a seconda del grado di disfunzione, viene attribuito un punteggio fra 0 e 4; la somma del punteggio di ogni singolo apparato/sistema costituisce il SOFA score totale (0-24). Il SOFA score basale in assenza di danni d'organo è 0.

Per la popolazione ostetrica il Gruppo di Lavoro regionale, in attesa dei criteri diagnostici che verranno prossimamente definiti dalla OMS, ha proposto dei criteri diagnostici di danno d'organo riassunti in un SOFA score modificato (mSOFA). Tale strategia si basa sulla necessità: a) di avere uno strumento agevole e di facile utilizzo al di fuori dell'area critica (risulterebbe infatti difficile calcolare il GCS come prelevare una EGA alla prima valutazione in presenza di una sospetta infezione al di fuori dall'area critica); b) di effettuare una diagnosi precoce in una popolazione in cui

l'evoluzione clinica può essere rapida e spesso fatale; c) di avere uno score che consideri le alterazioni fisiologiche tipiche della gravidanza;
L'elaborazione del SOFA score modificato si basa sulle considerazioni qui di seguito riportate:

- Il SOFA score modificato è sovrapponibile al SOFA score utilizzato per la popolazione adulta con le variazioni qui di seguito riportate.
- Il SOFA score modificato riporta unicamente i valori relativi al punteggio 0 (valori fisiologici nella popolazione ostetrica) e al punteggio 1 (valori patologici soglia indicativi di possibile danno d'organo sepsi correlato nella popolazione ostetrica), rendendo più agevoli l'utilizzo di questo score (anche mnemonicamente) e quindi la diagnosi; tutti i valori superiori a quelli relativi al punteggio 1 verranno egualmente considerati patologici.
- Nella popolazione ostetrica un punteggio ≥1 del SOFA score modificato (e non ≥2 come nella popolazione adulta) rappresenta il criterio diagnostico per identificare un danno d'organo sepsi correlato in presenza di un'infezione certa o presunta; tale strategia è supportata dalle alterazioni fisiologiche indotte dalla gravidanza (come descritto al punto 4) e dalla rapida evoluzione clinica indotta dalla sepsi in questa popolazione offrendo cosi uno strumento di screening più sensibile e più precoce.
- Il SOFA score modificato definisce dei valori soglia specifici per la popolazione ostetrica. Nella popolazione ostetrica il valore di creatininemia basale è significativamente ridotto con valori normali compresi fra 0,4-0,9 mg/dL, pertanto un valore soglia di 1,2mg/dL viene considerato come identificativo di un danno d'organo severo (punteggio 1), egualmente per il valore di bilirubina.
- Il SOFA score modificato offre delle semplificazioni per la valutazione dell'apparato respiratorio e dello stato neurologico. Il Glasgow Coma Score (GCS) (non routinariamente utilizzato nelle degenze di ostetricia e ginecologia) è stato sostituito dalla scala di valutazione AVPU (alert, vigilant, pain, unconscious), già utilizzata nel sistema di allerta/monitoraggio MEOWS; il rapporto PaO2/FiO2 (che richiede una emogasanalisi arteriosa) può essere in prima istanza sostituito dalla "necessità o meno di O2 per mantenere una saturazione arteriosa > 95%"; entrambe le valutazioni dovranno poi essere confermate dalla valutazione del GCS e dal rapporto PaO2/FiO2 una volta che la paziente sarà presa in carico dall'Anestesista-Rianimatore e/o eventuale ricovero in area critica.
- La popolazione ostetrica può avere una PAS <90mmHg, per cui il SOFA score modificato deve essere interpretato nel contesto dei valori pressori abituali della paziente gravida o in puerperio.
- Nella popolazione generale un SOFA score ≥2 è associato ad una mortalità del 10% per ogni danno d'organo, mentre per la popolazione ostetrica ad oggi non vi sono associazioni validate fra un valore di SOFA e mortalità predetta; pertanto l'obbiettivo di questo strumento è unicamente quello di facilitare il riconoscimento di un danno d'organo sepsi correlato e quindi la diagnosi di sepsi il più precocemente possibile in una popolazione peculiare come quella ostetrica.

Operativamente i criteri diagnostici di danno d'organo (SOFA score modificato ≥1) necessitano dei valori di creatininemia, bilirubinemia e numero di piastrine, dati spesso tardivi rispetto al sospetto clinico; è fondamentale procedere ad una sua prima valutazione in attesa degli esami ematochimici e non ritardare i primi interventi diagnostico-terapeutici in caso di forte sospetto clinico e/o alterazione parametri vitali MEOWS.

Lo **SHOCK SETTICO** è un sottoinsieme della sepsi in cui le alterazioni circolatorie, cellulari e metaboliche presenti sono associate a un maggior rischio di mortalità rispetto alla sola sepsi

Criteri diagnostici suggeriti. La diagnosi di shock settico prevede il riscontro di un'infezione presunta o accertata associata ad ipotensione con necessità di somministrare farmaci vasopressori per mantenere una PAM ≥65mmHg e una lattacidemia >2mmol/L dopo adeguata resuscitazione volemica.

- Per adeguata resuscitazione volemica s'intende la somministrazione di 30ml/kg di cristalloidi in boli ripetuti di 500 ml in 1 ora.
- Per definire un paziente in shock settico è indispensabile valutare la risposta al carico volemico: se l'ipotensione arteriosa associata ad un valore di lattati >2mmol/L persiste, la diagnosi è di shock settico, se invece si risolve la diagnosi è di sepsi.

La diagnosi di presunta o accertata sepsi/shock settico non deve essere ritardata in attesa di evidenze microbiologiche; i segni/sintomi d'infezione associati ad un danno d'organo (e/o ipotensione associata ad incremento dei lattati) non altrimenti spiegabile sanciscono la diagnosi.

Quick-SOFA (o qSOFA)

Essendo consci della difficoltà di calcolare il SOFA score al di fuori delle Unità di Terapia Intensiva, gli Autori delle nuove LG nella popolazione adulta hanno proposto un surrogato semplificato il qSOFA. Il qSOFA, dove la prima lettera sta a indicare "quick", veloce, da usare rapidamente al letto del paziente (BEDSIDE) senza la necessità di esami di laboratorio, indaga tre semplici parametri, ovvero pressione arteriosa, frequenza respiratoria e stato mentale. Se almeno 2 criteri sono soddisfatti – tra tachipnea (FR≥22 atti/min), ipotensione (PAS≤100 mm/Hg) e un'alterazione dello stato sensorio – il paziente con sospetta/accertata infezione è a rischio di mortalità ospedaliera o di ricovero prolungato in area critica. Il ruolo di questo score è quindi quello di identificare fra i pazienti infetti quelli a maggior rischio di evoluzione negativa e non quello di diagnosi nè di infezione nè di sepsi. Ad oggi **il Gruppo di Lavoro regionale non suggerisce l'utilizzo di questo score per la popolazione ostetrica** per i seguenti motivi: a) tale score non ha alcuna validazione nella popolazione ostetrica; b) i valori soglia delle variabili che lo compongono non sono utilizzabili nella paziente gravida (la frequenza respiratoria basale è più alta e la pressione arteriosa sistolica più bassa rispetto alla popolazione adulta); c) dati di letteratura indicano che la sensibilità degli EWS è superiore a quella del qSOFA per identificare pazienti a rischio di evoluzione negativa; d) è preferibile l'utilizzo di uno strumento che identifichi un rischio di evoluzione negativa non solo fra i pazienti infetti.

COME IDENTIFICARE E FARE DIAGNOSI PRECOCEMENTE DI SEPSI/SHOCK SETTICO

La diagnosi di sepsi/shock settico parte da un alto indice di sospetto clinico del professionista sanitario.

La diagnosi di sepsi/shock settico deve essere sospettata in presenza di uno o più di questi elementi secondo l'algoritmo riportato in Figura 1:

- presenza di fattori di rischio per infezioni/sepsi (predisposizione) (Tabella1),
- segni e sintomi d'infezione,
- alterazione dei parametri vitali,
- riscontro di un danno d'organo,
- bradicardia o tachicardia fetale, morte fetale o perinatale, aborto o travaglio prematuro.

Nella pratica clinica:

- **Predisposizione**: nella popolazione ostetrica la presenza di fattori di rischio specifici, variabili a seconda del periodo gestazionale, è associata ad un'aumentata probabilità di contrarre infezioni/sepsi. Il loro riconoscimento e la loro tracciabilità sulla documentazione clinica facilitano l'eventuale diagnosi d'infezione/sepsi in caso di un quadro clinico ancora poco definito. Ad ogni accesso ospedaliero e alle visite periodiche durante il ricovero è quindi fondamentale valutare la presenza/nuova insorgenza di fattori di rischio per infezioni/sepsi (Tabella 1)**;** tale dato deve essere riportato nella documentazione clinica e ben visibile da parte di tutti gli operatori sanitari coinvolti nel percorso della paziente gravida/in puerperio.
- **Sospetto clinico d'infezione**: Le infezioni in gravidanza non sono rare e sono potenzialmente pericolose per la mamma e per il feto. I segni e sintomi clinici d'infezione variano a seconda della sede e spesso sono subdoli per le alterazioni fisiologiche tipiche della gravidanza. Ad ogni valutazione è fondamentale effettuare un'accurata anamnesi (sintomi riferiti dalla paziente) e/o un attento esame clinico (segni/sintomi rilevati dall'operatore) che permettano di identificare l'eventuale presenza di infezione in atto (Tabella 6).

  In caso di presunta o accertata infezione considerare sempre il sospetto di una sepsi /shock settico ricercando segni/sintomi di un eventuale danno d'organo prima attraverso la valutazione dei parametri vitali mediante lo score MEOWS e degli esami ematochimici di laboratorio, e successivamente, per confermare la diagnosi mediante i criteri diagnostici di danno d'organo (SOFA score modificato).

Tabella 6. Segni e sintomi clinici suggestivi di infezione per la donna in gravidanza e puerperio.

Iperpiressia o ipotermia
Sintomi simil influenzali
Cefalea e/o rigidità nucale e/o stato confusionale/disorientamento
Diarrea e/o vomito
Rush cutaneo
Dolore e tensione addominale/ pelvica
Perdite vaginali purulente o maleodoranti
Liquido amniotico purulento o maleodorante
Segni di mastite
Segni di cellulite/infezione ferita chirurgica/infezione perineale
Difficoltà respiratoria (frequenza respiratoria elevata e/o utilizzo muscolatura accessoria e/o ipossiemia)
Sintomi respiratori (tosse produttiva, faringodinia ecc)
Sintomi urinari (disuria, urine torbide ecc)
Segni infezione associata a catetere intravascolare (edema, rossore…)
Segni d'infezione fetale o neonatale
Leucocitosi o leucopenia
Incremento degli indici di flogosi (PCR, PCT)

- **Alterazione dei parametri vitali.** Un'alterazione dei parametri vitali rilevata secondo il sistema di allerta MEOWS è spesso il primo segnale di infezione/sepsi. In caso di riscontro di ≥2 parametri gialli o ≥1 rosso MEOWS bisogna considerare sempre il sospetto di un' infezione/sepsi /shock settico ricercando i segni e/o sintomi relativi alla presenza di una presunta o accertata infezione e segni e/o sintomi relativi alla presenza di un danno d'organo non altrimenti spiegabile. In una paziente con infezione/sepsi/shock settico alcuni parametri MEOWS possono essi stessi evidenziare se patologici un danno d'organo, come nel caso della pressione arteriosa sistolica o della frequenza respiratoria /SpO2 o della valutazione neurologica. Ad ogni valutazione della paziente ostetrica è pertanto fondamentale effettuare una valutazione dei parametri vitali mediante il sistema di allerta MEOWS.
- **Riscontro di uno o più danni d'organo.** Il danno d'organo sepsi correlato è il risultato della risposta sregolata dell'organismo all'infezione. E' un evento pericoloso per la mamma e per il feto ed è spesso il primo segnale di sepsi che giunge alla valutazione del professionista sanitario. Il danno d'organo viene sospettato in presenza di un'alterazione di alcuni parametri vitali MEOWS rilevata da ≥1 parametro giallo e/o rosso e/o da un'alterazione degli esami di laboratorio (valore di creatininemia, bilirubinemia e conteggio piastrinico per identificare rispettivamente il danno renale, epatico e coagulatorio). Se tali alterazioni corrispondono ai criteri di identificazione del danno d'organo (incremento del punteggio di SOFA score modificato ≥1) in presenza di un'infezione certa o presunta si pone la diagnosi di sepsi. In presenza di un danno d'organo non altrimenti spiegabile è fondamentale ricercare sempre e tempestivamente un'infezione per identificare o escludere un'eventuale sepsi/shock settico.
- **Bradicardia o tachicardia fetale, morte fetale o perinatale, aborto o travaglio prematuro** sono eventi che possono essere indotti da un'infezione/sepsi materna; in tutti questi casi è fondamentale ricercare sempre e tempestivamente un'infezione e un danno d'organo per escludere o confermare il sospetto diagnostico.

In sintesi:

- Nella pratica clinica è importante ricercare in tutti i pazienti con un'infezione presunta o accertata segni/sintomi di danno d'organo e in tutti pazienti con danno d'organo non altrimenti spiegabile (es. ipotensione o alterazione del sensorio ecc.) ricercare segni/sintomi di infezione presunta o accertata per poter confermare o escludere la diagnosi di sepsi/shock settico; nei pazienti con alterazione dei parametri vitali secondo MEOWS (≥2 gialli o ≥1 rosso) è importante ricercare segni/sintomi di un'eventuale infezione e di un eventuale danno d'organo per poter confermare o escludere la diagnosi di sepsi/shock settico.
- Nella pratica clinica in tutti i pazienti con un'infezione presunta o accertata la ricerca del danno d'organo deve essere effettuata mediante la rilevazione dei parametri vitali con il sistema di allerta MEOWS e l'invio di esami ematochimici (pannello sepsi) e validata con la misura del SOFA score modificato.

Figura 1. Algoritmo diagnostico di sepsi/shock settico.

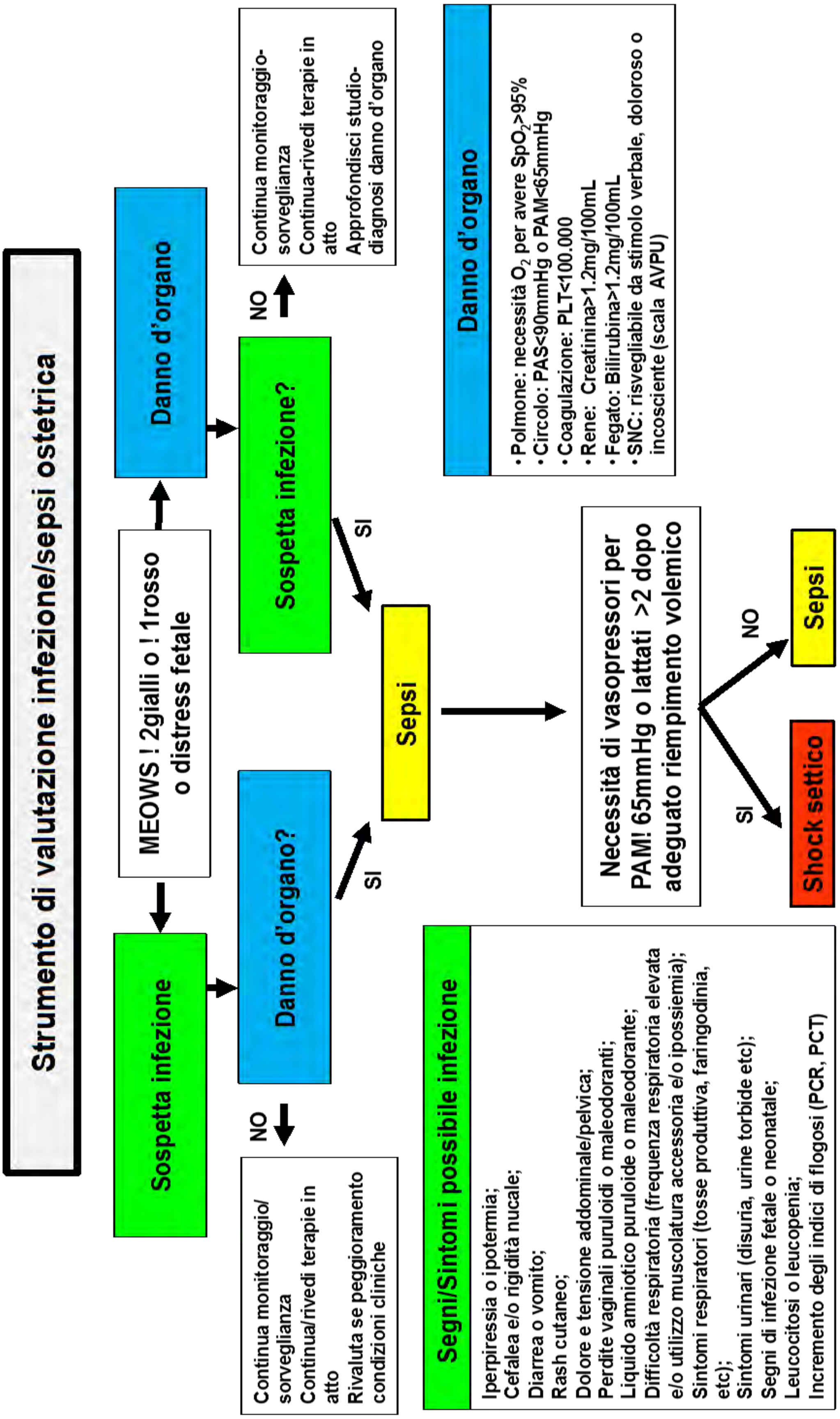

IDENTIFICAZIONE DEL RISCHIO

In caso di una donna in gravidanza o in puerperio, giunta al punto nascita o già ricoverata, in caso di sospetta infezione il percorso diagnostico-terapeutico assistenziale deve prevedere quanto di seguito riportato:

- valutazione dell'Ostetrica e del Medico Ginecologo di guardia,
- rilevazione dei parametri vitali secondo il sistema di allerta/monitoraggio MEOWS,
- valutazione fetale.

Le azioni successive sono definite in base alla rilevazione o meno di un allarme di deterioramento clinico identificabile con il codice colore MEOWS. Sul codice colore MEOWS in presenza di sospetta o accertata infezione viene identificato un livello di rischio con le relative modalità operative (Figura 2).

RISCHIO BASSO (MEOWS 1 PARAMETRO GIALLO E SOSPETTA O CERTA INFEZIONE)

La condizione di rischio basso è definita dalla presenza di infezione sospetta o certa e dalla rilevazione di 1 parametro giallo secondo MEOWS.
In tale condizione il Medico Ginecologo valuta i parametri vitali, il benessere fetale, stabilisce l'eventuale esecuzione di esami ematochimici ("pannello sepsi": emocromo completo con formula, lattati, elettroliti, azotemia, creatininemia, bilirubinemia, PT-PTT, PCR o PCT) e/o esami radiologici e/o l'eventuale richiesta di consulenza specialistica, procedendo al trattamento terapeutico più adeguato in base alle condizioni cliniche della paziente. Il monitoraggio dei parametri vitali deve proseguire secondo l'algoritmo MEOWS.

RISCHIO INTERMEDIO (MEOWS 2 GIALLI O 1 ROSSO E SOSPETTA O CERTA INFEZIONE)

La condizione di rischio intermedio è definita dalla presenza di una sospetta o certa infezione e dalla rilevazione di 2 parametri gialli o 1 rosso secondo MEOWS.
In tale condizione il percorso diagnostico-assistenziale prevede:

- monitoraggio dei PV secondo algoritmo MEOWS;
- supporto delle funzioni vitali ove necessario;
- esami ematochimici "pannello sepsi": emocromo completo con formula, lattati;
- elettroliti, azotemia, creatininemia, bilirubinemia, PT-PTT, PCR o PCT;
- ricerca il danno d'organo (criteri diagnostici di danno d'organo o SOFA score modificato);
- effettuazione dei "Sepsis Six ";
- monitoraggio fetale;
- eventuali consulenze specialistiche;
- in caso di Medico Ginecologo operante in centro Spoke lo stesso valuta l'indicazione ad eventuale trasferimento della paziente presso centro Hub.

Se non ci sono i criteri diagnostici di danno d'organo (SOFA score modificato <1) e il valore del lattato sierico è <2 mmol/l, la paziente viene posta in osservazione e durante tale periodo sono monitorati i segni/sintomi di infezione e i parametri vitali secondo l'algoritmo MEOWS. In base alla valutazione clinica e ai risultati degli esami diagnostici, il medico stabilisce il piano terapeutico più adeguato e l'eventuale necessità di consulenze specialistiche durante l'osservazione clinica.

Se c'è uno o più criteri diagnostici di danno d'organo (SOFA score modificato ≥1) oppure il valore del lattato sierico è ≥2 mmol/l si procede come nel caso di rischio alto (vedi di seguito).

RISCHIO ALTO (MEOWS>2 GIALLI O >1 ROSSO O ≥1 CRITERIO DI DANNO D'ORGANO O LATTATI >2 IN PRESENZA DI SOSPETTA O CERTA INFEZIONE).

La situazione di rischio elevato è definita dalla presenza di sospetta o certa infezione associata ad una delle seguenti condizioni:

- >2 parametri gialli e/o >1 rosso alla rilevazione dei pv secondo MEOWS,
- ≥1 criteri diagnostici di danno d'organo [SOFA score modificato ≥1],
- valore del lattato sierico è >2 mmol/l.

In tale situazione la paziente è valutata congiuntamente dal Medico Anestesista Rianimatore, dal Ginecologo e se possibile dall'Infettivologo (ove non possibile Medico Internista e/o consulto Infettivologo centro Hub). Il percorso diagnostico-terapeutico assistenziale deve prevedere quanto segue (come nel rischio intermedio):

- monitoraggio dei PV secondo algoritmo MEOWS,
- supporto delle funzioni vitali ove necessario,
- esami ematochimici "pannello sepsi",
- ricerca il danno d'organo (criteri diagnostici o SOFA score modificato),
- effettuazione dei "Sepsis Six " (Capitolo 4),
- monitoraggio fetale,
- eventuali consulenze specialistiche,
- in caso di Medico Ginecologo operante in centro Spoke, lo stesso valuta l'indicazione ad eventuale trasferimento della paziente presso centro Hub,
- considerare il ricovero in area critica.

Figura 2. Strumento di identificazione del rischio in caso di sospetta o certa infezione materna**.**

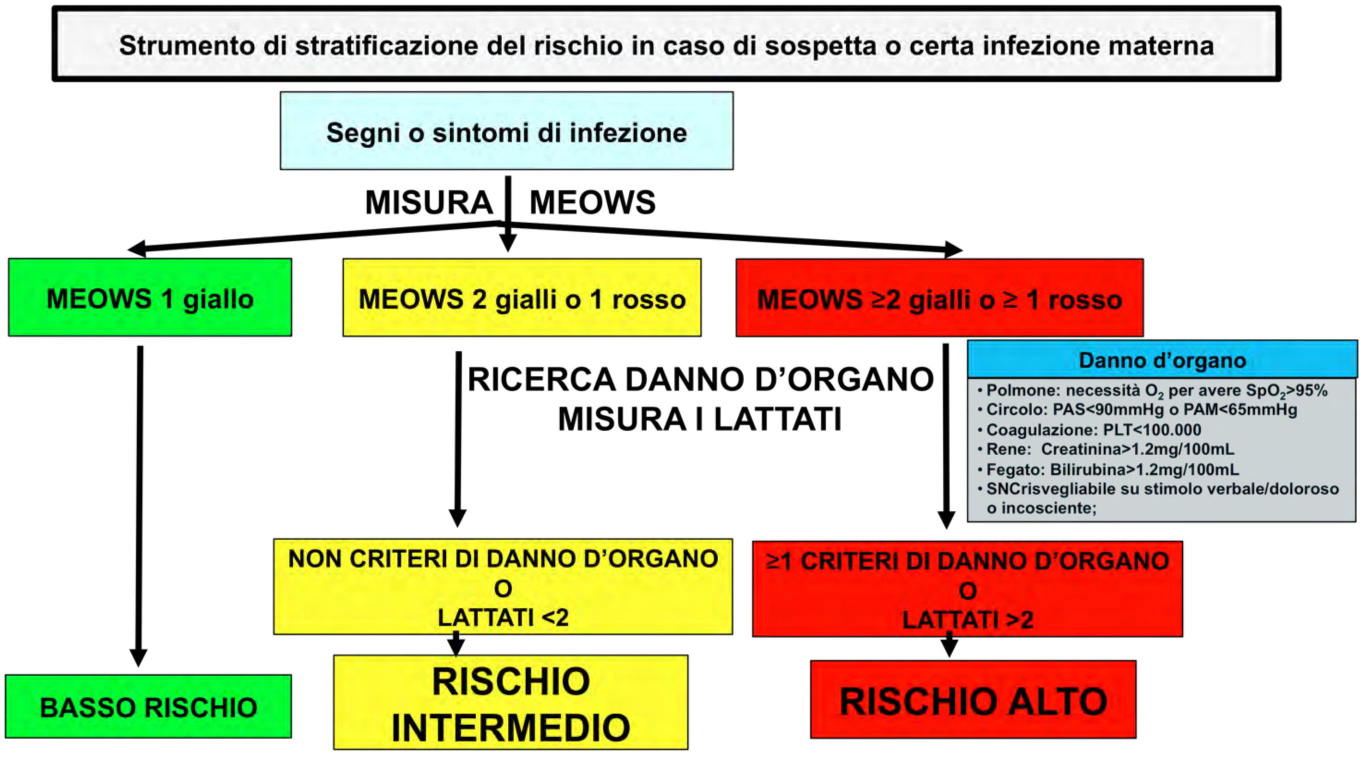

Raccomandazioni

- **Ad ogni valutazione della paziente ostetrica, sia per un nuovo accesso ospedaliero sia durante la degenza, ricercare sempre in tutti i pazienti con infezione presunta o accertata segni di danno d'organo (es. ipotensione o alterazione del sensorio etc.) e ricercare in tutti i pazienti con danno d'organo un'infezione per poter confermare o escludere la diagnosi di sepsi/shock settico; nei pazienti con alterazione dei parametri vitali secondo MEOWS (≥2 parametro giallo o ≥1 rosso), ricercare segni/sintomi di un'eventuale infezione e di un eventuale danno d'organo.**
- **Per identificare l'eventuale presenza di un'infezione in atto, effettuare un'accurata anamnesi (sintomi riferiti dalla paziente) e/o un attento esame clinico (segni/sintomi rilevati dall'operatore).**
- **L'identificazione di un eventuale danno d'organo si basa in prima istanza sulla rilevazione di uno o più parametri vitali alterati (rilevati da ≥1 parametro giallo MEOWS) e/o da un'alterazione degli esami di laboratorio (creatininemia, bilirubinemia e conteggio piastrinico). Solo se tali alterazioni dei parametri vitali e/o degli esami ematochimici corrispondono ai criteri diagnostici di danno (punteggio di SOFA score modificato ≥1) in presenza di un'infezione certa o presunta si pone la diagnosi di sepsi.**
- **in presenza sospetta o certa infezione valutare sempre la condizione di rischio in base al codice colore MEOWS.**
  - **rischio basso: sospetta o accertata infezione + 1 parametro giallo MEOWS;**
  - **rischio intermedio: sospetta o accertata infezione +2 parametri gialli o 1 rosso MEOWS; tale condizione richiede sempre la valutazione dei criteri diagnostici di danno d'organo (valutazione SOFA score modificato), la misurazione dei lattati sierici e l'effettuazione dei sepsis six;**
  - **rischio alto: sospetta o accertata infezione+ >2 parametri gialli o >1 rosso MEOWS o la presenza di ≥1 criteri di danno d'organo (SOFA score modificato ≥1) o lattati >2 mmol/l; tale condizione richiede sempre tutti gli interventi del rischio intermedio e la valutazione congiunta dell'anestesista-rianimatore, ginecologo ed ove possibile dell' infettivologo.**
- **Seguire il percorso diagnostico-terapeutico-assistenziale definito dal livello di rischio definito dal MEOWS e/o dalla presenza di uno o più criteri di danno d'organo (SOFA score modificato) e/o livello di lattati.**
- **Gli esami di laboratorio (pannello sepsi) devono essere richiesti in tutti i casi caso di rischio intermedio e rischio alto e su valutazione medica in caso di rischio basso.**

Azioni

- **Coinvolgere precocemente competenze multidisciplinari adeguate in rapporto al quadro clinico (Medico Ginecologo, Anestesista Rianimatore, Infettivologo).**
- **In caso di sepsi/shock settico presunti o accertati, valutare l'eventuale necessità di ricovero in un'area ad alta intensità di cura e/o l'eventuale trasferimento c/o struttura Hub.**
- **In caso di presunta o accertata sepsi/shock settico il Ginecologo e l'Anestesista-Rianimatore devono darne comunicazione alla Paziente (se le condizioni cliniche lo permettono) e/o al partner o al familiare più prossimo; tale comunicazione deve informare sulle condizioni cliniche e sui possibili rischi per la madre e il feto correlati alla patologia.**
- **Predisporre un sistema di identificazione e di inquadramento con algoritmo decisionale (soglie di rivalutazione, di allerta e di rischio) idoneo per la paziente ostetrica con sospetta infezione/sepsi/shock settico.**
- **Definire le modalità di azione e di intervento in base ai diversi livelli di allerta e di rischio.**
- **Predisporre una procedura condivisa con i criteri diagnostici per sepsi (danno d'organo) e shock settico nella popolazione ostetrica.**
- **Predisporre sistemi di comunicazione standardizzata tra operatori (ostetriche e medici) per la pronta e completa comunicazione dei dati relativi alla situazione del paziente (metodo Situation, Background, Assessment, Recommendation – SBAR).**
- **Predisporre un programma di formazione e di aggiornamento periodico sui sistemi di allerta, sui criteri diagnostici della sepsi/shock settico materna, sui percorsi diagnostico-terapeutici e sulla potenziale evolutività e letalità di queste condizioni cliniche per tutti i professionisti sanitari coinvolti nell'assistenza della paziente in gravidanza/puerperio.**

4. PRIMI INTERVENTI DIAGNOSTICO-TERAPEUTICI PER LA SEPSI MATERNA

Razionale

Studi clinici hanno dimostrato che nella popolazione ostetrica il trattamento tempestivo della sepsi/shock settico gioca un ruolo chiave nel migliorare la prognosi e nel ridurre la mortalità. L'utilizzo di protocolli standardizzati di diagnosi e trattamento consente un intervento precoce ed efficace.

La gestione della sepsi nelle pazienti gravide non sembra essere differente da quella della popolazione adulta fintanto che tiene conto delle alterazioni fisiologiche materne e del benessere del feto. La valutazione della vitalità fetale ha particolare rilevanza poichè l'equilibrio fra apporto e consumo di ossigeno fetale è spesso alterato in caso di sepsi materna. La stabilizzazione materna rappresenta pertanto il miglior approccio per favorire la vitalità fetale.

Il Royal College delle Ostetriche e Ginecologi approva l'utilizzo delle raccomandazioni basate sull'evidenza proposte dalla Surviving Sepsis Campaign (SSC 2016) per la gestione della sepsi materna nonostante la popolazione ostetrica non sia stata specificatamente considerata in queste linee guida. Gli interventi diagnostico-terapeutici primari ("bundle rianimatorio") proposti dalla SSC sono stati "tradotti" dalla Surviving Sepsis Organization in un approccio semplice ed efficace

denominato "Sepsis Six" (un'assonanza facile da ricordare che riassume le "Sei cose da fare nella paziente settica"). Lo schema Sepsis Six, ormai un riferimento internazionale nella gestione iniziale della sepsi, prevede l'attuazione, entro la prima ora dal riconoscimento/sospetto della sepsi, di 6 interventi, di cui: 3 diagnostici (prelievo per emocoltura, misurazione dei lattati ed altri esami di laboratorio, monitoraggio della diuresi), e 3 terapeutici (somministrazione di ossigeno, somministrazione di fluidi e antibioticoterapia) secondo lo schema riportato in Figura 9. L'approccio dei Sepsis Six ormai validato ed ampliamente utilizzato nei punti nascita dei paesi anglosassoni (NHS Foundation), tiene conto non solo degli interventi diagnostico-terapeutici di provata efficacia ma anche di una valutazione clinica della paziente simile all'approccio ABCDE. Pertanto il Gruppo di Lavoro Regionale propone l'utilizzo dei Sepsis Six per la gestione diagnostico-terapeutica di tutti i casi di sepsi materna, certa o presunta con alcune "precauzioni" relative alle peculiarità della popolazione ostetrica.

Raccomandazioni

- **Attuare tutti gli interventi dei Sepsis Six in tutti i casi di sospetta o accertata sepsi materna o shock settico.**
- **In particolare attuare i Sepsis Six in tutte le pazienti con:**
  - **RISCHIO INTERMEDIO=segni/sintomi infezione + 2 gialli o 1 rosso MEOWS;**
  - **RISCHIO ELEVATO=segni/sintomi d'infezione + >2 gialli o >1 rosso MEOWS o mSOFA≥1; L'attuazione dei Sepsis Six nella paziente a RISCHIO BASSO è da considerarsi unicamente su indicazione medica**
- **Attuare tutti gli interventi dei Sepsis Six entro la 1° ora dal sospetto diagnostico.**
- **L'attuazione dei Sepsis Six deve prevedere un coinvolgimento multi-professionale e multidisciplinare; il nucleo dei soggetti coinvolti deve comprendere in ogni caso l'Ostetrica, il Medico Ginecologo, l'Anestesista-Rianimatore e l'Infettivologo, il coinvolgimento di altre figure specialistiche deve essere valutato sul singolo caso.**

Figura 9. Sepsis Six

“SEPSIS SIX”

3 Diagnostiche:

- Prelievo per emocoltura ed altri es. colturali
- Prelievo per dosaggio dei lattati ed esami ematochimici (“pannello sepsi”)
- Monitoraggio della diuresi

3 Terapeutiche:

- Valutazione necessità O_2 e/o assistenza ventilatoria;
- Terapia antibiotica empirica
- Resuscitazione volemica

Azione

— **Predisporre una procedura operativa esplicativa sulle indicazioni, modalità e i tempi di attuazione dei Sepsis Six.**

— **Predisporre un programma di formazione e di aggiornamento periodico sui primi interventi diagnostico-terapeutici (Sepsis Six) da attuare in caso di sospetta sepsi/shock settico materna per tutti i professionisti sanitari coinvolti nell’assistenza della paziente in gravidanza/puerperio.**

PRELIEVO PER EMOCOLTURA ED ESAMI COLTURALI

Prima di iniziare il trattamento antibiotico devono essere eseguite le emocolture (almeno 2 set di colture per la ricerca di germi aerobi ed anaerobi) ed eventualmente altre colture a seconda del sospetto sito di infezione. La loro esecuzione non deve in alcun modo ritardare la somministrazione della terapia antibiotica.

Razionale

Gli esami microbiologici sono essenziali per la diagnosi eziologica e per la modulazione della terapia antibiotica empirica della sepsi. L’identificazione dell’agente patogeno favorisce una prognosi migliore nei casi di sepsi e shock settico; la strategia della de-escalation della terapia antibiotica riduce i costi e l’insorgenza di resistenze ed effetti avversi. L'emocoltura è l'elemento diagnostico essenziale per la diagnosi clinica. Tutte le colture indicate (sangue, urine, espettorato, feci, materiale da raccolte, drenaggi, dispositivi, antigeni urinari precoci, altro) devono essere raccolte prima dell'inizio della terapia antibiotica. L'esecuzione degli esami colturali non deve comportare un ritardo eccessivo (>45 min) nell'inizio della terapia antibiotica. Le modalità di raccolta, conservazione, invio ("fase preanalitica") e di lavorazione sono essenziali per garantire risultati attendibili e utili.

Raccomandazioni

- **Prima della somministrazione dell'antibiotico eseguire almeno 2 set di emocolture (2 set di colture = 4 flaconi): >1 per via percutanea e almeno 1 da ogni accesso vascolare (se in sede da >48h); se la terapia antibiotica fosse già in corso, è consigliabile l'esecuzione del prelievo prima della somministrazione successiva.**
- **Effettuare esami colturali per tutte le sedi anatomiche che potrebbero essere focolaio di partenza dell'infezione, individuate dall'esame obiettivo e dall'anamnesi della paziente (per es. urinocoltura, tampone profondo della ferita e/o prelievo del materiale purulento, tampone faringeo per Influenza, espettorato e/o Ag urinari per Legionella e Pneumococco, liquor, colturale della placenta, secrezioni vaginali ecc.; i campioni vanno raccolti, conservati ed inviati secondo le procedure locali.**
- **Gli esami colturali appropriati devono poter essere effettuati in tutte le unità di degenza (compresa Sala Parto o Sala Operatoria) ed in Pronto Soccorso (generico o ostetrico); la sede di ricovero non deve ritardare il prelievo e l'invio degli esami colturali.**

Azioni

- **Prevedere specifiche istruzioni operative per l'effettuazione degli esami colturali (emocolture ed altri esami colturali) comprese le modalità di raccolta, la conservazione, l'invio da parte dei reparti (compresa Sala Parto e Camera Operatoria), accettazione da parte del Laboratorio, elaborazione e comunicazione del risultato e dei valori critici da parte del laboratorio che deve essere garantita anche nel caso di un successivo trasferimento della paziente in altro reparto.**
- **Predisporre procedure operative per l'elaborazione di esami colturali in urgenza (ove necessario e/o ove garantito 7/7) delle emocolture e delle altre colture.**
- **Valutare (ove possibile) l'utilizzo di tecnologie validate che riducano i tempi di risposta.**

LATTATI, EMOGLOBINA E PARAMETRI DI FUNZIONALITA' D'ORGANO

Razionale

Nella popolazione settica la valutazione precoce del valore ematico dei lattati gioca un ruolo chiave nella stratificazione del rischio e nella gestione terapeutica. L'aumento del valore del lattato, indice del metabolismo anaerobio nei casi di sepsi spesso secondario ad ipoperfusione tissutale, si associa ad un rischio di deterioramento clinico ed ad una prognosi infausta. Nella popolazione ostetrica in presenza di segni/sintomi di infezione un valore del lattato sierico >2 mmol/l definisce una paziente ad alto rischio. Secondo le definizioni un valore di lattato >2 mmol/L in associazione ad una grave ipotensione arteriosa refrattaria al carico volemico, con necessità di supporto aminico per mantenere MAP≥65 mmHg sancisce la diagnosi di shock settico materno. Il valore di lattato, non solo come valore assoluto ma anche come trend, è importante per la valutazione della risposta alla

terapia, in particolare alla resuscitazione volemica come dimostrato dall'associazione fra la clearence dei lattati e l'efficacia della fase resuscitativa nella popolazione settica.
La valutazione dei parametri di funzionalità d'organo è fondamentale per evidenziare un danno d'organo non clinicamente visibile e permette la valutazione del SOFA score modificato e quindi di formulare la diagnosi di sepsi.
La misura del livello di emoglobina permette l'identificazione di un eventuale quadro di anemia che in caso di sepsi potrebbe peggiorare l'ipoperfusione per un ridotto trasporto di ossigeno ai tessuti.

Raccomandazioni

— **Misurare il valore ematico dei lattati in tutte le pazienti ostetriche con segni e/o sintomi di sospetta o presunta infezione; in particolare la misura dei lattati deve essere effettuata in tutte le pazienti ostetriche con rischio intermedio (=segni/sintomi infezione + 2 gialli o 1 rosso MEOWS) e rischio alto (=segni/sintomi d'infezione +>2 gialli o >1 rosso MEOWS o mSOFA≥1).**

— **Misurare il valore ematico dei lattati tramite prelievo arterioso o venoso da accesso periferico o centrale mediante una metodica point of care per ottenere un risultato nel più breve tempo possibile (<30 min).**

— **Misurare i parametri di funzionalità d'organo ("pannello sepsi": emocromo completo con formula, lattati, elettroliti, azotemia, creatininemia, bilirubinemia, PT-PTT, PCR o PCT) per evidenziare un eventuale danno d'organo non visualizzabile all'esame obiettivo e per permettere la formulazione della diagnosi mediante il calcolo del SOFA score modificato.**

— **La trasfusione di emazie concentrate in caso di sepsi/shock settico (in assenza di altre patologie quali ischemia miocardica, ipossiemia severa o emorragia massiva) è consigliata quando il livello di Hb scende < 7 g/dL.**

Azioni

— **Predisporre una procedura operativa per la misura point of care dei lattati in tutti i punti nascita.**

— **Predisporre una procedura operativA per la richiesta di esami di laboratorio in urgenza 24/7 (comprese modalità di raccolta, invio, comunicazione e registrazione del risultato) e ove possibile predisporre un "pacchetto" di esami specifici per la sepsi ("pannello sepsi") per facilitarne la richiesta.**

MONITORAGGIO DIURESI

Razionale

La diuresi oraria è una misura sensibile della perfusione renale per cui un'oliguria/anuria con valori di diuresi < 0.5 ml/kg/h può indicare un'alterazione della perfusione renale secondaria ad una compromissione emodinamica. Un' oliguria o anuria possono essere il primo segnale di un danno renale sepsi correlato confermabile mediante gli esami di funzionalità renale.

Raccomandazioni

- **Monitorare la diuresi in tutte le pazienti ostetriche con sospetta o accertata infezione/sepsi/shock settico.**
- **Posizionare il catetere vescicale in tutte le pazienti ostetriche con un quadro di sepsi o shock settico per il monitoraggio della diuresi oraria.**

OSSIGENOTERAPIA

Razionale

La fisiopatologia della sepsi è caratterizzata da un'aumentata richiesta metabolica di ossigeno ma da una sua ridotta disponibilità e capacità di utilizzo a livello periferico per riduzione del trasporto e dell'estrazione. La somministrazione di ossigeno supplementare contribuisce a migliorare il trasporto di ossigeno, correggendo l'eventuale desaturazione arteriosa. L'obiettivo del trattamento è di avere una saturazione arteriosa in O2>94%, valore soglia per prevenire un'ipossia fetale con gravi conseguenze.

Raccomandazioni

- **Si raccomanda la valutazione e il monitoraggio della funzione respiratoria (frequenza respiratoria e SpO2) in tutte le pazienti con sospetta o accertata sepsi.**
- **Si raccomanda la somministrazione di ossigenoterapia (umidificata) con FiO_2 100% con maschera facciale con reservoir, dotata di valvola non–rebreathing nelle pazienti ostetriche con sospetta o accertata sepsi.**

Azione

- **Predisposizione di una procedura operativa per la somministrazione dell'ossigenoterapia e per il monitoraggio della funzione respiratoria.**

TERAPIA ANTIBIOTICA

Razionale

La somministrazione tempestiva di un'appropriata terapia antibiotica entro la prima ora dall'identificazione della sepsi e dopo il prelievo di appropriate colture è essenziale per un efficace trattamento: ogni ora di ritardo si associa ad un significativo incremento della mortalità. La terapia antibiotica iniziale empirica si basa su criteri clinici ed epidemiologici ed usualmente include uno o più farmaci ad ampio spettro di azione, attivi contro i possibili patogeni (batteri e/o funghi), ai dosaggi efficaci e con caratteristiche che garantiscono la penetrazione nei focolai di infezione presenti. In particolare la scelta dello schema antibiotico deve tener conto dei seguenti elementi:
La principale fonte settica in ambito ostetrico è il tratto genitale, seguito dalle vie urinarie e dalle ferite (Tabella 11, allegato 4); da studi internazionali i microrganismi più frequentemente isolati in ambito ostetrico sono l'E.Coli, lo Streptococco di Gruppo B ed i germi anaerobi; spesso sono presenti infezioni miste (Gram positivi e negativi) (Tabella 12, allegato 4). In Italia il tasso di resistenze ai carbapenemi negli enterobatteri, per diffusione di carbapenemasi e produzione di *ß-lattamasi a spettro esteso* (ESBL), è superiore rispetto alla media europea e con un trend in aumento negli ultimi anni. Considerando l'elevata mortalità associata ad un trattamento iniziale non appropriato (in letteratura non ostetrica è stata stimata una riduzione della sopravvivenza fino a 5 volte se la terapia antibiotica empirica in caso di shock settico non copriva il patogeno in causa), si ritiene preferibile che lo schema terapeutico prescelto sbagli nel senso della "sovra-inclusività". Al fine di facilitare la scelta della terapia antibiotica empirica più appropriata, si riporta nella Figura 10 lo spettro d'azione degli antibiotici più comunemente utilizzati per il trattamento delle pazienti con sepsi/shock settico.

Figura 10. Spettro d'azione dei principali antibiotici utilizzati nel trattamento della sepsi in ostetricia.

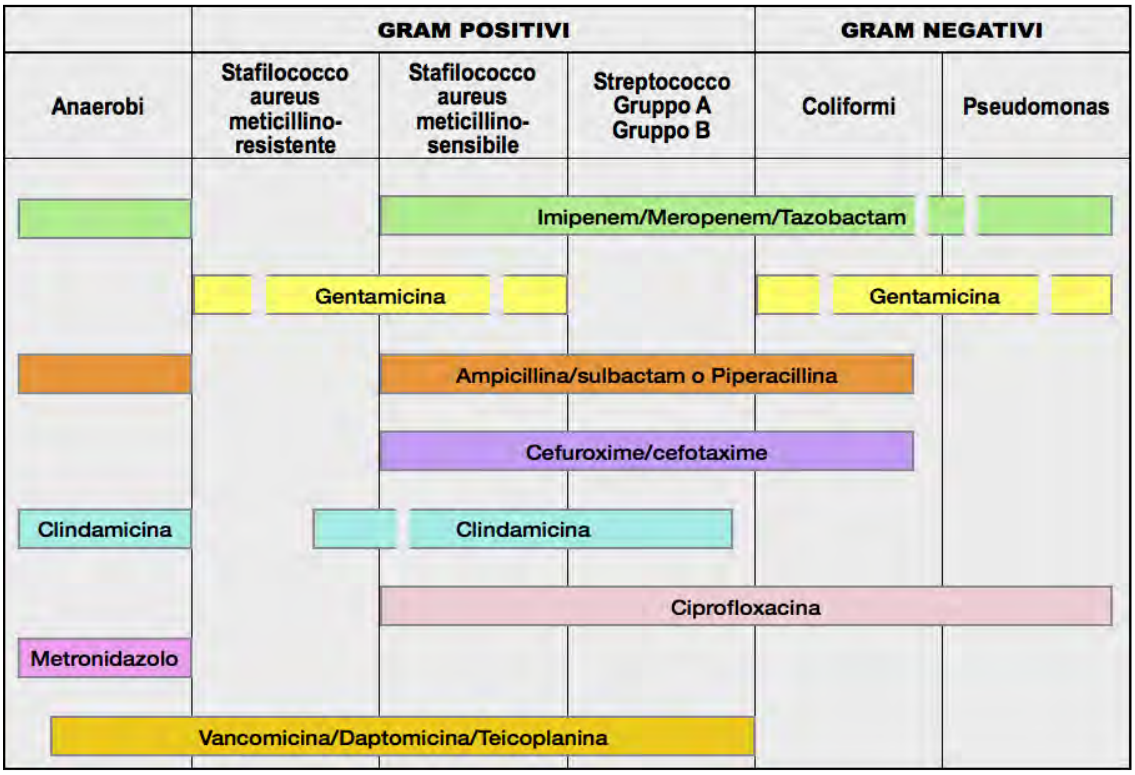

Modificato da Royal College of Obstetricians and Gynaecologists (RCOG) Sepsis in Pregnancy, Bacterial (Green-top Guideline No. 64a), 2012

Nella Tabella 13 (allegato 4) sono riportati i dosaggi standard per la somministrazione endovenosa dei diversi farmaci utilizzati nella terapia antibiotica empirica della sepsi/shock settico. Si ricorda l'importanza di somministrare sempre la dose carico e la dose di mantenimento appropriata per ottenere le concentrazioni plasmatiche efficaci, poiché il fallimento nel raggiungere le concentrazioni di picco plasmatico è associato al rischio di insuccesso clinico.
Nella Tabella 7 sono riportate alcune proposte di schemi di trattamento antibiotico empirico della sepsi e shock settico nella paziente ostetrica. Si rimanda all'Allegato 4 per i dettagli relativi alla scelta del regime antibiotico, alla sua durata e modulazione.

Tabella 7. Regimi antibiotici da considerare per il trattamento antibiotico empirico della sepsi/shock settico nella paziente ostetrica con focolaio a partenza dal tratto genitale, dalle vie urinarie o dalla ferita.

ANTIBIOTICO	DOSE	NOTE
**Piperacillina/tazobactam +**<br>**Gentamicina *oppure***<br>**Amikacina**	- 4,5 g ogni 6 h<br>- 5 mg/kg/die in unica somministrazione<br>- 15mg/kg/die in unica somministrazione	
**Meropenem +**<br>**Gentamicina *oppure***<br>**Amikacina**	- 1 g ogni 8 h<br>- 5 mg/kg/die in unica somministrazione<br>- 15mg/kg/die in unica somministrazione	Solo nel caso di recente infezione da batteri ESBL, la condizione di colonizzazione da batteri ESBL o la recente esposizione fluorochinoloni e/o cefalosporine
**Ciprofloxacina +**<br>**Gentamicina ±**<br>**Metronidazolo (per anaerobi)**	- 400 mg ogni 8-12 h<br>- 5 mg/kg/die in unica somministrazione<br>- 1 g dose di carico, poi 500 mg ogni 6 h	Se allergia *grave* a penicillina (anafilassi: broncospasmo, angioedema, ipotensione)
**Piperacillina/tazobactam +**<br>**Gentamicina +**<br>**Daptomicina *oppure***<br>**Vancomicina**	- 4,5 g ogni 6 h<br>- 5 mg/kg/die in unica somministrazione<br>- 8 mg/kg/die in unica somministrazione<br>- 25 mg/kg dose di carico, poi 500 mg ogni 6 h	Se sospetta infezione da MRSA (vedi testo)

Raccomandazioni

- **Somministrare il trattamento antibiotico per via endovenosa non appena possibile e comunque entro la prima ora in tutti i casi di sospetta o accertata sepsi/shock settico e più nello specifico nelle pazienti a rischio intermedio e alto.**
- **Il trattamento empirico deve essere iniziato con uno o più antibiotici ad ampio spettro che possano coprire i patogeni più probabilmente coinvolti nell'eziologia della sepsi o dello shock settico.**
- **Una volta identificato il patogeno responsabile e ottenuto l'antibiogramma e/o documentato un miglioramento clinico, occorre modulare il trattamento antibiotico.**

Azioni

- **Predisporre protocolli locali di terapia antibiotica empirica con specifica di dosaggi, modalità di somministrazione, durata e rimodulazione (de-escalation).**
- **Verificare la pronta disponibilità o la reperibilità degli antibiotici nelle possibili sedi di utilizzo 24/7.**
- **Predisporre le procedure di consulenza dell'Infettivologo (guardia attiva o reperibilità) ed ove non possibile Medico Internista e/o consulenza Infettivologo centro Hub.**

CONTROLLO DEL FOCOLAIO SETTICO

Razionale

Il controllo del focolaio settico, con l'eradicazione dello stesso dove indicato, è indispensabile per il successo del trattamento della sepsi e shock settico. Pertanto è necessario identificare prontamente il focolaio settico mediante l'esame obbiettivo e la diagnostica di imaging più idonea in relazione al sospetto clinico. Le diverse fasi della gravidanza e del puerperio possono anch'esse indirizzare verso il possibile focolaio settico e suggerire il corretto percorso diagnostico-terapeutico; a titolo esemplificativo: in tutti i casi di sepsi in puerperio è fondamentale eseguire un'ecografia pelvica per identificare raccolte o presenza di materiale in cavità, nelle pazienti con diagnosi di endometrite escludere la presenza di ascessi pelvici o microascessi uterini, la diagnosi di vulvite necrotizzante rappresenta un' emergenza chirurgica etc. Le principali procedure per il controllo del focolaio settico in ambito ostetrico sono riportate nella Tabella 8.

Tabella 8. Principali procedure per il controllo del focolaio settico in ambito ostetrico.

1) evacuazione di prodotti del concepimento ritenuti
2) risanamento del letto della ferita (debridement) nelle infezioni di ferita e nelle infezioni dei tessuti molli
3) drenaggio di ascessi
4) stent o nefrostomia percutanea per le pielonefriti ostruttive
5) espletamento del parto in caso di corionamniosite
6) isterectomia se necrosi miometriale

Raccomandazioni

- **Identificare e controllare la fonte settica con l'eradicazione della stessa dove indicato, entro 6-12 ore dalla diagnosi di sepsi/shock settico.**
- **Ottenere l'eradicazione di un focolaio settico usando la tecnica di controllo meno invasiva e più efficace in rapporto ai rischi e benefici (drenaggio, sbrigliamento, rimozione dei dispositivi potenzialmente infetti) in particolare nelle pazienti in gravidanza.**
- **In caso di eradicazione di un focolaio settico, prelevare campioni del materiale biologico infetto (liquidi biologici o biopsie tissutali) e inviarlo per esame colturale.**
- **Considerare sempre possibili focolai settici non ostetrici, (per es. appendicite, ascesso pancreatico, infarto intestinale, influenza).**
- **Rimuovere prontamente gli accessi vascolari ritenuti possibili focolai settici, non appena siano stati posizionati altri accessi vascolari.**
- **Valutare la decisione di espletare il parto nell'ambito della sepsi ostetrica bilanciando i rischi legati all'epoca gestazionale, le condizioni materne e quelle fetali. La stabilizzazione delle condizioni materne è prioritaria prima di espletare il parto per compromissione del benessere fetale, poiché l'intervento sulla madre potrebbe migliorare la condizione del feto.**

Azioni

- **Elaborare percorsi diagnostici idonei all'identificazione del focolaio settico (con particolare attenzione alla diagnostica per immagini in corso di gravidanza) e alla formulazione degli interventi di trattamento.**
- **Definire procedure relative all'assistenza alla paziente durante le diverse fasi della diagnostica.**
- **Elaborare protocolli multidisciplinari per il trattamento specialistico finalizzato all'eradicazione del focolaio settico (chirurgia, radiologia, radiologia interventistica).**
- **Verificare le effettive possibilità operative delle competenze specialistiche con particolare riferimento alla radiologia interventistica e alla disponibilità di sala operatoria e dell'equipe necessaria disponibili in urgenza sull'arco delle 24 ore e definire possibilità alternative (eventuale trasferimento in un centro Hub).**
- **Verificare le modalità di attivazione della equipe multidisciplinare.**

RESUSCITAZIONE VOLEMICA

Razionale

Il quadro fisiopatologico della sepsi è caratterizzato da alterazioni della perfusione tissutale nella quale l'ipovolemia (assoluta e/o relativa) svolge un ruolo essenziale. Il tempestivo reintegro

volemico pùo correggere l’ipovolemia e spesso lo stato ipotensivo e migliorare l’ipoperfusione pericolosa per la mamma ed il feto.
L’obbiettivo della resuscitazione volemica è quello di ottenere una PAM≥65mmHg, tale valore va considerato in relazione ai parametri pressori abituali della paziente e agli altri indici di perfusione (diuresi e lattati). Una clearence dei lattati pari al 10% in 2 ore si è dimostrato uno strumento semplice ed adeguato per valutare l’efficacia della resuscitazione volemica e dell’evoluzione del quadro clinico.
Bisogna ricordare che in alcuni casi la resuscitazione volemica, a causa dello stato di permeabilizzazione tipico della sepsi, può essere responsabile di un edema polmonare lesionale con conseguente distress respiratorio. Tale “complicanza” non rappresenta un errore clinico ma il risultato di un conflitto d’organo (cuore-polmone) che dovrà essere fronteggiato con la sospensione/riduzione del reintegro volemico e con un’assistenza respiratoria.
Una condizione di ipotensione con necessità di somministare farmaci vasopressori per mantenere una PAM ≥65mmHg e una lattacidemia >2mmol/L dopo adeguata resuscitazione volemica definiscono un quadro di shock settico; per adeguata resuscitazione volemica s’intende la somministrazione di 30ml/kg di cristalloidi in boli refratti di 500 ml in 1 ora. Per definire un paziente in shock settico è indispensabile valutare la risposta al carico volemico: se l’ipotensione arteriosa associata ad un valore di lattati >2mmol/L persiste, la diagnosi è di shock settico, se invece si risolve la diagnosi è di sepsi.
Bisogna considerare che le alterazioni del sistema cardiovascolare tipiche della paziente gravida hanno un significativo impatto sull’inquadramento clinico e sulla gestione emodinamica in caso di shock: 1) in gravidanza il volume ematico aumenta fino al 50% , per i segni clinici di ipovolemia si evidenziano tardivamente, solo dopo una perdita del 30% del volume ematico; 2) in caso di ipotensione materna la placenta è l’organo che risponde con maggior vasocostrizione con secondaria ipossia fetale ed acidosi; 3) il distress fetale può essere il primo segno di instabilità emodinamica; 4) dalla 20° settimana l’utero gravidico può causare compressione aorto-cavale riducendo il ritorno venoso al cuore con conseguente ipotensione.
Una gestione emodinamica tempestiva ed “attenta” alle peculiarità della paziente gravida è fondamentale per migliorare la prognosi in questa popolazione.

Raccomandazioni

- **Nelle pazienti con ipotensione in caso di sospetta o accertata sepsi/shock settico, effettuare un carico volemico di 30ml/kg di cristalloidi in boli refratti di 500 ml entro la 1° ora; si raccomanda dopo la 20° settimana di gravidanza di posizionare la paziente in decubito laterale sinistro per escludere la compressione aorto-cavale come causa/concausa di ipotensione arteriosa.**
- **Effettuare la resuscitazione volemica con cristalloidi alternando soluzione fisiologica con soluzione elettrolitiche bilanciate; si suggerisce utilizzo albumina in aggiunta ai cristalloidi nelle pazienti che abbiano necessità di elevata quantità di fluidi.**
- **Monitorare i seguenti parametri vitali in corso di resuscitazione volemica: pressione arteriosa, frequenza cardiaca, diuresi oraria, SpO_2, frequenza respiratoria.**
- **Valutare la risposta alla resuscitazione volemica sia in termini di efficacia (miglioramento PA, perfusione periferica: diuresi e lattati) che in termini di complicanze.**
- **Proseguire la resuscitazione volemica fino al raggiungimento dell’obbiettivo di una PAM ≥65mmHg per una dose massima di 30ml/kg; dosi aggiuntive di fluide devono essere eventualmente prescritte dal Medico anestesista Rianimatore.**

- **Monitorare il livello ematico dei lattati in tutti i casi di ipoperfusione e shock conclamato per valutare la risposta alla resuscitazione volemica e l'evoluzione del quadro clinico.**
- **Valutare la vitalità fetale in tutti i casi d'ipoperfusione o di shock conclamato.**

Azioni

- **Predisposizione di un protocollo di "resuscitazione volemica" con indicazione della tipologia, della quantità e delle modalità di somministrazione dei fluidi, del monitoraggio materno e fetale e degli obiettivi che si intendono raggiungere.**
- **Verifica della disponibilità di sistemi infusionali adeguati (pompe infusionali).**
- **Verifica della disponibilità delle soluzioni indicate (quantità, qualità) nelle sedi di utilizzo (sala parto, camera operatoria ecc).**
- **Verificare procedura di richiesta di competenza specialistica (Anestesista- Rianimatore).**

PROFILASSI DEL TROMBOEMBOLISMO VENOSO

Razionale

La sepsi in ambito ostetrico è considerata un fattore di rischio per tromboembolismo venoso: i meccanismi patogenetici sottostanti non sono completamente chiariti, ma si ritiene siano la conseguenza di multipli fattori quali immobilità, attivazione della cascata infiammatoria, coagulazione intravascolare disseminata e stasi venosa.
Dosi profilattiche di LMWH nella paziente ostetrica (gravidanza e puerperio) sono quelle definite nelle linee guida dell'American College of Chest Physicians (2012) come segue: deltaparina 5000 UI s.c. ogni 24 ore; nadroparina 2850 UI s.c. ogni 24 ore; enoxaparina s.c. 40 IU s.c. ogni 24 ore. Agli estremi di peso corporeo, il dosaggio deve essere aggiustato.

Raccomandazioni

- **Nella paziente settica è raccomandata la profilassi farmacologica del tromboembolismo venoso con eparina a basso peso molecolare (LMWH), salvo controindicazioni all'utilizzo di questo farmaco.**
- **Qualora possibile, la profilassi farmacologica andrebbe associata a quella meccanica (calze elastiche o compressione pneumatica intermittente). Utilizzare solamente la profilassi con mezzi meccanici solo se vi sono controindicazioni cliniche alla profilassi con LMWH.**

Azione

- **Predisporre protocolli per la profilassi tromboembolica nella gravida e nella puerpera con sepsi.**

ALLEGATI

ALLEGATO 1. I PARAMETRI DELLE SCHEDE MEOWS

Ad ogni valutazione devono essere rilevati come parametri base:

- frequenza respiratoria,
- temperatura,
- frequenza cardiaca (polso materno),
- pressione arteriosa,
- livello di coscienza (AVPU score).

Altri parametri utili:

1. saturazione di O2,
2. diuresi,
3. aspetto della donna,
4. scala del dolore,
5. lochiazioni.

FREQUENZA RESPIRATORIA

La frequenza respiratoria rappresenta uno dei parametri più significativi essendo il primo e più sensibile indicatore del deterioramento delle condizioni cliniche della paziente (Johnson and Taylor, 2010). Questo parametro si misura contando gli atti respiratori per 30 secondi e quindi raddoppiando, se la frequenza del respiro è costante, altrimenti si prosegue a contare fino ai 60 secondi. Un altro metodo di misurazione consiste nell'appoggiare il polso della donna sul suo torace e rilevare le escursioni toraciche.

Raccomandazione

- **La frequenza respiratoria deve essere rilevata ad ogni valutazione subito dopo la frequenza cardiaca.**
- **E' importante che la donna non percepisca che si sta rilevando la sua frequenza respiratoria, altrimenti potrebbe modificare il proprio modo di respirare, determinando una lettura non esatta.**
- **La valutazione del sistema respiratorio deve comprendere anche l'esame obbiettivo (auscultazione).**
- **I valori di frequenza respiratoria considerati normali nelle schede MEOWS sono compresi tra 10 e 19 atti respiratori/minuto.**
- **La tachipnea rappresenta uno dei segnali più precoci e importanti in caso di sepsi e la sua presenza richiede sempre una valutazione clinica approfondita.**
- **La dispnea è un sintomo piuttosto comune in gravidanza, si può manifestare in qualsiasi trimestre, ha un esordio graduale ed è solitamente notato dalla donna quando parla o quando**

si corica. Le principali cause di dispnea in caso di infezione/sepsi/shock settico nella popolazione ostetrica sono riportate in Tabella 9.

Tabella 9. Principali cause di dispnea in gravidanza in caso di infezione/sepsi/shock settico

Condizione	Segni	Sintomi	Indagini	Trattamento
Polmonite	Tachipnea, dispnea, febbre, desaturazione	Tosse, febbre, malessere	Rx torace, TAC torace EGA Esami ematochimici specifici	Ossigeno<br><br>Antibiotici<br><br>Supporti respiratori
Edema polmonare lesionale	Tachipnea, dispnea<br><br>Desaturazione<br><br>Scompenso cardiaco	Ortopnea, dispnea espettorato schiumoso, rosato, dolore toracico	Rx torace, EGA, ECG ecocardiogramma Monitoraggio invasivo	Ossigeno Diuretici Amine vasoattive Trasferimento in reparto intensivo

SATURAZIONE DI OSSIGENO

I livelli di percentuale di saturazione di ossigeno dell'emoglobina arteriosa dipendono dall'efficienza degli scambi respiratori a livello polmonare e sono rilevabili dalla SpO_2.

Raccomandazioni

— **La SpO_2 può non essere misurata di routine (rientra nei possibili parametri aggiuntivi) ma deve sicuramente essere rilevata in determinate circostanze:**
  - **se la frequenza respiratoria (FR) rientra nei parametri di anormalità (rosso o giallo),**
  - **se vi è una condizione medica/ostetrica che potrebbe alterare gli scambi respiratori (es. disordini respiratori, assistenza in terapia intensiva).**

— **L'accuratezza nella valutazione della saturazione di ossigeno dipende dal flusso ematico periferico nella zona di applicazione del sensore. Se la circolazione periferica è compromessa o le condizioni cliniche della donna sono critiche, la rilevazione della SpO_2 può risultare inaccurata o non rilevabile.**

— **I parametri di saturazione normali nelle MEOWS sono compresi tra 96 e 100%.**

TEMPERATURA

L'assenza di febbre non esclude la sepsi, dal momento che la somministrazione di paracetamolo o di altri antipiretici possono ridurla. Al contrario, "l'assenza di iperpiressia nella sepsi deve essere motivo di allerta".

Raccomandazioni

- **La temperatura dovrebbe essere rilevata nella sede più adeguata (timpanica).**
- **Riportare la temperatura rilevata nella scheda MEOWS.**
- **I parametri di temperatura considerati normali nelle MEOWS sono compresi tra 36 e 37.4°C.**
- **L'ipotermia può essere un reperto significativo indicativo di infezione e non deve essere ignorato.**
- **L'iperpiressia potrebbe essere mascherata dalla somministrazione di antipiretici.**
- **In caso di iperpiressia considerare lo screening per la sepsi e la terapia antibiotica specifica precocemente.**

FREQUENZA CARDIACA

La sede più frequentemente utilizzata per la rilevazione della frequenza cardiaca è l'arteria radiale per la facile accessibilità. L'arteria brachiale viene utilizzata nella misurazione della pressione arteriosa e la carotidea e femorale possono essere valutate in caso di ipotensione, quando la gittata cardiaca non può essere rilevata nella circolazione periferica (Johnson & Taylor, 2010).

L'arteria radiale dovrebbe essere palpata utilizzando il dito indice e medio, appoggiando il polso della donna sul suo torace, e la frequenza cardiaca dovrebbe essere valutata per 30 secondi per poi raddoppiare il valore se regolare, o per 60 secondi se irregolare (Kozier et al, 1998).

Alcuni strumenti (Saturimetro, Monitor ECG) forniscono la lettura della frequenza cardiaca.

Raccomandazioni

- **La frequenza cardiaca dovrebbe essere valutata per 30 secondi per poi raddoppiare il valore se regolare, o per 60 secondi se irregolare (Kozier et al, 1998).**
- **La frequenza cardiaca può essere misurata a che con strumenti elettronici (saturimetro, monitor ECG).**
- **E' necessario riportare la frequenza cardiaca nella scheda MEOWS.**
- **I parametri considerati normali della frequenza cardiaca sono compresi tra 60 e 99 bpm.**

PRESSIONE ARTERIOSA

I valori di pressione arteriosa sistolica e diastolica vengono registrati separatamente nella tabella MEOWS. Importante, al fine di ottenere delle misurazioni corrette della pressione arteriosa, utilizzare sfingomanometri idonei alla circonferenza del braccio della paziente. La rilevazione elettronica della PA è ormai ampiamente utilizzata ed attendibile; è consigliato comunque in caso di dubbio procedere anche alla misurazione manuale.

Raccomandazioni

— **La pressione arteriosa sistolica (PAS) e diastolica (PAD) vengono riportate separatamente per facilitare la rilevazione di valori di allerta.**

— **La pressione deve essere rilevata utilizzando uno sfigmomanometro adeguato alle dimensioni del braccio della donna.**

— **La pressione arteriosa sistolica dovrebbe essere rilevata al I suono di Korotkoff chiaramente udibile, mentre la pressione diastolica al V Korotkoff, quando il suono non è più rilevabile.**

— **La rilevazione elettronica della PA può essere imprecisa nella lettura. In caso di alterazioni importanti è buona norma rivalutarla mediante misurazione manuale.**

— **I valori di PAS considerati normali nella scheda MEOWS sono compresi tra 100 e 139 mmHg, e tra 50 e 89 per la PAD.**

L'ipotensione costituisce un segno tardivo di deterioramento delle condizioni cliniche nella paziente gravida. L'ipotensione fisiologica in gravidanza e nel puerperio possono determinare un ritardo nel riconoscimento precoce di una possibile alterazione emodinamica.

Ipertensione – la definizione convenzionale di ipertensione in gravidanza è:

- Due rilevazioni di 140/90 mmHg a distanza di almeno 4 ore (NCCWCH, 2010). Le linee guida dell'AIPE adottano la definizione di ipertensione gestazionale in presenza di valori di pressione arteriosa sistolica >140 mmHg e/o di pressione diastolica >90 mmHg, in almeno due misurazioni consecutive, a distanza di almeno 6 ore l'una dall'altra, dopo la 20a settimana di gravidanza, in una donna normotesa prima della gravidanza e prima della 20a settimana (Report National High Blood Pressare, 2000).
- Un aumento di 15 mmHg al di sopra dei valori pressori rilevati alla prima visita
- Una misurazione di 160/100 o maggiore.

DIURESI

Proteinuria: può indicare presenza di infezione, patologia renale sottostante risultante dall'ipertensione o può essere un campione contaminato (da liquido o secrezioni vaginali). Test positivi transitori in genere non hanno significato e sono dovuti ai cambiamenti fisiologici della gravidanza con comparsa di minime quantità di albumina o globulina nelle urine. Per escludere la presenza di infezioni è necessario inviare un campione per urinocoltura.

Glicosuria: è un reperto comune in gravidanza per i cambiamenti fisiologici che avvengono della funzionalità renale. Tuttavia il glucosio può apparire nelle urine anche in caso di:

- aumento dei livelli di glucosio (iperglicemia)
- modificazioni della funzionalità e quindi dell'assorbimento renale
- transitorio dopo la somministrazione di corticosteroidi es. Betametasone/Desametasone.

Raccomandazioni

— **L'esame delle urine mediante stick urinario dovrebbe essere eseguito e documentato nella scheda MEOWS nelle seguenti occasioni:**

- **In presenza di disordini materni specifici quali ipertensione, diabete o segni/sintomi tipici delle complicanze ipertensive della gravidanza, anche in assenza di ipertensione arteriosa.**
- **In presenza di sintomi clinici quali disuria.**

— **La frequenza dell'esame delle urine dopo il ricovero dipende dalla valutazione clinica e dalla diagnosi formulata.**

VALUTAZIONE DELLO STATO NEUROLOGICO – SCALA AVPU

La risposta neurologica rappresentala una misura dello stato di coscienza. La scala AVPU indica:

- **A** – vigile ed orientato verso persone, luogo, tempo ed evento (Alert).
- **V** – risponde alla voce/stimoli verbali (per esempio nel postoperatorio) (Verbal).
- **P** – risponde a stimoli dolorosi con movimenti volontari o involontari (Pain).
- **U** – non responsivo – il paziente non risponde ad alcun tipo di stimolo (Unresponsive).

Raccomandazioni

— **La valutazione neurologica dovrebbe essere riportata nella scheda MEOWS.**

- Vigile (A): box bianco.
- Risposta alla voce (V): box giallo.
- Risposta al dolore (P): box rosso.
- Nessuna risposta (U): box rosso.

— **Qualsiasi cambiamento nel livello di coscienza (scala AVPU) deve sempre essere considerato significativo e si deve agire immediatamente.**

VALUTAZIONE DEL DOLORE

Un dato importante che deve sempre essere incluso nell'inquadramento clinico complessivo della donna è la valutazione del dolore *mediante una scala di punteggio da 0-10 (o: nessun dolore, 10: dolore estremo).* Essendo questo dato caratterizzato da una importante componente soggettiva, non viene inserito con uno score nella scheda MEOWS, ma si ricorda che deve sempre essere considerato.

Nel documento irlandese di riferimento, vengono proposte altre scale di valutazione del dolore che possono essere utilizzate. In Figura 11 riportiamo la scala di valutazione del dolore proposta dal panel.

Figura 11. Scala di valutazione del dolore

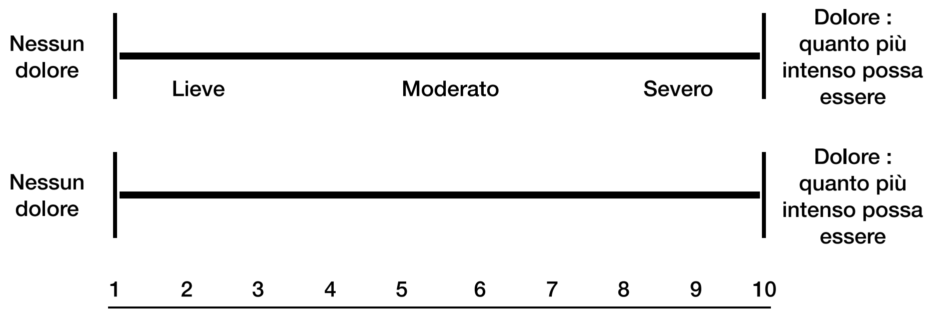

Raccomandazione

— **Non vi è un punteggio assegnato alla scala di dolore, ma qualsiasi elemento di preoccupazione in relazione al dolore riferito dalla donna deve essere motivo di coinvolgimento dell'Ostetrica in turno, del ginecologo o dell'anestesista.**

ALLEGATO 2. INTENSIFICAZIONE DI MONITORAGGIO

- Intensificare la frequenza delle rilevazioni ogni 15 minuti,
- monitorare saturazione O2 e somministrare O2 con maschera facciale,
- se in epoca prenatale, posizionare la donna in decubito laterale sinistro a 15-20° e iniziare, monitoraggio cardiotocografico (CTG) una volta stabilizzata la donna,
- valutare l'accesso venoso,
- valutare foglio terapia e la corretta somministrazione dei farmaci, riportare eventuale ritardo di somministrazione, soprattutto di farmaci antipertensivi,
- assicurarsi di avere personale ostetrico esperto disponibile e valutare il luogo più idoneo per gestire la situazione,
- organizzare l'eventuale trasferimento,
- valutare la posizione migliore del letto (se in posizione litotomica o seduto),
- verificare che l'ambiente sia sicuro,
- verificare l'invio dei campioni di laboratorio / la ricezione degli esami,
- portare apparecchio per monitoraggio, siringhe per emogasanalisi e dispositivi per indagini ulteriori (es. colturali),
- riportare in cartella in maniera dettagliata tutto quanto viene effettuato,
- chiedere alla paziente di riferire qualsiasi sintomo / segno di peggioramento delle condizioni cliniche ed assicurarsi di ottenere una documentazione adeguata,
- informare la paziente ed i suoi famigliari in merito al piano assistenziale.

ALLEGATO 3. IMPLEMENTAZIONE E OSTACOLI ALL'UTILIZZO DELLA SCHEDA MEOWS

L'elevato carico di lavoro, dovuto alla cronica carenza di personale e l'aumentata complessità della popolazione ostetrica, possono entrare in conflitto con l'applicazione del monitoraggio previsto dalla scheda MEOWS; la rilevazione dei parametri potrebbe essere giudicata da alcuni "eccessiva" e non necessaria in una condizione fisiologica come la gravidanza.
Per ottenere una migliore compliance da parte del personale nei confronti della nuova procedura di monitoraggio è sicuramente necessario tenere in considerazione e implementare anche gli aspetti organizzativi e la formazione adeguata di tutte le figure professionali coinvolte nell'assistenza alla donna gravida.
I problemi rilevati nell'utilizzo delle schede MEOWS sono riportati nella Tabella 10.

Tabella 10. Barriere percepite alla implementazione delle schede EWS

Barriere percepite all'implementazione delle schede EWS (n = 107)		
	n	(%)
Sovrapposizione con utilizzo del partogramma	46	43.4
Mancanza di istruzioni / training (n = 106)	23	21.7
Personale insufficiente per completare adeguatamente le schede MEOWS	21	19.6
Mancanza di supporto per le schede MEOWS da parte delle Ostetriche	18	16.8
Mancanza di supporto per le schede MEOWS da parte dei medici	17	15.9
Utilizzo di altre schede parametri	15	14
Mancanza di evidenza e validazione delle MEOWS nell'assistenza ostetrica	14	13.1
Mancanza di priorità relativamente ad altre procedure assistenziali	13	12.1
Altro	13	12.1
Necessario troppo tempo	10	9.3
Parametri trigger inappropriati per la gravida	9	8.4
Impatto sulla donna delle frequenti interruzioni	4	3.7

Nel 21% dei casi sono stati riferiti dei ritardi alla chiamata del medico, in genere dovuti all'elevato carico di lavoro. Spesso non vi è una chiara delineazione del processo di intensificazione del monitoraggio e del coinvolgimento di un'equipe multidisciplinare. La barriera più importante rilevata è stata la sovrapposizione con la compilazione del partogramma (43%). Ricordiamo che la

scheda MEOWS non è validata per la donna in travaglio. Pertanto, per il monitoraggio della donna in travaglio ci si deve attenere al protocollo che non prevede l'utilizzo delle schede MEOWS.

La mancanza di indicazioni / training sull'utilizzo della scheda MEOWS è stato il secondo ostacolo riconosciuto (22%) al quale si aggiunge la mancanza di personale.

L'implementazione dell'utilizzo della scheda MEOWS richiede un percorso formativo per ginecologi, ostetriche ed anestesisti, in merito alla modalità corretta di rilevamento dei parametri e alla modalità di intensificazione del monitoraggio con il modificarsi dei parametri. Inoltre, è necessario avviare audit, feedback e revisione di casi clinici.

Allegato 4. Scelta del regime antibiotico, rimodulazione e durata del trattamento

Tabella 11. Agenti patogeni più frequentemente coinvolti nelle sepsi in ambito ostetrico a seconda del focolaio infettivo di partenza (da Joseph et al. 2009).

Infezione che può determinare sepsi e microrganismi più frequentemente coinvolti (indicati tra parantesi)	Microrganismi che più frequentemente sono causa di sepsi in gravidanza/ puerperio
Pielonefrite (1, 4)	1 *Escherichia coli*
Ascesso perinefrico (1, 4)	2 *Bacteroides* (anaerobi)
Polmonite (6, 7)	3 *Clostridium* (anaerobi)
Corioamniosite (1, 2, 8–12)	4 *Klebsiella* spp.
Endometrite (1, 2, 5, 9, 12)	5 *Pseudomonas aeruginosa*
Fascite necrotizzante (2, 3, 6, 9)	6 *Streptococcus* species
Aborto settico (1, 3)	7 *Staphylococcus aureus*
Infezione di ferita da TC (1, 2, 6, 7)	8 Streptococco di Gruppo B
Mastite (7)	9 *Peptostreptococcus* (anaerobi)
	10 *Enterococcus* spp.
	11 *Listeria monocytogenes*
	12 *Enterobacter* spp.

Tabella 12. Agenti patogeni isolati in donne con sepsi nelle diverse fasi della gravidanza (da Knowles et al. 2015).

	In gravidanza	Intrapartum	Postpartum
E. coli	55%	22%	42%
Streptococco Gruppo B	4.2%	43%	9.2%
Anaerobi	8.5%	8%	8.5%
Stafilococco	8.5%	5%	9.2%
Enterococco	4.2%	5%	4.6%
Streptococco Gruppo A	0	2%	7.6%
Klebsiella	2%	2%	1.5%
H. influenzae	6.4%	1%	0
Altri	11.2%	11%	11.2%
**Totale, n**	**47**	**99**	**130**

Tabella 13. Dosaggi raccomandati e modalità di infusione per gli antibiotici comunemente utilizzati nella sepsi.

ANTIBIOTICO	DOSE STANDARD E.V. NELL'ADULTO CON DIAGNOSI DI SEPSI
Amoxicillina/clavulanato	2.25 g ogni 6 h
Amikacina	15 mg/Kg/die
Ampicillina /sulbactam	3 g ogni 6 h
Azitromicina	500 mg/die (unica somministrazione)
Ceftriaxone	2 g/die (unica somministrazione)
Clindamicina	900 mg ogni 8 h
Ciprofloxacina	400 mg ogni 8-12 h
Daptomicina	8 mg/kg/die (unica somministrazione)
Gentamicina	5 mg/kg/die (unica somministrazione)
Levofloxacina	750 mg/die (se peso > 70 Kg : 500 mg ogni 12h)
Meropenem	2g dose carico, quindi 1 g ogni 6-8 h (infusione prolungata di 4-6 h)
Metronidazolo	15 mg/kg dose di carico, quindi 7.5 mg/kg ogni 6 h
Piperacillina/tazobactam	4,5 g ogni 6 h (prima dose in bolo, poi infusione prolungata di 4-6 h)
Vancomicina	25 mg/kg dose di carico, poi 500 mg ogni 6 h

Possibili schemi (Tabella 7 e Tabella 13) per la terapia antibiotica empirica iniziale della sepsi in ambito ostetrico includono un carbapenemico ad ampio spettro (meropenem; imipenem/cilastatina; ertapenem; doripenem) o una combinazione penicillina/inibitore delle beta lattamasi (es. Piperacillina/tazobactam) in associazione ad un aminoglicoside (es. gentamicina; amikacina). La terapia combinata (utilizzare 2 diverse classi di antibiotico in associazione) sembra biologicamente plausibile e probabilmente utile dal punto di vista clinico nel caso di infezione severa (in particolare nello shock settico) anche se non vi sono evidenze conclusive di beneficio nel caso di batteriemia e sepsi senza shock. Tuttavia, in pazienti con sepsi da Gram negativi (patogeni prevalenti nelle sepsi in gravidanza e puerperio) è stato riportato che la combinazione beta-lattamico piu aminoglicoside riduce in maniera significativa la probabilità di terapia iniziale inadeguata, che rappresenta un fattore predittivo indipendente di mortalità (Micek 2010).
L'aumento dei batteri Gram negativi resistenti ai carbapenemi (European Centre for Disease Prevention and Control 2017) impone di limitare l'uso di tali antibiotici solo nelle infezioni complicate da batteri produttori di ß-lattamasi a spettro esteso -ESBL- (pazienti con una recente infezione da batteri ESBL accertata microbiologicamente, con una condizione di colonizzazione da batteri ESBL o con una recente esposizione a fluorochinoloni e/o cefalosporine). In assenza di queste condizioni è preferibile l'associazione di un beta-lattamico + aminoglicoside per la terapia empirica della sepsi in ostetricia (Elton and Chaudhari, 2015; Burton and Sibai 2012; Rhodes 2016). La dose iniziale di beta-lattamico può essere somministrata in bolo o infusione rapida per raggiungere rapidamente i livelli ematici terapeutici. Dopo la dose iniziale, sembra che un'infusione protratta/continua sia più efficace rispetto alla somministrazione a dosi intermittenti.
Per gli aminoglicosidi, in caso di insufficienza renale è necessario monitorare il livello plasmatico del farmaco per assicurarsi che le concentrazioni siano sufficiente basse da minimizzare il rischio di tossicità renale e per calibrare l'intervallo di tempo tra le somministrazioni.

Per la possibilità di cross-reazione tra penicilline e carbapenemici (stimata in circa l'1% dei casi), in caso di shock anafilattico o reazione allergica grave a penicillina in anamnesi, i carbapenemici sono controindicati. In queste pazienti, una possibile alternativa è rappresentata dall'associazione ciprofloxacina + aminoglicoside.
Gli schemi terapeutici sopra suggeriti non sono appropriati in caso di infezione da *S. aureus* meticillino-resistente (MRSA). In Italia il fenomeno delle infezioni comunitarie da MRSA è ritenuto decisamente più contenuto rispetto allo scenario statunitense, mentre è alta la prevalenza di MRSA nelle infezioni nosocomiali. Non esistendo uno score specifico per la valutazione del rischio di colonizzazione da MRSA in setting ostetrico, si può considerare la possibilità di colonizzazione da MRSA in presenza di uno o più dei seguenti fattori di rischio:

- esposizione a precedente terapia antibiotica (entro 6 mesi) con fluorochinoloni, cefalosporine, carbapenemi;
- ospedalizzazione o terapia endovenosa nei 12 mesi precedenti al parto;
- trasferimento da altra struttura sanitaria;
- presenza di catetere vescicale o cateteri vascolari al momento dell'accesso in ospedale.

In questi casi, va aggiunto allo schema terapeutico suggerito sopra un farmaco anti-MRSA, come vancomicina, daptomicina o teicoplanina

In Tabella 14 sono riportati i dati di sicurezza sui principali antibiotici utilizzati nella gestione della sepsi per l'impiego in gravidanza (si fa riferimento alla classificazione della Food and Drug Administration) e durante l'allattamento (dati tratti dal database LACTMED).

La **rimodulazione del trattamento**, soprattutto quando si imposta inizialmente una terapia antibiotica combinata, è di beneficio sia a livello delle società perché riduce i rischi di sviluppare resistenze batteriche, ma anche per il singolo paziente (rischi di sovrainfezioni e mortalità). Le pazienti che iniziano terapia antibiotica empirica per sepsi o shock settico dovranno essere valutate successivamente, idealmente dopo le prime 72 ore, dallo specialista infettivologo per la rimodulazione della terapia. Una volta disponibili gli esiti degli esami colturali, eliminare gli antibiotici non necessari e rimpiazzare gli antibiotici ad ampio spettro con agenti più specifici. La valutazione finalizzata a rimodulare la terapia antibiotica deve essere fatta quotidianamente.

Durata del trattamento: una durata del trattamento antibiotico di 7-10 giorni è adeguata per la maggior parte delle infezioni associate con sepsi o shock settico. Trattamenti più prolungati sono opportuni per le pazienti con una risposta clinica lenta, con focolai di infezione non drenabili, batteriemie o nelle pazienti con immunodeficienza. Trattamenti più brevi sono appropriati nelle pazienti con rapida risoluzione clinica dopo controllo del focolaio di partenza nelle sepsi addominali o urinarie. In caso di batteriemia è raccomandato l'effettuazione di 2 set di emocolture a 48-72 ore dalle prime emocolture positive per documentare la negativizzazione delle emocolture ed il controllo dell'infezione.

Tabella 14. Dati di sicurezza per l'impiego degli antibiotici in gravidanza e allattamento

ANTIBIOTICO	Gravidanza (categoria FDA*)	Allattamento (sommario tratto dal database LACTMED)
Clindamicina	**B**	Monitorare il neonato per effetti collaterali gastroenterici (diarrea, candidosi o raramente melena)
Ciprofloxacina	**C**	Accettabile in allattamento, ma consigliato monitoraggio del neonato per alterazioni della flora intestinale (diarrea o candidiasi). Lasciando un intervallo di 3-4 h tra somministrazione del farmaco e poppatta riduce l'esposizione del neonato al farmaco.
Daptomicina	**B**	Informazioni limitate, ma effetti avversi sul neonato non attesi per alto peso molecolare, elevato legame alle proteine e bassa biodisponibilitòà orale del farmaco.<br>Non necessarie precauzioni particolari.
Gentamicina	**D**	Considerato compatibile con l'allattamento
Meropenem	**B**	Nonostante l'assenza di informazioni sull'uso di Meropenem durante l'allattamento, non ci si aspetta che I beta-lattamici possano causare effetti avversi nel lattante. E' suggerito il monitoraggio del neonato per alterazioni della flora intestinale.
Metronidazolo	**B**	Vecchi studi avevano ipotizzato una possibile proprietà mutagena del farmaco nell'uomo, sollevando timori sull'esposizione dei neonati mediante l'allattamento. La rilevanza di questi risultati è stata messa in discussione e non esistono studi conclusivi nella specie umana. Alcune fonti raccomandano la sospensione dell'allattamento 12-24 ore dopo la somministrazione.
Piperacillina/tazobactam	**B**	Considerato compatibile con l'allattamento
Vancomicina	**C**	Dati limitati indicano che la vancomicina si ritrova a basse concentrazioni nel latte materno e poichè la biodisponibilità orale del farmaco è bassa, è improbabile che possano esserci effetti avversi sul neonato.<br>Non necessarie precauzioni particolari.
** Categoria B: **gli studi sugli animali non hanno dimostrato rischi per il feto, ma non ci sono studi metodologicamente validi e controllati nelle donne in gravidanza OPPURE gli studi sugli animali hanno rilevato una tossicità, che non è stata confermata da studi metodologicamente validi e controllati in donne al primo e ai trimestri successivi di gravidanza.***<br>*Categoria C: **gli studi sugli animali hanno rilevato una tossicità per il feto e non ci sono studi metodologicamente validi e controllati sull'uomo, tuttavia i potenziali benefici del farmaco potrebbero giustificarne l'utilizzo nella donna in gravidanza nonostante i potenziali rischi per il feto.***		

Categoria D: ***gli studi sull'uomo e i dati di farmacovigilanza hanno evidenziato un rischio per il feto, tuttavia i potenziali benefici del farmaco potrebbero giustificarne l'utilizzo nella donna in gravidanza nonostante i potenziali rischi per il feto.***

ALLEGATO 5. Check-list logistico organizzativa

Considerato che ogni organizzazione ha già in uso una Check-list Logistico Organizzativa, già proposta dal Decreto Regionale n. 7517 del 5/8/2013 "Strategie integrate per ridurre la mortalità ospedaliera associata a Sepsi grave", si ritiene importante continuare con tale monitoraggio individuando specifici indicatori (Indicatori di struttura, di processo e di esito).
Tra gli indicatori di processo l'allegata Check-list Logistico Organizzativa è stata modificata includendo gli aspetti organizzativi proposti dal presente documento specifici per i punti nascita.
L'utilizzo di tale strumento permette la verifica di tali aspetti, l'analisi delle criticità e l'individuazione delle possibili soluzioni correttive (es. rimozioni di barriere).
Rappresenta inoltre un valido strumento di benchmarking per le aziende sanitarie regionali, permettendo di creare un confronto sistematico e la creazione di uno standard di eccellenza in relazione alle performance, valutando il corretto impiego delle risorse e definirne quindi i cambiamenti necessari ai fini del miglioramento.

Azienda_______________________________ **Presidio**____________________________________

CHECK-LIST LOGISTICO ORGANIZZATIVA

Percorso clinico assistenziale

1) Esiste nel vostro Ospedale una procedura aziendale codificata per l'inquadramento clinico dei pazienti al momento del ricovero (in PS e nei reparti degenza) con contestuale definizione dell'algoritmo decisionale (criteri e soglia di allerta, tempi di rivalutazione)?

SI ☐ **NO** ☐

se SI, è previsto l'uso di uno specifico sistema a punteggio (score di allertamento tipo MEWS)?

SI ☐ NO ☐

Se SI, indicare in quali reparti: PS☐ o reparti degenza☐; quale score:.....................

Se SI, è prevista una rivalutazione dello score in tempi codificati? SI☐ NO☐

Se SI, è prevista la possibilità per gli infermieri/ostetriche di eseguire manovre diagnostico-terapeutiche

secondo i protocolli e conferma del medico di guardia? SI☐ NO

1. **bis) E' in uso nel vostro (Punto Nascita) reparto/ospedale una procedura codificata per l'inquadramento clinico e il monitoraggio con contestuale definizione dell'algoritmo decisionale (tipo score MEOWS) per la paziente ostetrica al di fuori della fase del travaglio?**

a) SI NO

Se SI

b) E' previsto l'uso di uno specifico sistema a punteggio/colore (Tipo MEOWS)?

- MEOWS (specificare quale versione) SI NO....................☐
- altro (specificare) SI NO...................☐;

c) Non Pertinente

☐

2) Esiste all'interno del vostro Ospedale un percorso clinico-assistenziale specifico per l'identificazione del paziente settico?

SI ☐ **NO** ☐

se SI☐

a) E' in uso un programma formativo per la sua condivisione? SI ☐ NO ☐

b) i relativi protocolli sono di tipo:
- cartaceo SI☐ NO
se SI, disponibile in reparto ☐ altrove☐
- supporto informatico SI☐ NO☐

2 bis) E' in uso nel vostro reparto/ospedale un protocollo diagnostico-terapeutico-assistenziale (PDTA) specifico per la sepsi ostetrica disponibile per tutti gli operatori sanitari del punto nascita?

a) SI NO

Se **SI**

b) Qual è la modalità di consultazione dei protocollo?
- cartaceo SI☐ NO☐;
se SI, disponibile in reparto ☐ altrove ______________________________☐
- supporto informatico SI☐ NO☐;
se SI, aperto a tutti -☐ ad accesso ristretto____________________________
- data ultimo aggiornamento:…………………..

3) Esiste nel vostro Ospedale una procedura di attivazione del team dell'emergenza in caso di necessità?

SI ☐ **NO**

(sintetica descrizione della procedura)

____
____
____
____

Laboratorio

4) Il vostro Laboratorio di Microbiologia accetta ed elabora emocolture h24 7 giorni su 7?

SI ☐ **NO** ☐

se NO: con che modalità le accetta ed elabora (giorni e orari)?

descrizione____
____
____

5) Sono in uso istruzioni per la conservazione delle emocolture prima dell'accettazione da parte del laboratorio?

SI ☐ **NO** ☐

6) E' in uso all'interno del vostro Ospedale una procedura aziendale per l'esecuzione delle emocolture?

SI ☐ **NO** ☐

se SI, a) indicare data stesura protocollo: …/…./…….; data ultimo aggiornamento: …/…./……;

b) protocollo di tipo
- cartaceo SI☐ NO☐;
se SI, disponibile in reparto ☐ altrove☐
- supporto informatico SI☐ NO☐

7) E' previsto che il vostro Laboratorio di Microbiologia fornisca a) un allerta immediato in caso di positività dell'esame con relativo esame batterioscopico e colorazione di Gram, e (b) una risposta preliminare negativa qualora l'emocoltura risultasse ancora negativa a 48h?

a) SI ☐ **NO**

b) SI **NO**☐

8) Il vostro Laboratorio di Biochimica è in grado accettare ed elaborare esami in urgenza 7 giorni su 7 h 24 ?

SI ☐ **NO** ☐
se NO: con che modalità gli accetta ed elabora (giorni e orari)?
____
____
____

9) E' possibile la misurazione dei lattati h24 in urgenza in tutti i reparti di degenza (point of care e/o laboratorio centrale)?
SI ☐ **NO**☐ (se parziale, specificare)☐____

10) Quanto tempo richiede in media l'esito della misura dei lattati ?
- in Pronto soccorso: immediato☐ <1hr☐ >1h☐ <2hr☐ >2hr☐
- in area di degenza: immediato☐ <1hr☐ >1h☐ <2hr☐ >2hr☐

Accesso venoso centrale
11) Esiste la possibilità di posizionare un catetere venoso centrale h24 in tutti i reparti di degenza medica?
SI ☐ **NO** ☐
se NO, indica se richiede trasferimento in: PS o Terapia intensiva SI☐ NO☐
camera operatoria: SI☐ NO☐

Pompe infusionali
12) Esiste la disponibilità di pompe infusionali in tutti i reparti?
SI ☐ **NO** ☐
se NO, indicare in quali reparti: PS☐ o reparti di degenza
☐
Urinometri
13) Esiste la disponibilità di urinometri in tutti i reparti?
SI ☐ **NO** ☐
se NO, indicare in quali reparti: PS☐ o reparti di degenza

Monitoraggio volemico non invasivo
14) Esiste la possibilità di avere un monitoraggio non invasivo con ecografia dello stato volemico (e della responsività) al letto del paziente in tutti i reparti?
SI ☐ **NO** ☐
se NO, indicare in quali reparti: PS☐ o reparti di degenza☐(nota)

Antibioticoterapia
15) Avete a disposizione delle linee guida aziendali di antibioticoterapia <u>empirica?</u>
SI ☐ **NO** ☐
se SI, è in uso un programma formativo per la condivisione? SI☐ NO☐
se SI, a) indicare: data stesura protocollo: …/…./…….; data ultimo aggiornamento: …/…./……;
b) qual è la modalità di consultazione dei protocolli?
- cartaceo SI☐ NO☐;
se SI, disponibile in reparto ☐ altrove☐
- supporto informatico SI☐ NO☐

15 bis)) Avete a disposizione delle linee guida di antibioticoterapia empirica per la paziente ostetrica?
a) SI NO
se SI, è in uso un programma formativo per la condivisione? SI☐ NO☐
se SI, a) indicare: data stesura protocollo: …/…./…….; data ultimo aggiornamento: …/…./……;
b) qual è la modalità di consultazione dei protocolli?
- cartaceo SI☐ NO☐;
se SI, disponibile in reparto ☐ altrove☐

- supporto informatico SI☐ NO☐;
  se SI, aperto a tutti☐ ad accesso ristretto
- data ultimo aggiornamento: …………………

16) Quali categorie di questi antibiotici avete sempre a disposizione nel vostro PS?
Chinolonici SI☐ NO☐, Carbapenemici SI☐ NO☐, Glicopeptidi SI☐ NO☐
Macrolidi SI☐ NO☐, Metronidazolo SI☐ NO☐, Cefalosporine III SI☐ NO
☐

17) Quali categorie di questi antibiotici avete sempre a disposizione nei vostri reparti di degenza medica?
Chinolonici SI☐ NO☐, Carbapenemici SI☐ NO☐, Glicopeptidi SI☐ NO☐
Macrolidi SI☐ NO☐, Metronidazolo SI☐ NO☐, Cefalosporine III SI☐ NO☐

17.1) Quali categorie di questi antibiotici avete sempre a disposizione **in Ostetricia**?
Associazione penicilline/inibitori beta lattamasi SI☐ NO , Macrolidi SI☐ NO☐,
Glicopeptidi SI☐ NO Chinolonici SI☐ NO☐

18) Dovete effettuare una richiesta motivata per alcuni antibiotici?
SI ☐ **NO** ☐
se SI, è possibile comunque rifornirvi nei festivi o durante la notte? SI☐ NO☐

Controllo della fonte

19) Con quale modalità è possibile attivare la consulenza del Chirurgo? Guardia attiva☐o reperibile☐;
Con quale modalità è possibile attivare la consulenza dell' Infettivologo? Guardia attiva☐o reperibile☐;

20) Il consulente Infettivologo viene "routinariamente" contattato nei casi di sepsi severa e shock settico (scelta/dose Atb)?
SI ☐ **NO** ☐

21) Con quale modalità sono attivabili equipe chirurgica e sala operatoria del pronto soccorso per eradicazione fonte ?
Guardia attiva SI☐
Reperibilità SI☐

22) Avete nel vostro Ospedale dei protocolli codificati e condivisi con i Chirurghi mirati all'eradicazione della fonte settica?
SI ☐ **NO** ☐
se SI, è in uso un programma formativo per la condivisione? SI☐ NO☐
se SI, a) indicare: data stesura protocollo: …/…./…….; data ultimo aggiornamento: …/…./……;
b) qual è la modalità di consultazione dei protocolli?
- cartaceo SI☐ NO☐;
  se SI, disponibile in reparto ☐ altrove☐
- supporto informatico SI☐ NO☐

23) Avete nel vostro Ospedale dei protocolli codificati e condivisi mirati al percorso diagnostico terapeutico del paziente con sospetta meningite?
SI ☐ **NO** ☐
se SI, è in uso un programma formativo per la condivisione? SI☐ NO☐
se SI, a) indicare: data stesura protocollo: …/…./…….; data ultimo aggiornamento: …/…./……;
b) qual è la modalità di consultazione dei protocolli?
- cartaceo SI☐ NO☐;
  se SI, disponibile in reparto ☐ altrove☐

- supporto informatico SI☐ NO☐

24) Avete nel vostro Ospedale dei protocolli codificati e condivisi mirati al percorso diagnostico terapeutico del paziente con sospetta infezione del catetere venoso centrale?

SI ☐ **NO** ☐

se SI, è in uso un programma formativo per la condivisione? SI☐ NO☐

se SI, a) indicare: data stesura protocollo: …/…./…….; data ultimo aggiornamento: …/…./……;

b) qual è la modalità di consultazione dei protocolli?

- cartaceo SI☐ NO☐;

se SI, disponibile in reparto ☐ altrove☐

- supporto informatico SI☐ NO☐

25) Avete nel vostro Ospedale dei protocolli codificati e condivisi mirati al percorso diagnostico terapeutico del paziente con sospetta polmonite comunitaria?

SI ☐ **NO** ☐

se SI, è in uso un programma formativo per la condivisione? SI☐ NO☐

se SI, a) indicare: data stesura protocollo: …/…./…….; data ultimo aggiornamento: …/…./……;

b) qual è la modalità di consultazione dei protocolli?

- cartaceo SI☐ NO☐;

se SI, disponibile in reparto ☐ altrove☐

- supporto informatico SI☐ NO☐

26) Esiste nel vostro Ospedale un servizio di radiologia interventistica per eradicazione fonte?

SI ☐ **NO** ☐

se SI, l'attivazione di questo percorso è agevole? SI☐ NO☐

se NO, avete una struttura ospedaliera di riferimento/convenzionata? SI☐ NO☐

Varie

27) La refertazione degli esami radiologici è attiva 7 giorni su 7 h24 ?

SI ☐ **NO** ☐

se NO, indica fasce orarie: ____________________

se NO, indica se attivabile reperibile: SI☐ NO ☐

28) E'in uso nel vostro reparto/ospedale un sistema di sorveglianza delle infezioni/sepsi/shock settico in gravidanza/puerperio ?

a) **SI** **NO**

se SI, Specificare quale e cadenza…………………………………………………………..☐

29) Nel vostro punto nascita i fattori di rischio (dare esempi: BMI, rischio sociale, etnie, fumo, stili di vita, ecc) di infezione/sepsi una volta identificati sono sistematicamente riportati sulla cartella ostetrica o altra documentazione sanitaria e facilmente individuabili da parte di tutti gli operatori durante il percorso della paziente gravida/in puerperio?

a) **SI** **NO**

se SI, Specificare come:…………………… ....

30) Segnalare eventuali difficoltà riscontrate nel percorso diagnostico-terapeutico del paziente settico nel vostro Ospedale o commenti sulla compilazione della check-list LO:

A)____

B)____

C)____

31) E' stato individuato il Gruppo di Miglioramento Aziendale SI ☐ NO ☐

Se SI indicare da chi è composto (ruolo, funzione)

e segnalare un ***Referente*** con NOME – COGNOME – MAIL e CONTATTO TELEFONICO

a) ……………………………………………………………………………………………………..
b) ……………………………………………………………………………………………………..
c) ……………………………………………………………………………………………………..
d) ……………………………………………………………………………………………………..
e) ……………………………………………………………………………………………………..
f) ……………………………………………………………………………………………………..
g) ……………………………………………………………………………………………………..
h) ……………………………………………………………………………………………………..
i) ……………………………………………………………………………………………………..
j) ……………………………………………………………………………………………………..
k) ……………………………………………………………………………………………………..
l) ……………………………………………………………………………………………………..
m) ……………………………………………………………………………………………………..
n) ……………………………………………………………………………………………………..

Referente

Nome……………………………………………….. Cognome…………………………………………………..

e.mail ………………………………………………………………………………………………

contatto telefonico
……..

BIBLIOGRAFIA

- Acosta CD, Kurinczuk JJ, Lucas DN, Tuffnell DJ, Sellers S, Knight M; United Kingdom Obstetric Surveillance System. Severe maternal sepsis in the UK, 2011-2012: a national case-control study. PLoS Med. 2014;11:e1001672.
- AGENAS. Linee di indirizzo clinico-organizzative per la prevenzione delle complicanze legate alla gravidanza. 2017
- Agency for Healthcare Research and Quality (AHRQ) Patient Safety Indicators Technical Specifications Updates - Version 6.0, September. Disponibile in: http://www.qualityindicators.ahrq.gov/modules/PSI_TechSpec.aspx.
- Albright CM, Ali TN, Lopes V, Rouse DJ, Anderson BL. The Sepsis in Obstetrics Score: a model to identify risk of morbidity from sepsis in pregnancy. Am J Obstet Gynecol. 2014;211:39.e1-8.
- Albright CM, Has P, Rouse DJ, Hughes BL. Internal validation of the sepsis in obstetrics score to identify risk of morbidity from sepsis in pregnancy. Obstet Gynecol 2017; 130: 747-755.
- Angus DC and van der Poll T. Severe Sepsis and Septic Shock. N Engl J Med 2013; 369: 840-851
- Angus DC, Linde-Zwirbe WT, Lidicker J et Al. Epidemiology of severe sepsis in United States: Analysis of incidence, outcome, and associated costs of care. Crit Care Me 2001; 9: 1303-1310
- Bagshaw SM, Lapinsky S, Dial S, et al; Cooperative Antimicrobial Therapy of Septic Shock (CATSS) Database Research Group: Acute kidney injury in septic shock: clinical outcomes and impact of duration of hypotension prior to initiation of antimicrobial therapy. Intensive Care Med 2009; 35:871–881.
- Barton JR, Sibai BM. Severe sepsis and septic shock in pregnancy. Obstet Gynecol. 2012;12:689-706.
- Bates SM, Greer IA, Middeldorp S, Veenstra DL, Prabulos AM, Vandvik PO. VTE, thrombophilia, antithrombotic therapy, and pregnancy: Antithrombotic Therapy and Prevention of Thrombosis, 9th ed: American College of Chest Physicians Evidence-Based Clinical Practice Guidelines. Chest. 2012;141:e691S-e736S.
- Bauer ME, Bateman BT, Bauer ST et Al. Maternal Sepsis Mortality and Morbidity During Hospitalization for Delivery: Temporal Trends and Indipendent Associations for Severe Sepsis. Anesth Analg 2013; 117: 944-50.
- Berni G, Francois C, Tonelli L. National Early Warning Score (NEWS) Misurazione standardizzata della gravità della malattia. Linea guida Consiglio Sanitario Regionale Regione Toscana. Traduzione e adattamento dalla linea guida originale della Royal College of Physicians. Aggiornamento 2016.
- Bonet M, Nogueira Pileggi V, Rijken MJ, Coomarasamy A, Lissauer D, Souza JP,G√ºlmezoglu AM. Towards a consensus definition of maternal sepsis: results of a systematic review and expert consultation. Reprod Health. 2017;14:67.
- Bowyer L, Robinson HL, Barrett H, et al.: SOMANZ guidelines for the investigation and management sepsis in pregnancy.Aust NZ Obstet Gynaecol 2017;57:540-551
- Bryan CS, Reynolds KL, Moore EE. Bacteremia in obstetrics and gynecology. Obstet Gynecol 1984;64:155–8.
- Buppasiri P, Lumbiganon P, Thinkhamrop J, Thinkhamrop B. Antibiotic prophylaxis for third- and fourth-degree perineal tear during vaginal birth. Cochrane Database Syst Rev. 2014:7;(10):CD005125.

- Cantwell R, Clutton-Brock T, Cooper G, et al. Saving Mothers' Lives: Reviewing maternal deaths to make motherhood safer: 2006-2008. The Eighth Report of the Confidential Enquiries into Maternal Deaths in the United Kingdom. BJOG. 2011;118 Suppl 1:1-203.
- CEMACH 2007. The Seventh Report of the Confidential Enquiries into Maternal Deaths in the United Kingdom.
- Chongsomchai C, Lumbiganon P, Laopaiboon M. Prophylactic antibiotics for manual removal of retained placenta in vaginal birth. Cochrane Database Syst Rev. 2014:20;(10):CD004904.
- Cole MF. A modified early obstetric warning system. BJ of Midwifwery 2014;22: 862-68
- Cordioli RL, Cordioli E, Negrini R, Silva E. Sepsis and pregnancy: do we know how to treat this situation? Rev Bras Ter Intensiva. 2013;25:334-44.
- Creanga AA, Syverson C, Seed K, Callaghan WM. Pregnancy-Related Mortality in the United States, 2011-2013. Obstet Gynecol. 2017;130:366-373.
- Daniels et al. The sepsis six and the severe sepsis resuscitation bundle: a prospective observational cohort study. Emerg Med J 2011 28: 507-512.
- Dellinger RP, Levy MM, Rhodes A, et al. Surviving Sepsis Campaign: International guidelines for management of severe sepsis and septic shock: 2012. Crit Care Med 2013; 41:580–637.
- Donati S, Senatore S, Ronconi A. Maternal mortality in Italy: a record-linkage study. BJOG. 2011;118:872-9.
- Drees M, Gerber JS, Morgan Dj. Research Methods in Healthcare Epidemiology and Antimicrobial Stewardship: Use of Administrative and Surveilance Databases. Infect Control Hosp Epidemiol 2016: 1-10.
- Drugs and Lactation Database (*LactMed*). Disponibile in: https://toxnet.nlm.nih.gov/newtoxnet/lactmed.htm.
- European Centre for Disease Prevention and Control. Antimicrobial resistance surveillance in Europe 2015. Annual Report of the European Antimicrobial Resistance Surveillance Network (EARS-Net). Stockholm: ECDC; 2017.
- Fernandez-Perez ER, Salman S, Pendem S, Farmer JC. Sepsis during pregnancy. Crit Care Med 2005;33:S286–93.
- Ferrer R, Martin-Loeches I, Phillips G, et al: Empiric antibiotic treatment reduces mortality in severe sepsis and septic shock from the first hour: results from a guideline-based performance improvement program. Crit Care Med 2014; 42:1749–1755
- Ford JM, Scholefield H. Sepsis in obstetrics: cause, prevention, and treatment. Curr Opin Anaesthesiol. 2014;27:253-8.
- Geller SE, Rosenberg D, Cox SM, Brown LM, Simonsons L, Driscoll CA, Kilpatrick SJ. The continuum of maternal morbidity and mortality: Factors associated with severity. Am J Obstet Gynecol 2004, 191: 939-44.
- Giani T, Pini B, Arena F, Conte V, Bracco S, Migliavacca R; AMCLI-CRE Survey Participants, Pantosti A, Pagani L, Luzzaro F, Rossolini GM. Epidemic diffusion of KPC carbapenemase-producing Klebsiella pneumoniae in Italy: results of the first countrywide survey, 15 May to 30 June 2011. Euro Surveill. 2013;18.
- Government of South Australia. Perinatal practice guidelines, Clinuical Guidelines, Sepsis in Pregnancy 2017, CG 190.
- Henderson E, Love EJ. Incidence of hospital-acquired infections associate with caesarean section. J Hosp Infect. 1995;29:245-55.

- Hussein J, Mavalankar DV, Sharma S, D'Ambruoso L. A review of health system infection control measures in developing countries: what can be learned to reducematernal mortality. Global Health. 2011;7:14.
- Intrapartum Fetal Surveillance Clinical Guidelines. RANZCOG 2014. Disponibile in: https://www.ranzcog.edu.au/intrapartum-fetal-surveillance-clinical-guidelines.html.
- Joseph J, Sinha A, Paech M, Walters BNJ. Sepsis in pregnancy and early goal-directed therapy. *Obstetric Medicine*. 2009;2:93-99.
- Kourtis AP, Read JS, Jamieson DJ. Pregnancy and infection. N Engl J Med. 2014;370:2211-8.
- Knowles SJ, O'Sullivan NP, Meenan AM, Hanniffy R, Robson M. Maternal sepsis incidence, aetiology and outcome for mother and fetus: a prospective study. BJOG.2015;122:663-71.
- Kumar A, Roberts D, Wood KE, et al: Duration of hypotension before initiation of effective antimicrobial therapy is the critical determinant of survival in human septic shock. Crit Care Med 2006; 34:1589–1596
- Mabie WC, Barton JR, Sibai B. Septic shock in pregnancy. Obstet Gynecol 1997;90:553–61.
- Martin GS, Mannino DM, Eaton S, Moss M. The epidemiology of sepsis in the United States from 1979 through 2000. N Engl J Med 2003; 348:1546–54.
- Micek ST, Welch EC, Khan J, Pervez M, Doherty JA, Reichley RM, Kollef MH. Empiric combination antibiotic therapy is associated with improved outcome against sepsis due to Gram-negative bacteria: a retrospective analysis. Antimicrob Agents Chemother. 2010;54:1742-8.
- Ministero della Salute. Near miss ostetrici in Italia: la sepsi, l'eclampsia, l'embolia di liquido amniotico e l'emoperitoneo spontaneo - Progetto finanziato dal programma CCM 2016 del Ministero della Salute.
- Morgan J, Roberts S. Maternal Sepsis. Obstet Gynecol Clin North Am. 2013; 40 (1):69-87.
- National Clinical Effectiveness Committee (NCEC). Sepsis Management. National Clinical Guideline No. 6. Disponibile in: www.health.gov.ie/patientsafety/ncec www.hse.ie/sepsis
- National Institute for Health and Clinical Excellence. Acutely ill patients in hospital. Recognition of and response to acute illness in adults in hospital. NICE clinical guideline 50. London: NICE, 2007.
- National Institute for Health and Clinical Excellence. Pregnancy and complex social factors: a model for service provision for pregnant women with complex social factors, NICE Guideline 2010, updated 2018.
- Olvera L, Dutra D. Early recognition and management of maternal sepsis. Nurs Womens Health 2016;2:182-95.
- Parfitt SE, Bogat ML, Roth C. Sepsis in obstetrics: treatment, prognosis and prevention. Am J Matern Child Nurs 2017: 42:206-209.
- Patterson C et al. Early warning systems in the UK: variation in content and implementation strategy has implications for a NHS early warning system. Clin Med 2011; 11: 424-7.
- Plante LA. Management of sepsis and septic shock for the obstetrician-gynecologist. Obstet Gynecol Clin North Am. 2016; 43:659-678.
- Report of the National High Blood Pressare Education Program. Working group report in high blood pressure in pregnancy. Am J Obstet Gynecol 2000, 183: S1-22.
- Rhodes et al. Surviving Sepsis Campaign: International Guidelines for Management of Sepsis and Septic Shock 2016. Critical Care Medicine, 2017 ;45: 486–552.
- Robson WP, Daniel R. The Sepsis Six: helping patients to survive sepsis. Br J Nurs. 2008;17:16-21.

- Royal College of Obstetricians & Gynaecologist. Bacterial Sepsis following Pregnancy. 2012 Green-top Guideline No.64b.
- Royal College of Physician. National Early Warning Score (NEWS) Standardising the assessment of acute-illness severity in the NHS. Report of a working party July 2012.
- Shields LE, Wiesner S, Klein C., Pelletrea B, Hedrian HL. Use of maternal early warning trigger tool reduces maternal morbidity. American Journal of Obstetrics and Gynecology, 214(4), 527.
- Singer M, Deutschman CS, Seymour CW. The Third International Consensus Definitions for Sepsis and Septic Shock (Sepsis-3). JAMA 2016; 315: 801-810.
- Smaill, FM, Gyte GM. Antibiotic prophylaxis versus no prophylaxis for preventing infection after cesarean section. Cochrane Database Syst Rev, 2010(1): p. CD007482.
- World Health Organization, UNICEF, UNFPA, The World Bank. Maternal Mortality 1990 to 2008: Estimates Developed by WHO, UNICEF, UNFPA and The World Bank. Geneva: WHO, 2010.
- World Health Organization, UNICEF, UNFPA, The World Bank. Maternal Mortality 1990 to 2008: Estimates Developed by WHO, UNICEF, UNFPA and The World Bank. Geneva: WHO, 2010.
- World Health Organization. Statement on Maternal Sepsis, 2017. Disponibile in: http://www.who.int/reproductivehealth/publications/maternal_perinatal_health/maternalsepsis-statement/en/
- Zaidi AK, Huskins WC, Thaver D, Bhutta ZA, Abbas Z, Goldmann DA. Hospital-acquired neonatal infections in developing countries. Lancet. 2005;365:1175-88.
